# Dataset on cost-analysis of medication deprescribing scenarios for older adult coverage under public drug benefit programs in Canada

**DOI:** 10.1016/j.dib.2020.105842

**Published:** 2020-06-08

**Authors:** Sarah Abu Fadaleh, Jody Shkrobot, Tatiana Makhinova, Dean Eurich, Cheryl A. Sadowski

**Affiliations:** a Faculty of Pharmacy & Pharmaceutical Sciences, University of Alberta, Canada; b School of Public Health, University of Alberta, Canada

**Keywords:** Deprescriptions, Inappropriate prescribing, Costs and cost analysis, Drug costs, Pharmacy fee, Pharmacy economics

## Abstract

The dataset covers the equations and procedure used for the estimation of an older adult's total annual medication costs, across Canadian provinces and territories; detailed to report pharmacy margin, government share, and patient share. We presented a case of an older adult using 10 different medications commonly used, according to Canadian Institute for Health Information. Eight different deprescribing scenarios were created, based on recommendations from Beers Criteria and the Canadian Deprescribing Network, for the purpose of comparing the cost difference before and after each intervention on pharmacies, patients, and governments. Scenarios included: (1) Stopping an over the counter medication; (2) Discontinuation of a medication; (3) Slow taper of a potentially inappropriate medication; (4) Rapid taper of a potentially inappropriate medication; (5) Switching to safer medication; (6) Dose reduction; (7) Switching to a lower cost medication; (8) Changing from combination to a single medication. The data presented are related to the article entitled “Financial advantage or barrier when deprescribing for seniors: A case based analysis” [1]

Specifications table

**Table:** 

Subject	Pharmaceutical Science
Specific subject area	Cost analysis, Deprescribing
Type of data	Tables, Equations
How data were acquired	Electronic search of provincial and territorial websites for the Ministries of Health in Canada. When data was not available, we directly contacted the Ministry of Health. When not responsive, we contacted the provincial pharmacy association, and for some territories we contacted a local pharmacy.
Data format	Raw and analyzed data
Parameters for data collection	For medication selection we used the DIN (Drug Identification Number) as the first drug on the Alberta Interactive Drug Benefit List; If not available in other provinces’ list, then the first DIN specific to that province was used. For medication supply we used three months’ supply, for four dispenses per year. For drug cost we used maximum allowable cost, if applicable [2], [3], [4], [5], [6], [7], [8], [9], [10], [11], [12]. For dispensing fee if not fixed, then it was drug pricing related (using max government allowable fees)
Description of data collection	In each province and territory, we calculated for each medication: (1) the mark-up, for prescription medication the mark-up was obtained from ministries websites, and for over the counter medication was calculated as the difference between an established wholesaler's list price (i.e. cost to pharmacy) and the same wholesaler's published manufacturer's suggested retail price. This resulted in a 35% gross margin to the pharmacy. (2) pharmacy margin, as dispensing fee plus mark up (3) government and patient share, according to eligibility and drug cost
Data source location	Canada provinces and territories
Data accessibility	With the article
Related research article	Abu Fadaleh SM, Shkrobot J, Makhinova T, Eurich D, Sadowski CA. Financial advantage or barrier when deprescribing for seniors: A case based analysis. *Research in Social and Administrative Pharmacy* [in press] *Available at:*https://doi.org/10.1016/j.sapharm.2020.03.003

## Value of the Data

• The data provide comparison of deprescribing cost-impact on three payers: the pharmacy, the patient, and the government
• Pharmaceutical policy-makers can use this model for further research concerning deprescribing cost analysis over a wider age group.
• The dataset highlights different costing criteria among Canadian public drug programs concerning older adults’ coverage.

## 1 Data description

*The Baseline dataset,* includes cost analysis of the provided case scenario among all Canadian provinces and territories before any deprescribing intervention. The patient eligibility for coverage under provincial drug programs was based on his annual income, as previously described. [1] [See Supplementary Table, Baseline Data]

*The following eight datasets*, provide each deprescribing scenario and the following cost implications. Each scenario is calculated for each province and territory in Canada, and includes the drug costs for the patient, government, and pharmacy. [See Supplementary Tables]

*The result dataset,* includes two tables; Table 1 was generated by calculating for each of the eight scenarios the mean difference of pharmacy margin, government share and patient share from the basic medication cost before the deprescring scenarios. Table 2 was calculated as the percentages of each scenario mean difference that was generated in table-1 from the average mean in terms of pharmacy margin, government share and patient share. All the calculations are in Canadian dollars.

**Table 1:** Mean difference of 8 deprescribing scenarios from the mean basic medication cost (Canadian dollars).

Average change	Pharmacy Margin	Government share	Patient share	Total cost (Patient+ Government shares)
Base	757.78	669.21	3697.61	4366.82
Scenario 1	25.69	6.66	57.26	63.91
Scenario 2	62.17	105.54	41.30	146.84
Scenario 3	14.38	15.18	7.88	23.05
Scenario 4	24.27	35.51	−64.64	−29.13
Scenario 5	38.45	46.51	−22.85	23.66
Scenario 6	0.33	2.13	1.15	3.28
Scenario 7	220.78	19.92	3231.78	3251.70
Scenario 8	−0.29	−2.89	−0.36	−3.26

**Table 2:** National average of the cost difference for each deprescribing scenario.

	Pharmacy Margin%	Government share%	Patient share%
Scenario 1	3.39	0.99	1.55
Scenario 2	8.20	15.77	1.12
Scenario 3	1.90	2.27	0.21
Scenario 4	3.20	5.31	−1.75
Scenario 5	5.07	6.95	−0.62
Scenario 6	0.04	0.32	0.03
Scenario 7	29.14	2.98	87.40
Scenario 8	−0.04	−0.43	−0.01

## 2 Experimental design, materials, and methods

The study methods have been described previously[1]. While information regarding drug costs was accessible from provincial and territorial Ministry of Health electronic websites, some data was not available and the following calls were made to complete the required dataset:

Province: Manitoba

• Interviewee: Provincial Drug Programs at Government of Manitoba
• Main question: To confirm the mark-up

Province: Newfoundland

• Interviewee: The Pharmacy Association of Newfoundland
• Main question: To confirm the mark-up

Territories: Northern Regions (Northwest territories and Nunavut)

• Interviewee: The Department of Indigenous Services Canada
• Main question: To collect Non-Insured Health Benefits (NIHB) pricing structure

A case study representing an average older adult in Canada, with common comorbidities and medications, was developed. The case scenario and medication regimen are listed below.

*Case scenario*: male (70 years) diagnosed with hypertension, hypothyroidism, diabetes, and hypercholesterolemia. His median after-tax annual income was $56,000 (Canadian dollars).

*The patient's regimen*: metformin 1000 mg twice a day, atorvastatin 40 mg tablet daily, omeprazole 20 mg tablet daily, irbesartan/hydrochlorothiazide (HCTZ)300 mg/25 mg daily, levothyroxine 50 mcg daily, atenolol 50 mg daily, liraglutide injection 1.8 mg daily, enteric coated acetylsalicylic acid (ASAEC) 81 mg daily, calcium 500 mg/Vitamin D 1000 units twice a day, lorazepam 1 mg daily at bedtime.

**Table:** 

*Scenarios description*	
Scenario 1:	Discontinuation of OTC medication (ASAEC).
Scenario 2:	Abrupt discontinuation of medication (atorvastatin, due to leg pain)
Scenario 3:	Slow taper of a PIM (lorazepam) over 4 quarters: Q1 (90 tablets) – Q2 (45 tablets) – Q3 (30 tablets)– Q4 (0 tablets)
Scenario 4:	Rapid taper of a PIM (omeprazole changed to lower dose: Q1 (90 tablets) of 20 mg - Q2 (90 tablets) of 10 mg- Q3 and Q4 (0 tablets)
Scenario 5:	Patient switched to an alternate/safer medication (lorazepam to OTC melatonin)
Scenario 6:	Dose reduction (lorazepam 1 mg to 0.5 mg dose)
Scenario 7:	Switched to lower cost medication, (based on patient request switched liraglutide to pre-filled detemir 10unit /day (1cartridge/month)).
Scenario 8:	Changed from combination to single drug (irbesartan/HCTZ 300 mg/25 mg to irbesartan 300 mg daily).

The baseline cost and change in cost on each payer for the 8 scenarios is shown in the following Figures. All values are in Canadian dollars (Figs. 1–9).

**Fig. 1:**
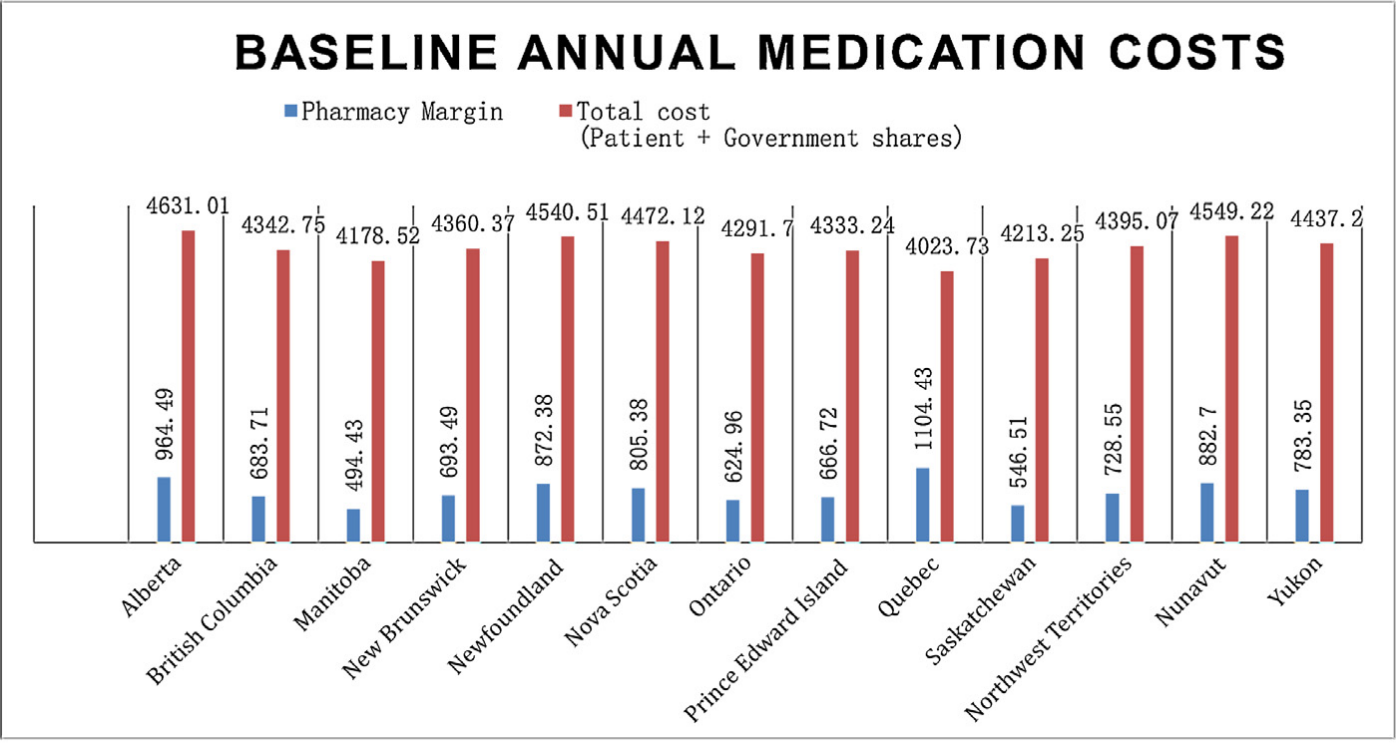
Baseline Annual Medication Costs in Canadian Dollars

**Fig. 2:**
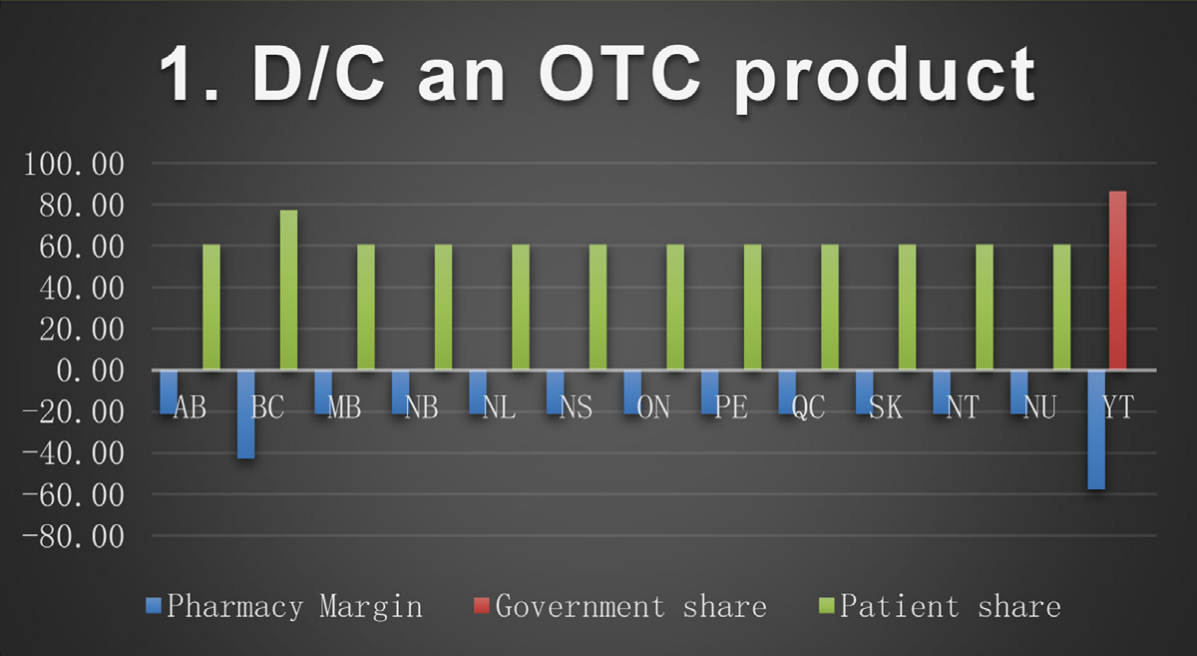
Deprescribing Scenario 1.

**Fig. 3:**
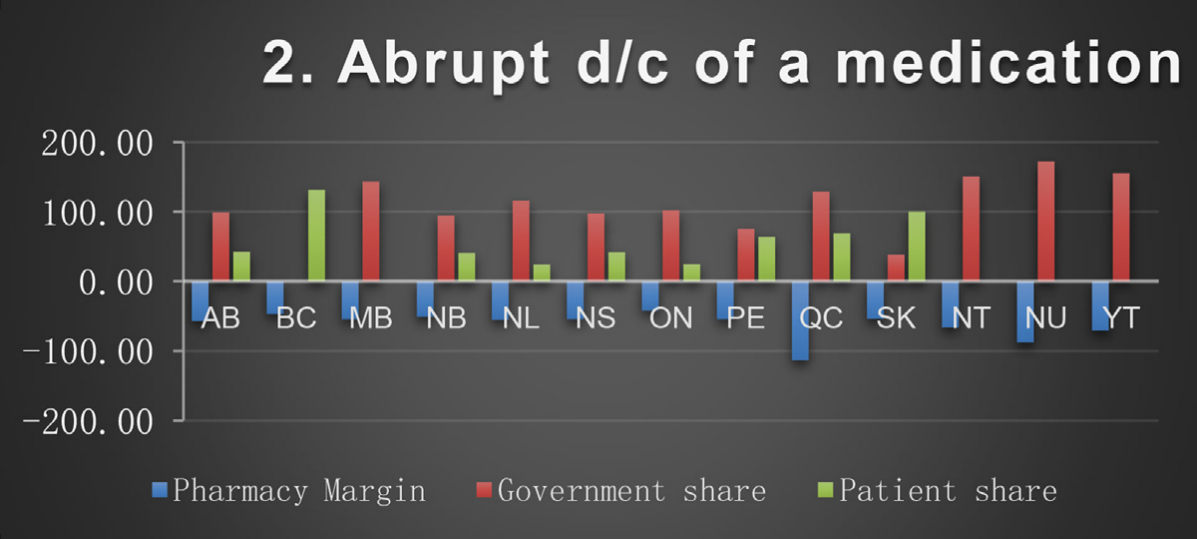
Deprescribing Scenario 2.

**Fig. 4:**
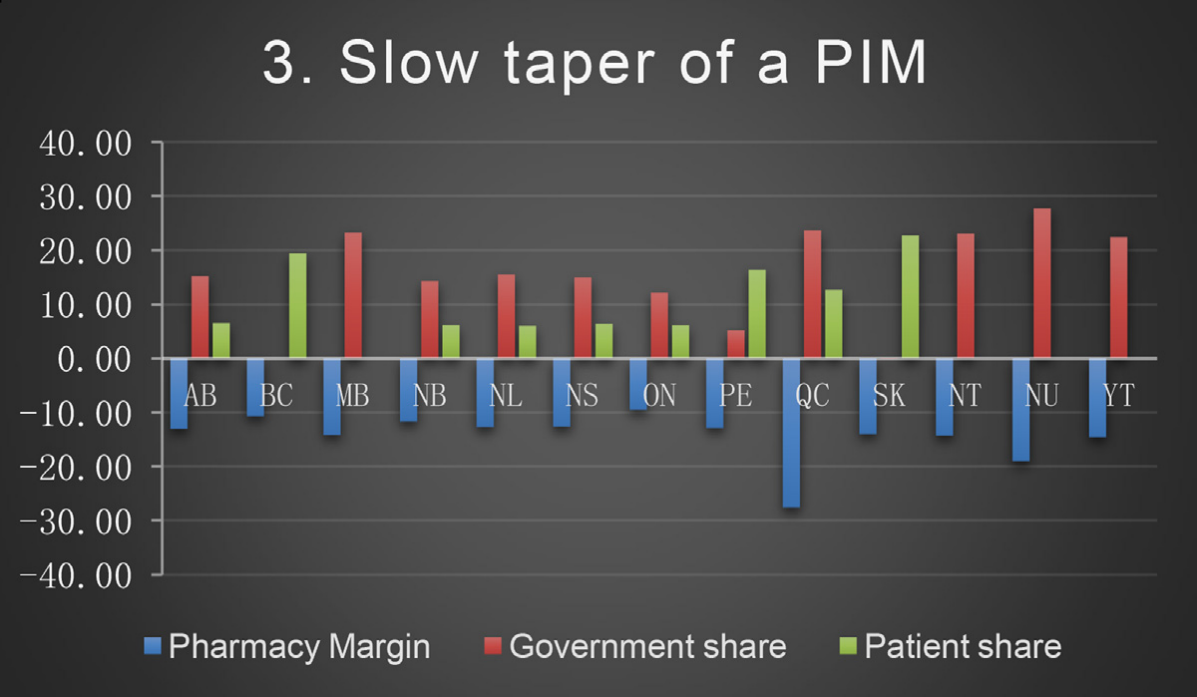
Deprescribing Scenario 3.

**Fig. 5:**
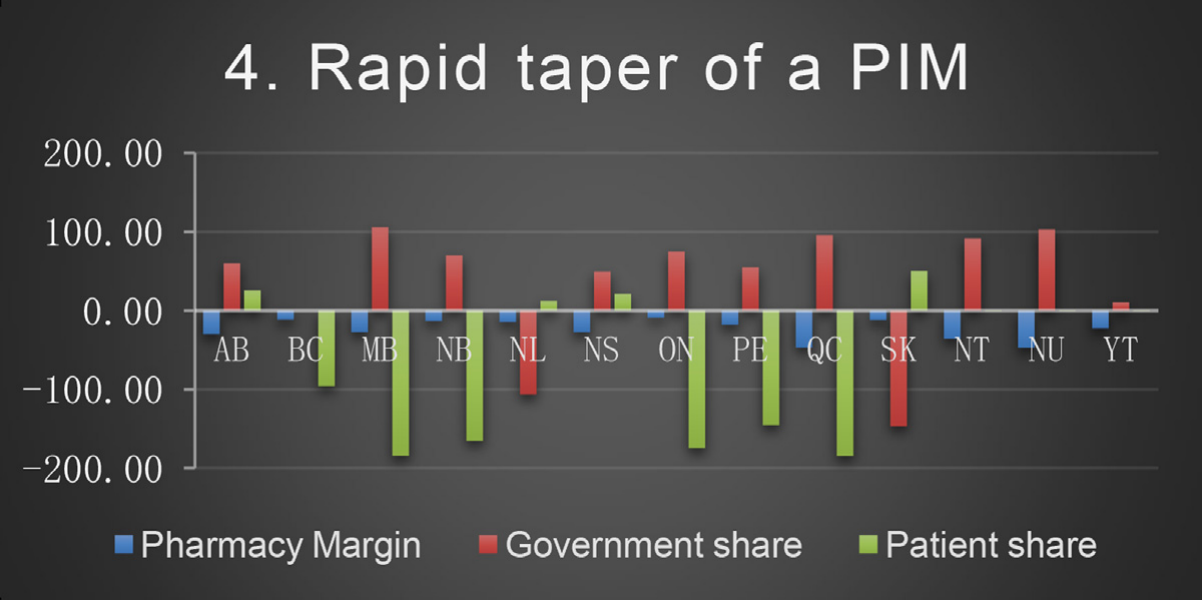
Deprescribing Scenario 4.

**Fig. 6:**
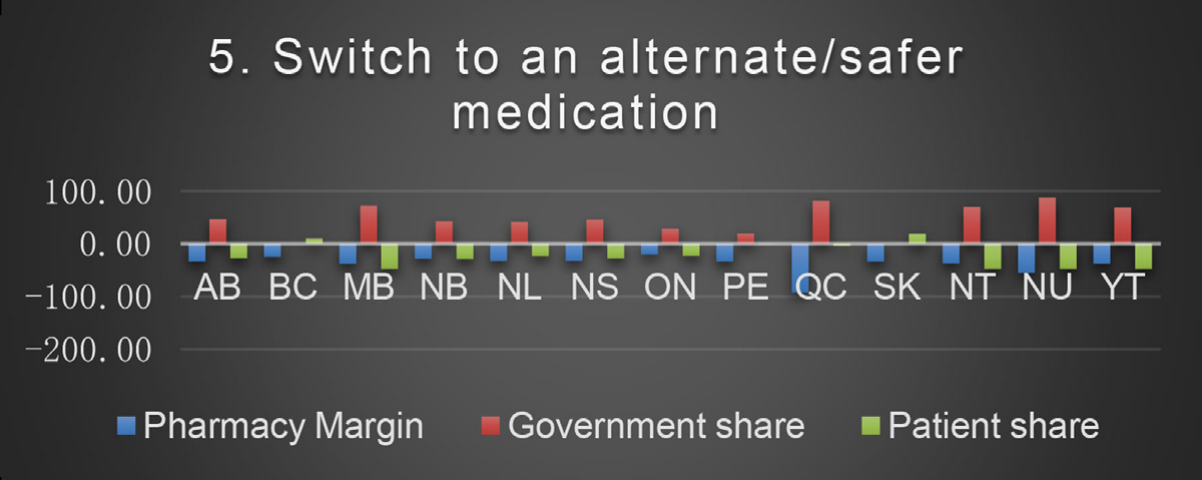
Deprescribing Scenario 5.

**Fig. 7:**
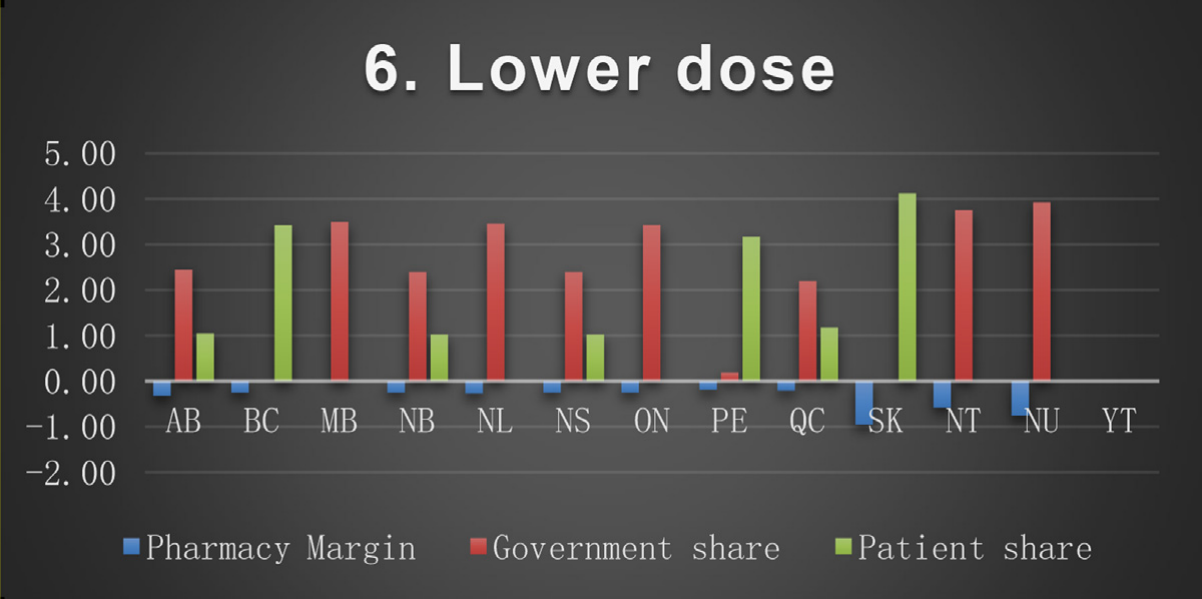
Deprescribing Scenario 6.

**Fig. 8:**
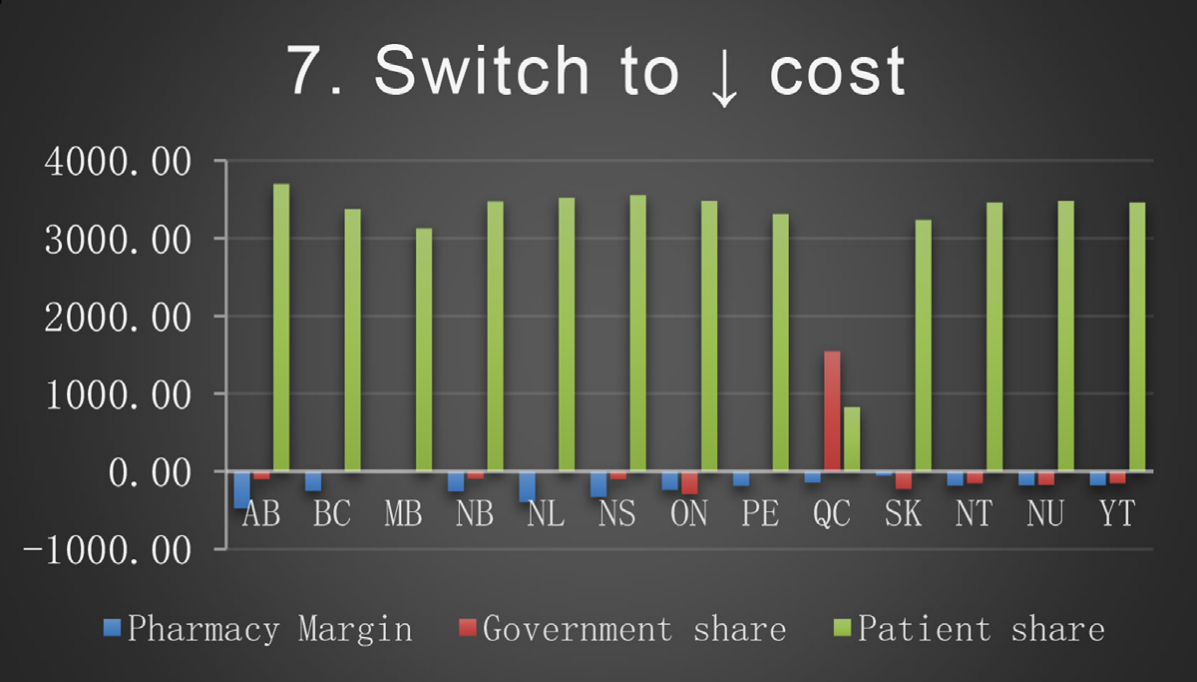
Deprescribing Scenario 7.

**Fig. 9:**
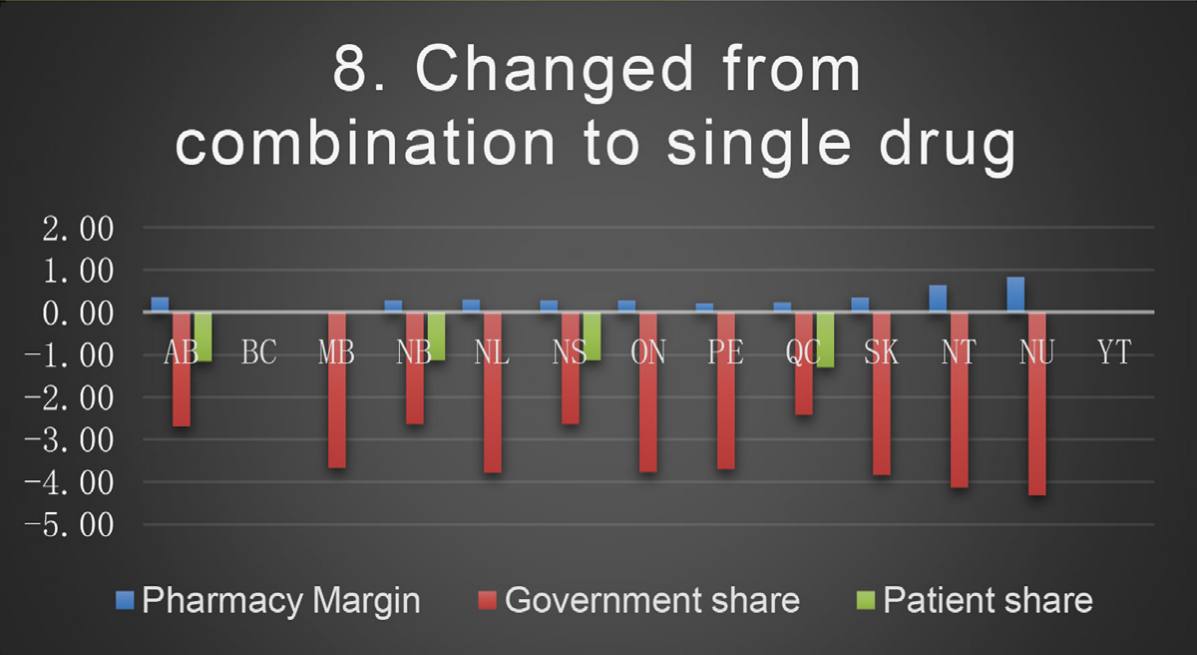
Deprescribing Scenario 8.

## Declaration of Competing Interest

The authors have no conflicts of interest to declare.

## Appendix Supplementary materials

**Supplementary materials:** Details of our method can be found in the Appendix. Image, application 1
