# Supplementary material for: Dataset on cost-analysis of medication deprescribing scenarios for older adult coverage under public drug benefit programs in Canada
Source: Data Brief. 2020 Jun 8;31:105842. doi: 10.1016/j.dib.2020.105842 (PMC7327419; doi:10.1016/j.dib.2020.105842)
Supplement: Supplementary file 1 — Supplementary materials: Details of our method can be found in the Appendix. [file mmc1.docx]

Supplementary Tables

Supplementary Table 1 – Baseline Data

| **Province** | **Drug** | **DIN** | **Days Supply** | **Quantity** | **MAC/Unit** | **Drug Cost / Unit** | **Drug Cost** | **Markup** | **Dispensing Fee** | **Total Cost** | **Pharmacy Margin $** | **Government Share** | **Patient Share** |  |  |  |
| --- | --- | --- | --- | --- | --- | --- | --- | --- | --- | --- | --- | --- | --- | --- | --- | --- |
| Alberta | Metformin 1000mg BID | 2167786 | 90 | 360 |  | 0.0247 | 8.892 | 0.9078732 | 12.15 | 21.9498732 | 13.0578732 | 15.36491124 | 6.58496196 |  |  |  |
| Alberta | Atorvastatin 40mg daily | 2295296 | 90 | 90 |  | 0.2342 | 21.078 | 2.1520638 | 12.15 | 35.3800638 | 14.3020638 | 24.76604466 | 10.61401914 |  | **Alberta /Annual costs** |  |
| Alberta | Omeprazole 20mg daily | 2245058 | 90 | 90 |  | 0.2287 | 20.583 | 2.1015243 | 12.15 | 34.8345243 | 14.2515243 | 24.38416701 | 10.45035729 |  | Pharmacy Margin | 964.4886895 |
| Alberta | Irbesartan/HCTZ 300 mg/25 mg daily | 2447894 | 90 | 90 |  | 0.2184 | 19.656 | 2.0068776 | 12.15 | 33.8128776 | 14.1568776 | 23.66901432 | 10.14386328 |  | Government share | 506.5652718 |
| Alberta | Levothyroxine 50 mcg daily | 2213192 | 90 | 90 |  | 0.031 | 2.79 | 0.284859 | 12.15 | 15.224859 | 12.434859 | 10.6574013 | 4.5674577 |  | Patient share | 4124.447818 |
| Alberta | Atenolol 50mg daily | 2255545 | 90 | 90 |  | 0.1107 | 9.963 | 1.0172223 | 12.15 | 23.1302223 | 13.1672223 | 16.19115561 | 6.93906669 |  |  |  |
| Alberta | Liraglutide inj 1.8mg daily | 2351064 | 90 | 27 |  | 29.7367 | 802.8909 | 120.6343577 | 12.15 | 935.6752577 | 132.7843577 | 0 | 935.6752577 |  | Average total cost | 463.101309 |
| Alberta | Lorazepam 1mg QHS | 655759 | 90 | 90 |  | 0.0447 | 4.023 | 0.4107483 | 12.15 | 16.5837483 | 12.5607483 | 11.60862381 | 4.97512449 |  |  |  |
| Alberta | ASAEC 81mg daily | 2237726 | 90 | 90 |  | 0.1095 | 9.855 | 5.306538462 | 0 | 15.16153846 | 5.306538462 | 0 | 15.16153846 |  |  |  |
| Alberta | Calcium 500mg / Vitamin D 1000U BID | 80017748 | 90 | 180 |  | 0.09389 | 16.9002 | 9.100107692 | 0 | 26.00030769 | 9.100107692 | 0 | 26.00030769 |  |  |  |
| Alberta/ Total Q1 |  |  |  |  |  |  |  |  |  | 115.7753272 | 241.1221724 | 126.641318 | 1031.111954 |  |  |  |
| British Columbia | Metformin 1000mg BID | 2167786 | 90 | 360 | 0.0267 |  | 8.9 | 0.712 | 10 | 19.612 | 10.712 | 0 | 19.612 |  |  |  |
| British Columbia | Atorvastatin 40mg daily | 2295296 | 90 | 90 | 0.2529 |  | 21.075 | 1.686 | 10 | 32.761 | 11.686 | 0 | 32.761 |  | **British Columbia /Annual costs** |  |
| British Columbia | Omeprazole 20mg daily | 2245058 | 90 | 90 | 0.2025 |  | 16.875 | 1.35 | 10 | 28.225 | 11.35 | 0 | 28.225 |  | Pharmacy Margin | 683.7148521 |
| British Columbia | Irbesartan/HCTZ 300 mg/25 mg daily | 2447894 | 90 | 90 | 0.2719 |  | 22.65833333 | 1.812666667 | 10 | 34.471 | 11.81266667 | 0 | 34.471 |  | Government share | 0 |
| British Columbia | Levothyroxine 50 mcg daily | 2213192 | 90 | 90 | 0.0341 |  | 2.841666667 | 0.227333333 | 10 | 13.069 | 10.22733333 | 0 | 13.069 |  | Patient share | 4342.745919 |
| British Columbia | Atenolol 50mg daily | 2255545 | 90 | 90 | 0.1196 |  | 9.966666667 | 0.797333333 | 10 | 20.764 | 10.79733333 | 0 | 20.764 |  |  |  |
| British Columbia | Liraglutide inj 1.8mg daily | 2351064 | 90 | 27 |  | 29.7367 | 802.8909 | 64.231272 | 10 | 877.122172 | 74.231272 | 0 | 877.122172 |  |  |  |
| British Columbia | Lorazepam 1mg QHS | 655759 | 90 | 90 | 0.0483 |  | 4.025 | 0.322 | 10 | 14.347 | 10.322 | 0 | 14.347 |  | Average total cost | 434.2745919 |
| British Columbia | ASAEC 81mg daily | 2237726 | 90 | 90 | 0.1035 |  | 8.625 | 0.69 | 10 | 19.315 | 10.69 | 0 | 19.315 |  |  |  |
| British Columbia | Calcium 500mg / Vitamin D 1000U BID | 80017748 | 90 | 180 |  | 0.09389 | 16.9002 | 9.100107692 | 0 | 26.00030769 | 9.100107692 | 0 | 26.00030769 |  |  |  |
| British Columbia/ Total Q1 |  |  |  |  |  |  |  |  |  | 108.568648 | 170.928713 | 0 | 1085.68648 |  |  |  |
| Manitoba | Metformin 1000mg BID | 2167786 | 90 | 360 |  | 0.0259 | 9.324 | 0 | 13.65 | 22.974 | 13.65 | 22.974 | 0 |  |  |  |
| Manitoba | Atorvastatin 40mg daily | 2295296 | 90 | 90 |  | 0.2459 | 22.131 | 0 | 13.65 | 35.781 | 13.65 | 35.781 | 0 |  | **Manitoba /Annual costs** |  |
| Manitoba | Omeprazole 20mg daily | 2245058 | 90 | 90 |  | 0.2401 | 21.609 | 0 | 13.65 | 35.259 | 13.65 | 35.259 | 0 |  | Pharmacy Margin | 494.4265846 |
| Manitoba | Irbesartan/HCTZ 300 mg/25 mg daily | 2447894 | 90 | 90 |  | 0.2293 | 20.637 | 0 | 13.65 | 34.287 | 13.65 | 34.287 | 0 |  | Government share | 747.708 |
| Manitoba | Levothyroxine 50 mcg daily | 2213192 | 90 | 90 |  | 0.031 | 2.79 | 0 | 13.65 | 16.44 | 13.65 | 16.44 | 0 |  | Patient share | 3430.810985 |
| Manitoba | Atenolol 50mg daily | 2255545 | 90 | 90 |  | 0.1162 | 10.458 | 0 | 13.65 | 24.108 | 13.65 | 24.108 | 0 |  |  |  |
| Manitoba | Liraglutide inj 1.8mg daily | 2351064 | 90 | 27 |  | 29.7367 | 802.8909 | 0 | 13.65 | 816.5409 | 13.65 | 0 | 816.5409 |  |  |  |
| Manitoba | Lorazepam 1mg QHS | 655759 | 90 | 90 |  | 0.0492 | 4.428 | 0 | 13.65 | 18.078 | 13.65 | 18.078 | 0 |  |  |  |
| Manitoba | ASAEC 81mg daily | 2237726 | 90 | 90 |  | 0.1095 | 9.855 | 5.306538462 | 0 | 15.16153846 | 5.306538462 | 0 | 15.16153846 |  |  |  |
| Manitoba | Calcium 500mg / Vitamin D 1000U BID | 80017748 | 90 | 180 |  | 0.09389 | 16.9002 | 9.100107692 | 0 | 26.00030769 | 9.100107692 | 0 | 26.00030769 |  |  |  |
| Manitoba/ Total Q1 |  |  |  |  |  |  |  |  |  |  | 123.6066462 | 186.927 | 857.7027462 |  |  |  |
| New Brunswick | Metformin 1000mg BID | 2167786 | 90 | 360 | 0.0247 |  | 8.892 | 0.71136 | 11 | 20.60336 | 11.71136 | 14.422352 | 6.181008 |  |  |  |
| New Brunswick | Atorvastatin 40mg daily | 2295296 | 90 | 90 | 0.2342 |  | 21.078 | 1.68624 | 11 | 33.76424 | 12.68624 | 23.634968 | 10.129272 |  | **New Brunswick /Annual costs** |  |
| New Brunswick | Omeprazole 20mg daily | 2245058 | 90 | 90 | 0.2287 |  | 20.583 | 1.64664 | 11 | 33.22964 | 12.64664 | 23.260748 | 9.968892 |  | Pharmacy Margin | 693.4940726 |
| New Brunswick | Irbesartan/HCTZ 300 mg/25 mg daily | 2447894 | 90 | 90 | 0.2184 |  | 19.656 | 1.57248 | 11 | 32.22848 | 12.57248 | 22.559936 | 9.668544 |  | Government share | 478.26688 |
| New Brunswick | Levothyroxine 50 mcg daily | 2213192 | 90 | 90 |  | 0.0311 | 2.879 | 0 | 11 | 13.879 | 11 | 9.7153 | 4.1637 |  | Patient share | 3882.107593 |
| New Brunswick | Atenolol 50mg daily | 2255545 | 90 | 90 | 0.1107 |  | 9.963 | 0.79704 | 11 | 21.76004 | 11.79704 | 15.232028 | 6.528012 |  |  |  |
| New Brunswick | Liraglutide inj 1.8mg daily | 2351064 | 90 | 27 |  | 29.7367 | 802.8909 | 64.231272 | 11 | 878.122172 | 75.231272 | 0 | 878.122172 |  |  |  |
| New Brunswick | Lorazepam 1mg QHS | 655759 | 90 | 90 | 0.0447 |  | 4.023 | 0.32184 | 11 | 15.34484 | 11.32184 | 10.741388 | 4.603452 |  |  |  |
| New Brunswick | ASAEC 81mg daily | 2237726 | 90 | 90 |  | 0.1095 | 9.855 | 5.306538462 | 0 | 15.16153846 | 5.306538462 | 0 | 15.16153846 |  |  |  |
| New Brunswick | Calcium 500mg / Vitamin D 1000U BID | 80017748 | 90 | 180 |  | 0.09389 | 16.9002 | 9.100107692 | 0 | 26.00030769 | 9.100107692 | 0 | 26.00030769 |  |  |  |
| New Brunswick/ Total Q1 |  |  |  |  |  |  |  |  |  |  | 173.3735182 | 119.56672 | 970.5268982 |  |  |  |
| Newfoundland and Labrador | Metformin 1000mg BID | 2167786 | 90 | 360 |  | 0.0269 | 8.925345622 | 0.758654378 | 12 | 21.684 | 12.75865438 | 15.684 | 6 |  |  |  |
| Newfoundland and Labrador | Atorvastatin 40mg daily | 2295296 | 90 | 90 |  | 0.2553 | 21.17695853 | 1.800041475 | 12 | 34.977 | 13.80004147 | 28.977 | 6 |  | **Newfoundland and Labrador / Annual costs** |  |
| Newfoundland and Labrador | Omeprazole 20mg daily | 2245058 | 90 | 90 |  | 0.2493 | 20.67926267 | 1.757737327 | 12 | 34.437 | 13.75773733 | 28.437 | 6 |  | Pharmacy Margin | 872.3789215 |
| Newfoundland and Labrador | Irbesartan/HCTZ 300 mg/25 mg daily | 2447894 | 90 | 90 |  | 0.2381 | 19.75023041 | 1.678769585 | 12 | 33.429 | 13.67876959 | 27.429 | 6 |  | Government share | 547.26 |
| Newfoundland and Labrador | Levothyroxine 50 mcg daily | 2213192 | 90 | 90 |  | 0.0338 | 2.803686636 | 0.238313364 | 12 | 15.042 | 12.23831336 | 9.042 | 6 |  | Patient share | 3993.251709 |
| Newfoundland and Labrador | Atenolol 50mg daily | 2255545 | 90 | 90 |  | 0.1207 | 10.01198157 | 0.851018433 | 12 | 22.863 | 12.85101843 | 16.863 | 6 |  |  |  |
| Newfoundland and Labrador | Liraglutide inj 1.8mg daily | 2351064 | 90 | 27 |  | 29.7367 | 802.8909 | 72.260181 | 40 | 915.151081 | 112.260181 | 0 | 915.151081 |  |  |  |
| Newfoundland and Labrador | Lorazepam 1mg QHS | 655759 | 90 | 90 |  | 0.0487 | 4.039631336 | 0.343368664 | 12 | 16.383 | 12.34336866 | 10.383 | 6 |  |  |  |
| Newfoundland and Labrador | ASAEC 81mg daily | 2237726 | 90 | 90 |  | 0.1095 | 9.855 | 5.306538462 | 0 | 15.16153846 | 5.306538462 | 0 | 15.16153846 |  |  |  |
| Newfoundland and Labrador | Calcium 500mg / Vitamin D 1000U BID | 80017748 | 90 | 180 |  | 0.09389 | 16.9002 | 9.100107692 | 0 | 26.00030769 | 9.100107692 | 0 | 26.00030769 |  |  |  |
| Newfoundland and Labrador/ Total Q1 |  |  |  |  |  |  |  |  |  |  | 218.0947304 | 136.815 | 998.3129272 |  |  |  |
| Nova Scotia | Metformin 1000mg BID | 2167786 | 90 | 360 | 0.0247 |  | 8.892 | 0.71136 | 11.95 | 21.55336 | 12.66136 | 15.087352 | 6.466008 |  |  |  |
| Nova Scotia | Atorvastatin 40mg daily | 2295296 | 90 | 90 | 0.2342 |  | 21.078 | 1.68624 | 11.95 | 34.71424 | 13.63624 | 24.299968 | 10.414272 |  | **Nova Scotia /Annual costs** |  |
| Nova Scotia | Omeprazole 20mg daily | 2245058 | 90 | 90 | 0.2287 |  | 20.583 | 1.64664 | 11.95 | 34.17964 | 13.59664 | 23.925748 | 10.253892 |  | Pharmacy Margin | 805.3776426 |
| Nova Scotia | Irbesartan/HCTZ 300 mg/25 mg daily | 2447894 | 90 | 90 | 0.2184 |  | 19.656 | 1.57248 | 11.95 | 33.17848 | 13.52248 | 23.224936 | 9.953544 |  | Government share | 497.625016 |
| Nova Scotia | Levothyroxine 50 mcg daily | 2213192 | 90 | 90 |  | 0.0316 | 2.844 | 0.29862 | 11.95 | 15.09262 | 12.24862 | 10.564834 | 4.527786 |  | Patient share | 3974.493027 |
| Nova Scotia | Atenolol 50mg daily | 2255545 | 90 | 90 | 0.1107 |  | 9.963 | 0.79704 | 11.95 | 22.71004 | 12.74704 | 15.897028 | 6.813012 |  |  |  |
| Nova Scotia | Liraglutide inj 1.8mg daily | 2351064 | 90 | 27 |  | 29.7367 | 802.8909 | 84.3035445 | 11.95 | 899.1444445 | 96.2535445 | 0 | 899.1444445 |  |  |  |
| Nova Scotia | Lorazepam 1mg QHS | 655759 | 90 | 90 | 0.0447 |  | 4.023 | 0.32184 | 11.95 | 16.29484 | 12.27184 | 11.406388 | 4.888452 |  |  |  |
| Nova Scotia | ASAEC 81mg daily | 2237726 | 90 | 90 |  | 0.1095 | 9.855 | 5.306538462 | 0 | 15.16153846 | 5.306538462 | 0 | 15.16153846 |  |  |  |
| Nova Scotia | Calcium 500mg / Vitamin D 1000U BID | 80017748 | 90 | 180 |  | 0.09389 | 16.9002 | 9.100107692 | 0 | 26.00030769 | 9.100107692 | 0 | 26.00030769 |  |  |  |
| Nova Scotia/ Total Q1 |  |  |  |  |  |  |  |  |  |  | 201.3444107 | 124.406254 | 993.6232567 |  |  |  |
| Ontario | Metformin 1000mg BID | 2167786 | 90 | 360 |  | 0.0247 | 8.892 | 0.71136 | 8.83 | 18.43336 | 9.54136 | 12.32336 | 6.11 |  |  |  |
| Ontario | Atorvastatin 40mg daily | 2295296 | 90 | 90 |  | 0.2342 | 21.078 | 1.68624 | 8.83 | 31.59424 | 10.51624 | 25.48424 | 6.11 |  | **Ontario /Annual costs** |  |
| Ontario | Omeprazole 20mg daily | 2245058 | 90 | 90 |  | 0.2287 | 20.583 | 1.64664 | 8.83 | 31.05964 | 10.47664 | 24.94964 | 6.11 |  | Pharmacy Margin | 624.9641526 |
| Ontario | Irbesartan/HCTZ 300 mg/25 mg daily | 2447894 | 90 | 90 |  | 0.2184 | 19.656 | 1.57248 | 8.83 | 30.05848 | 10.40248 | 23.94848 | 6.11 |  | Government share | 452.16848 |
| Ontario | Levothyroxine 50 mcg daily | 2213192 | 90 | 90 |  | 0.0316 | 2.844 | 0.22752 | 8.83 | 11.90152 | 9.05752 | 5.79152 | 6.11 |  | Patient share | 3839.536073 |
| Ontario | Atenolol 50mg daily | 2255545 | 90 | 90 |  | 0.1107 | 9.963 | 0.79704 | 8.83 | 19.59004 | 9.62704 | 13.48004 | 6.11 |  |  |  |
| Ontario | Liraglutide inj 1.8mg daily | 2351064 | 90 | 27 |  | 29.7367 | 802.8909 | 64.231272 | 8.83 | 875.952172 | 73.061272 | 0 | 875.952172 |  |  |  |
| Ontario | Lorazepam 1mg QHS | 655759 | 90 | 90 |  | 0.0447 | 4.023 | 0.32184 | 8.83 | 13.17484 | 9.15184 | 7.06484 | 6.11 |  |  |  |
| Ontario | ASAEC 81mg daily | 2237726 | 90 | 90 |  | 0.1095 | 9.855 | 5.306538462 | 0 | 15.16153846 | 5.306538462 | 0 | 15.16153846 |  |  |  |
| Ontario | Calcium 500mg / Vitamin D 1000U BID | 80017748 | 90 | 180 |  | 0.09389 | 16.9002 | 9.100107692 | 0 | 26.00030769 | 9.100107692 | 0 | 26.00030769 |  |  |  |
| Ontario/ Total Q1 |  |  |  |  |  |  |  |  |  |  | 156.2410382 | 113.04212 | 959.8840182 |  |  |  |
| Prince Edward Island | Metformin 1000mg BID | 2167786 | 90 | 360 | 0.0247 |  | 8.892 | 0.53352 | 12.36 | 21.78552 | 12.89352 | 5.84552 | 15.94 |  |  |  |
| Prince Edward Island | Atorvastatin 40mg daily | 2295296 | 90 | 90 | 0.2342 |  | 21.078 | 1.26468 | 12.36 | 34.70268 | 13.62468 | 18.76268 | 15.94 |  | **Prince Edward Island / Annual costs** |  |
| Prince Edward Island | Omeprazole 20mg daily | 2245058 | 90 | 90 | 0.2287 |  | 20.583 | 1.23498 | 12.36 | 34.17798 | 13.59498 | 18.23798 | 15.94 |  | Pharmacy Margin | 666.7168006 |
| Prince Edward Island | Irbesartan/HCTZ 300 mg/25 mg daily | 2447894 | 90 | 90 | 0.2184 |  | 19.656 | 1.17936 | 12.36 | 33.19536 | 13.53936 | 17.25536 | 15.94 |  | Government share | 307.3244 |
| Prince Edward Island | Levothyroxine 50 mcg daily | 2213192 | 90 | 90 |  | 0.031 | 2.79 | 0.1674 | 12.36 | 15.3174 | 12.5274 | 4.8374 | 10.48 |  | Patient share | 4025.916801 |
| Prince Edward Island | Atenolol 50mg daily | 2255545 | 90 | 90 | 0.1107 |  | 9.963 | 0.59778 | 12.36 | 22.92078 | 12.95778 | 6.98078 | 15.94 |  |  |  |
| Prince Edward Island | Liraglutide inj 1.8mg daily | 2351064 | 90 | 27 |  | 29.7367 | 802.8909 | 48.173454 | 12.36 | 863.424354 | 60.533454 | 0 | 863.424354 |  |  |  |
| Prince Edward Island | Lorazepam 1mg QHS | 655759 | 90 | 90 | 0.0447 |  | 4.023 | 0.24138 | 12.36 | 16.62438 | 12.60138 | 4.91138 | 11.713 |  |  |  |
| Prince Edward Island | ASAEC 81mg daily | 2237726 | 90 | 90 |  | 0.1095 | 9.855 | 5.306538462 | 0 | 15.16153846 | 5.306538462 | 0 | 15.16153846 |  |  |  |
| Prince Edward Island | Calcium 500mg / Vitamin D 1000U BID | 80017748 | 90 | 180 |  | 0.09389 | 16.9002 | 9.100107692 | 0 | 26.00030769 | 9.100107692 | 0 | 26.00030769 |  |  |  |
| Prince Edward Island/ Total Q1 |  |  |  |  |  |  |  |  |  |  | 166.6792002 | 76.8311 | 1006.4792 |  |  |  |
| Quebec | Metformin 1000mg BID | 2167786 | 90 | 360 |  | 0.0247 | 8.892 | 0.57798 | 27 | 36.46998 | 27.57798 | 23.74195698 | 12.72802302 |  |  |  |
| Quebec | Atorvastatin 40mg daily | 2295296 | 90 | 90 |  | 0.2342 | 21.078 | 1.37007 | 27 | 49.44807 | 28.37007 | 32.19069357 | 17.25737643 |  | **Quebec /Annual costs** |  |
| Quebec | Omeprazole 20mg daily | 2245058 | 90 | 90 |  | 0.2287 | 20.583 | 1.337895 | 27 | 48.920895 | 28.337895 | 31.84750265 | 17.07339236 |  | Pharmacy Margin | 1104.425045 |
| Quebec | Irbesartan/HCTZ 300 mg/25 mg daily | 2447894 | 90 | 90 |  | 0.2184 | 19.656 | 1.27764 | 27 | 47.93364 | 28.27764 | 31.20479964 | 16.72884036 |  | Government share | 2512.262681 |
| Quebec | Levothyroxine 50 mcg daily | 2213192 | 90 | 90 |  | 0.0274 | 2.466 | 0.16029 | 27 | 29.62629 | 27.16029 | 19.28671479 | 10.33957521 |  | Patient share | 1511.467163 |
| Quebec | Atenolol 50mg daily | 2255545 | 90 | 90 |  | 0.1107 | 9.963 | 0.647595 | 27 | 37.610595 | 27.647595 | 24.48449735 | 13.12609766 |  |  |  |
| Quebec | Liraglutide inj 1.8mg daily | 2351064 | 90 | 27 |  | 22.83 | 616.41 | 40.06665 | 27 | 683.47665 | 67.06665 | 444.9432992 | 238.5333509 |  |  |  |
| Quebec | Lorazepam 1mg QHS | 655759 | 90 | 90 |  | 0.0447 | 4.023 | 0.261495 | 27 | 31.284495 | 27.261495 | 20.36620625 | 10.91828876 |  |  |  |
| Quebec | ASAEC 81mg daily | 2237726 | 90 | 90 |  | 0.1095 | 9.855 | 5.306538462 | 0 | 15.16153846 | 5.306538462 | 0 | 15.16153846 |  |  |  |
| Quebec | Calcium 500mg / Vitamin D 1000U BID | 80017748 | 90 | 180 |  | 0.09389 | 16.9002 | 9.100107692 | 0 | 26.00030769 | 9.100107692 | 0 | 26.00030769 |  |  |  |
| Quebec/Total Q1 |  |  |  |  |  |  |  |  |  |  | 276.1062612 | 628.0656704 | 377.8667908 |  |  |  |
| Saskatchewan | Metformin 1000mg BID | 2167786 | 90 | 360 |  | 0.0247 | 8.892 | 1.3338 | 11.4 | 21.6258 | 12.7338 | 0 | 21.6258 |  |  |  |
| Saskatchewan | Atorvastatin 40mg daily | 2295296 | 90 | 90 |  | 0.2342 | 21.078 | 2.1078 | 11.4 | 34.5858 | 13.5078 | 9.5858 | 25 |  | **Saskatchewan /Annual costs** |  |
| Saskatchewan | Omeprazole 20mg daily | 2245058 | 90 | 90 |  | 0.2287 | 20.583 | 2.0583 | 11.4 | 34.0413 | 13.4583 | 9.0413 | 25 |  | Pharmacy Margin | 546.5067846 |
| Saskatchewan | Irbesartan/HCTZ 300 mg/25 mg daily | 2447894 | 90 | 90 |  | 0.2184 | 19.656 | 1.9656 | 11.4 | 33.0216 | 13.3656 | 8.0216 | 25 |  | Government share | 106.5948 |
| Saskatchewan | Levothyroxine 50 mcg daily | 2213192 | 90 | 90 |  | 0.0316 | 2.844 | 0.8532 | 11.4 | 15.0972 | 12.2532 | 0 | 15.0972 |  | Patient share | 4106.652385 |
| Saskatchewan | Atenolol 50mg daily | 2255545 | 90 | 90 |  | 0.1107 | 9.963 | 1.49445 | 11.4 | 22.85745 | 12.89445 | 0 | 22.85745 |  |  |  |
| Saskatchewan | Liraglutide inj 1.8mg daily | 2351064 | 90 | 27 |  | 29.7367 | 802.8909 | 20 | 11.4 | 834.2909 | 31.4 | 0 | 834.2909 |  |  |  |
| Saskatchewan | Lorazepam 1mg QHS | 655759 | 90 | 90 |  | 0.0447 | 4.023 | 1.2069 | 11.4 | 16.6299 | 12.6069 | 0 | 16.6299 |  |  |  |
| Saskatchewan | ASAEC 81mg daily | 2237726 | 90 | 90 |  | 0.1095 | 9.855 | 5.306538462 | 0 | 15.16153846 | 5.306538462 | 0 | 15.16153846 |  |  |  |
| Saskatchewan | Calcium 500mg / Vitamin D 1000U BID | 80017748 | 90 | 180 |  | 0.09389 | 16.9002 | 9.100107692 | 0 | 26.00030769 | 9.100107692 | 0 | 26.00030769 |  |  |  |
| Saskatchewan/ Total Q1 |  |  |  |  |  |  |  |  |  |  | 136.6266962 | 26.6487 | 1026.663096 |  |  |  |
| Northwest Territories | Metformin 1000mg BID | 2167786 | 90 | 360 |  | 0.0247 | 8.892 | 1.6325712 | 12.72 | 23.2445712 | 14.3525712 | 23.2445712 | 0 |  |  |  |
| Northwest Territories | Atorvastatin 40mg daily | 2295296 | 90 | 90 |  | 0.2342 | 21.078 | 3.8699208 | 12.72 | 37.6679208 | 16.5899208 | 37.6679208 | 0 |  | **Northwest Territories /Annual costs** |  |
| Northwest Territories | Omeprazole 20mg daily | 2245058 | 90 | 90 |  | 0.2287 | 20.583 | 3.7790388 | 12.72 | 37.0820388 | 16.4990388 | 37.0820388 | 0 |  | Pharmacy Margin | 728.5483686 |
| Northwest Territories | Irbesartan/HCTZ 300 mg/25 mg daily | 2447894 | 90 | 90 |  | 0.2184 | 19.656 | 3.6088416 | 12.72 | 35.9848416 | 16.3288416 | 35.9848416 | 0 |  | Government share | 767.981784 |
| Northwest Territories | Levothyroxine 50 mcg daily | 2213192 | 90 | 90 |  | 0.031 | 2.79 | 0.512244 | 12.72 | 16.022244 | 13.232244 | 16.022244 | 0 |  | Patient share | 3627.090985 |
| Northwest Territories | Atenolol 50mg daily | 2255545 | 90 | 90 |  | 0.1107 | 9.963 | 1.8292068 | 12.72 | 24.5122068 | 14.5492068 | 24.5122068 | 0 |  |  |  |
| Northwest Territories | Liraglutide inj 1.8mg daily | 2351064 | 90 | 27 |  | 29.7367 | 802.8909 | 50 | 12.72 | 865.6109 | 62.72 | 0 | 865.6109 |  |  |  |
| Northwest Territories | Lorazepam 1mg QHS | 655759 | 90 | 90 |  | 0.0447 | 4.023 | 0.7386228 | 12.72 | 17.4816228 | 13.4586228 | 17.4816228 | 0 |  |  |  |
| Northwest Territories | ASAEC 81mg daily | 2237726 | 90 | 90 |  | 0.1095 | 9.855 | 5.306538462 | 0 | 15.16153846 | 5.306538462 | 0 | 15.16153846 |  |  |  |
| Northwest Territories | Calcium 500mg / Vitamin D 1000U BID | 80017748 | 90 | 180 |  | 0.09389 | 16.9002 | 9.100107692 | 0 | 26.00030769 | 9.100107692 | 0 | 26.00030769 |  |  |  |
| Northwest Territories |  |  |  |  |  |  |  |  |  |  | 182.1370922 | 191.995446 | 906.7727462 |  |  |  |
| Nunavut | Metformin 1000mg BID | 2167786 | 90 | 360 |  | 0.0247 | 8.892 | 2.1127392 | 16.95 | 27.9547392 | 19.0627392 | 27.9547392 | 0 |  |  |  |
| Nunavut | Atorvastatin 40mg daily | 2295296 | 90 | 90 |  | 0.2342 | 21.078 | 5.0081328 | 16.95 | 43.0361328 | 21.9581328 | 43.0361328 | 0 |  | **Nunavut /Annual costs** |  |
| Nunavut | Omeprazole 20mg daily | 2245058 | 90 | 90 |  | 0.2287 | 20.583 | 4.8905208 | 16.95 | 42.4235208 | 21.8405208 | 42.4235208 | 0 |  | Pharmacy Margin | 882.6971286 |
| Nunavut | Irbesartan/HCTZ 300 mg/25 mg daily | 2447894 | 90 | 90 |  | 0.2184 | 19.656 | 4.6702656 | 16.95 | 41.2762656 | 21.6202656 | 41.2762656 | 0 |  | Government share | 905.210544 |
| Nunavut | Levothyroxine 50 mcg daily | 2213192 | 90 | 90 |  | 0.031 | 2.79 | 0.662904 | 16.95 | 20.402904 | 17.612904 | 20.402904 | 0 |  | Patient share | 3644.010985 |
| Nunavut | Atenolol 50mg daily | 2255545 | 90 | 90 |  | 0.1107 | 9.963 | 2.3672088 | 16.95 | 29.2802088 | 19.3172088 | 29.2802088 | 0 |  |  |  |
| Nunavut | Liraglutide inj 1.8mg daily | 2351064 | 90 | 27 |  | 29.7367 | 802.8909 | 50 | 16.95 | 869.8409 | 66.95 | 0 | 869.8409 |  |  |  |
| Nunavut | Lorazepam 1mg QHS | 655759 | 90 | 90 |  | 0.0447 | 4.023 | 0.9558648 | 16.95 | 21.9288648 | 17.9058648 | 21.9288648 | 0 |  |  |  |
| Nunavut | ASAEC 81mg daily | 2237726 | 90 | 90 |  | 0.1095 | 9.855 | 5.306538462 | 0 | 15.16153846 | 5.306538462 | 0 | 15.16153846 |  |  |  |
| Nunavut | Calcium 500mg / Vitamin D 1000U BID | 80017748 | 90 | 180 |  | 0.09389 | 16.9002 | 9.100107692 | 0 | 26.00030769 | 9.100107692 | 0 | 26.00030769 |  |  |  |
| Nunavut |  |  |  |  |  |  |  |  |  |  | 220.6742822 | 226.302636 | 911.0027462 |  |  |  |
| Yukon | Metformin 1000mg BID | 2167786 | 90 | 360 |  | 0.0247 | 8.892 | 2.1127392 | 12.72 | 23.7247392 | 14.8327392 | 23.7247392 | 0 |  |  |  |
| Yukon | Atorvastatin 40mg daily | 2295296 | 90 | 90 |  | 0.2342 | 21.078 | 5.0081328 | 12.72 | 38.8061328 | 17.7281328 | 38.8061328 | 0 |  | **Yukon /Annual costs** |  |
| Yukon | Omeprazole 20mg daily | 2245058 | 90 | 90 |  | 0.2287 | 20.583 | 4.8905208 | 12.72 | 38.1935208 | 17.6105208 | 38.1935208 | 0 |  | Pharmacy Margin | 783.3462996 |
| Yukon | Irbesartan/HCTZ 300 mg/25 mg daily | 2357410 | 90 | 90 |  | 0.2184 | 19.656 | 4.6702656 | 12.72 | 37.0462656 | 17.3902656 | 37.0462656 | 0 |  | Government share | 870.7538688 |
| Yukon | Levothyroxine 50 mcg daily | 2213192 | 90 | 90 |  | 0.03 | 2.7 | 0.64152 | 12.72 | 16.06152 | 13.36152 | 16.06152 | 0 |  | Patient share | 3566.444831 |
| Yukon | Atenolol 50mg daily | 2255545 | 90 | 90 |  | 0.1107 | 9.963 | 2.3672088 | 12.72 | 25.0502088 | 15.0872088 | 25.0502088 | 0 |  |  |  |
| Yukon | Liraglutide inj 1.8mg daily | 2351064 | 90 | 27 |  | 29.7367 | 802.8909 | 50 | 12.72 | 865.6109 | 62.72 | 0 | 865.6109 |  |  |  |
| Yukon | Lorazepam 1mg QHS | 655759 | 90 | 90 |  | 0.04 | 3.6 | 0.85536 | 12.72 | 17.17536 | 13.57536 | 17.17536 | 0 |  |  |  |
| Yukon | ASAEC 81mg daily | 2237726 | 90 | 90 |  | 0.08 | 7.2 | 1.71072 | 12.72 | 21.63072 | 14.43072 | 21.63072 | 0 |  |  |  |
| Yukon | Calcium 500mg / Vitamin D 1000U BID | 80017748 | 90 | 180 |  | 0.09389 | 16.9002 | 9.100107692 | 0 | 26.00030769 | 9.100107692 | 0 | 26.00030769 |  |  |  |
| Yukon |  |  |  |  |  |  |  |  |  |  | 195.8365749 | 217.6884672 | 891.6112077 |  |  |  |

Supplementary Table 2 – Scenario 1

| **Province** | **Drug** | **DIN** | **Days Supply** | **Quantity** | **MAC/Unit** | **Drug Cost / Unit** | **Drug Cost** | **Markup** | **Dispensing Fee** | **Total Cost** | **Pharmacy Margin $** | **Government Share** | **Patient Share** |  |  |  |
| --- | --- | --- | --- | --- | --- | --- | --- | --- | --- | --- | --- | --- | --- | --- | --- | --- |
| Alberta | Metformin 1000mg BID | 2167786 | 90 | 360 |  | 0.0247 | 8.892 | 0.9078732 | 12.15 | 21.9498732 | 13.0578732 | 15.36491124 | 6.58496196 |  |  |  |
| Alberta | Atorvastatin 40mg daily | 2295296 | 90 | 90 |  | 0.2342 | 21.078 | 2.1520638 | 12.15 | 35.3800638 | 14.3020638 | 24.76604466 | 10.61401914 |  | Alberta /Annual costs |  |
| Alberta | Omeprazole 20mg daily | 2245058 | 90 | 90 | 0 | 0.2287 | 20.583 | 2.1015243 | 12.15 | 34.8345243 | 14.2515243 | 24.38416701 | 10.45035729 |  | Pharmacy Margin | 943.2625357 |
| Alberta | Irbesartan/HCTZ 300 mg/25 mg daily | 2447894 | 90 | 90 |  | 0.2184 | 19.656 | 2.0068776 | 12.15 | 33.8128776 | 14.1568776 | 23.66901432 | 10.14386328 |  | Government share | 506.5652718 |
| Alberta | Levothyroxine 50 mcg daily | 2213192 | 90 | 90 |  | 0.031 | 2.79 | 0.284859 | 12.15 | 15.224859 | 12.434859 | 10.6574013 | 4.5674577 |  | Patient share | 4063.801664 |
| Alberta | Atenolol 50mg daily | 2255545 | 90 | 90 |  | 0.1107 | 9.963 | 1.0172223 | 12.15 | 23.1302223 | 13.1672223 | 16.19115561 | 6.93906669 |  |  |  |
| Alberta | Liraglutide inj 1.8mg daily | 2351064 | 90 | 27 |  | 29.7367 | 802.8909 | 120.6343577 | 12.15 | 935.6752577 | 132.7843577 | 0 | 935.6752577 |  |  |  |
| Alberta | Lorazepam 1mg QHS | 655759 | 90 | 90 |  | 0.0447 | 4.023 | 0.4107483 | 12.15 | 16.5837483 | 12.5607483 | 11.60862381 | 4.97512449 |  |  |  |
| Alberta | Calcium 500mg / Vitamin D 1000U BID | 80017748 | 90 | 180 |  | 0.09389 | 16.9002 | 9.100107692 | 0 | 26.00030769 | 9.100107692 | 0 | 26.00030769 |  |  |  |
| Alberta/ Total Q1 |  |  |  |  |  |  |  |  |  |  | 235.8156339 | 126.641318 | 1015.950416 |  |  |  |
| British Columbia | Metformin 1000mg BID | 2167786 | 90 | 360 | 0.0267 |  | 8.9 | 0.712 | 10 | 19.612 | 10.712 | 0 | 19.612 |  |  |  |
| British Columbia | Atorvastatin 40mg daily | 2295296 | 90 | 90 | 0.2529 |  | 21.075 | 1.686 | 10 | 32.761 | 11.686 | 0 | 32.761 |  | British Columbia /Annual costs |  |
| British Columbia | Omeprazole 20mg daily | 2245058 | 90 | 90 | 0.2025 |  | 16.875 | 1.35 | 10 | 28.225 | 11.35 | 0 | 28.225 |  | Pharmacy Margin | 640.9548521 |
| British Columbia | Irbesartan/HCTZ 300 mg/25 mg daily | 2447894 | 90 | 90 | 0.2719 |  | 22.658333 | 1.812666667 | 10 | 34.471 | 11.81266667 | 0 | 34.471 |  | Government share | 0 |
| British Columbia | Levothyroxine 50 mcg daily | 2213192 | 90 | 90 | 0.0341 |  | 2.8416667 | 0.227333333 | 10 | 13.069 | 10.22733333 | 0 | 13.069 |  | Patient share | 4265.485919 |
| British Columbia | Atenolol 50mg daily | 2255545 | 90 | 90 | 0.1196 |  | 9.9666667 | 0.797333333 | 10 | 20.764 | 10.79733333 | 0 | 20.764 |  |  |  |
| British Columbia | Liraglutide inj 1.8mg daily | 2351064 | 90 | 27 |  | 29.7367 | 802.8909 | 64.231272 | 10 | 877.122172 | 74.231272 | 0 | 877.122172 |  |  |  |
| British Columbia | Lorazepam 1mg QHS | 655759 | 90 | 90 | 0.0483 |  | 4.025 | 0.322 | 10 | 14.347 | 10.322 | 0 | 14.347 |  |  |  |
| British Columbia | Calcium 500mg / Vitamin D 1000U BID | 80017748 | 90 | 180 |  | 0.09389 | 16.9002 | 9.100107692 | 0 | 26.00030769 | 9.100107692 | 0 | 26.00030769 |  |  |  |
| British Columbia/ Total Q1 |  |  |  |  |  |  |  |  |  |  | 160.238713 | 0 | 1066.37148 |  |  |  |
| Manitoba | Metformin 1000mg BID | 2167786 | 90 | 360 |  | 0.0259 | 9.324 | 0 | 13.65 | 22.974 | 13.65 | 22.974 | 0 |  |  |  |
| Manitoba | Atorvastatin 40mg daily | 2295296 | 90 | 90 |  | 0.2459 | 22.131 | 0 | 13.65 | 35.781 | 13.65 | 35.781 | 0 |  | Manitoba /Annual costs |  |
| Manitoba | Omeprazole 20mg daily | 2245058 | 90 | 90 |  | 0.2401 | 21.609 | 0 | 13.65 | 35.259 | 13.65 | 35.259 | 0 |  | Pharmacy Margin | 473.2004308 |
| Manitoba | Irbesartan/HCTZ 300 mg/25 mg daily | 2447894 | 90 | 90 |  | 0.2293 | 20.637 | 0 | 13.65 | 34.287 | 13.65 | 34.287 | 0 |  | Government share | 747.708 |
| Manitoba | Levothyroxine 50 mcg daily | 2213192 | 90 | 90 |  | 0.031 | 2.79 | 0 | 13.65 | 16.44 | 13.65 | 16.44 | 0 |  | Patient share | 3370.164831 |
| Manitoba | Atenolol 50mg daily | 2255545 | 90 | 90 |  | 0.1162 | 10.458 | 0 | 13.65 | 24.108 | 13.65 | 24.108 | 0 |  |  |  |
| Manitoba | Liraglutide inj 1.8mg daily | 2351064 | 90 | 27 |  | 29.7367 | 802.8909 | 0 | 13.65 | 816.5409 | 13.65 | 0 | 816.5409 |  |  |  |
| Manitoba | Lorazepam 1mg QHS | 655759 | 90 | 90 |  | 0.0492 | 4.428 | 0 | 13.65 | 18.078 | 13.65 | 18.078 | 0 |  |  |  |
| Manitoba | Calcium 500mg / Vitamin D 1000U BID | 80017748 | 90 | 180 |  | 0.09389 | 16.9002 | 9.100107692 | 0 | 26.00030769 | 9.100107692 | 0 | 26.00030769 |  |  |  |
| Manitoba/ Total Q1 |  |  |  |  |  |  |  |  |  |  | 118.3001077 | 186.927 | 842.5412077 |  |  |  |
| New Brunswick | Metformin 1000mg BID | 2167786 | 90 | 360 | 0.0247 |  | 8.892 | 0.71136 | 11 | 20.60336 | 11.71136 | 14.422352 | 6.181008 |  |  |  |
| New Brunswick | Atorvastatin 40mg daily | 2295296 | 90 | 90 | 0.2342 |  | 21.078 | 1.68624 | 11 | 33.76424 | 12.68624 | 23.634968 | 10.129272 |  | New Brunswick /Annual costs |  |
| New Brunswick | Omeprazole 20mg daily | 2245058 | 90 | 90 | 0.2287 |  | 20.583 | 1.64664 | 11 | 33.22964 | 12.64664 | 23.260748 | 9.968892 |  | Pharmacy Margin | 672.2679188 |
| New Brunswick | Irbesartan/HCTZ 300 mg/25 mg daily | 2447894 | 90 | 90 | 0.2184 |  | 19.656 | 1.57248 | 11 | 32.22848 | 12.57248 | 22.559936 | 9.668544 |  | Government share | 478.26688 |
| New Brunswick | Levothyroxine 50 mcg daily | 2213192 | 90 | 90 |  | 0.0311 | 2.879 | 0 | 11 | 13.879 | 11 | 9.7153 | 4.1637 |  | Patient share | 3821.461439 |
| New Brunswick | Atenolol 50mg daily | 2255545 | 90 | 90 | 0.1107 |  | 9.963 | 0.79704 | 11 | 21.76004 | 11.79704 | 15.232028 | 6.528012 |  |  |  |
| New Brunswick | Liraglutide inj 1.8mg daily | 2351064 | 90 | 27 |  | 29.7367 | 802.8909 | 64.231272 | 11 | 878.122172 | 75.231272 | 0 | 878.122172 |  |  |  |
| New Brunswick | Lorazepam 1mg QHS | 655759 | 90 | 90 | 0.0447 |  | 4.023 | 0.32184 | 11 | 15.34484 | 11.32184 | 10.741388 | 4.603452 |  |  |  |
| New Brunswick | Calcium 500mg / Vitamin D 1000U BID | 80017748 | 90 | 180 |  | 0.09389 | 16.9002 | 9.100107692 | 0 | 26.00030769 | 9.100107692 | 0 | 26.00030769 |  |  |  |
| New Brunswick/ Total Q1 |  |  |  |  |  |  |  |  |  |  | 168.0669797 | 119.56672 | 955.3653597 |  |  |  |
| Newfoundland and Labrador | Metformin 1000mg BID | 2167786 | 90 | 360 |  | 0.0269 | 8.9253456 | 0.758654378 | 12 | 21.684 | 12.75865438 | 15.684 | 6 |  |  |  |
| Newfoundland and Labrador | Atorvastatin 40mg daily | 2295296 | 90 | 90 |  | 0.2553 | 21.176959 | 1.800041475 | 12 | 34.977 | 13.80004147 | 28.977 | 6 |  | Newfoundland and Labrador / Annual costs |  |
| Newfoundland and Labrador | Omeprazole 20mg daily | 2245058 | 90 | 90 |  | 0.2493 | 20.679263 | 1.757737327 | 12 | 34.437 | 13.75773733 | 28.437 | 6 |  | Pharmacy Margin | 851.1527677 |
| Newfoundland and Labrador | Irbesartan/HCTZ 300 mg/25 mg daily | 2447894 | 90 | 90 |  | 0.2381 | 19.75023 | 1.678769585 | 12 | 33.429 | 13.67876959 | 27.429 | 6 |  | Government share | 547.26 |
| Newfoundland and Labrador | Levothyroxine 50 mcg daily | 2213192 | 90 | 90 |  | 0.0338 | 2.8036866 | 0.238313364 | 12 | 15.042 | 12.23831336 | 9.042 | 6 |  | Patient share | 3932.605555 |
| Newfoundland and Labrador | Atenolol 50mg daily | 2255545 | 90 | 90 |  | 0.1207 | 10.011982 | 0.851018433 | 12 | 22.863 | 12.85101843 | 16.863 | 6 |  |  |  |
| Newfoundland and Labrador | Liraglutide inj 1.8mg daily | 2351064 | 90 | 27 |  | 29.7367 | 802.8909 | 72.260181 | 40 | 915.151081 | 112.260181 | 0 | 915.151081 |  |  |  |
| Newfoundland and Labrador | Lorazepam 1mg QHS | 655759 | 90 | 90 |  | 0.0487 | 4.0396313 | 0.343368664 | 12 | 16.383 | 12.34336866 | 10.383 | 6 |  |  |  |
| Newfoundland and Labrador | Calcium 500mg / Vitamin D 1000U BID | 80017748 | 90 | 180 |  | 0.09389 | 16.9002 | 9.100107692 | 0 | 26.00030769 | 9.100107692 | 0 | 26.00030769 |  |  |  |
| Newfoundland and Labrador/ Total Q1 |  |  |  |  |  |  |  |  |  |  | 212.7881919 | 136.815 | 983.1513887 |  |  |  |
| Nova Scotia | Metformin 1000mg BID | 2167786 | 90 | 360 | 0.0247 |  | 8.892 | 0.71136 | 11.95 | 21.55336 | 12.66136 | 15.087352 | 6.466008 |  |  |  |
| Nova Scotia | Atorvastatin 40mg daily | 2295296 | 90 | 90 | 0.2342 |  | 21.078 | 1.68624 | 11.95 | 34.71424 | 13.63624 | 24.299968 | 10.414272 |  | Nova Scotia /Annual costs |  |
| Nova Scotia | Omeprazole 20mg daily | 2245058 | 90 | 90 | 0.2287 |  | 20.583 | 1.64664 | 11.95 | 34.17964 | 13.59664 | 23.925748 | 10.253892 |  | Pharmacy Margin | 784.1514888 |
| Nova Scotia | Irbesartan/HCTZ 300 mg/25 mg daily | 2447894 | 90 | 90 | 0.2184 |  | 19.656 | 1.57248 | 11.95 | 33.17848 | 13.52248 | 23.224936 | 9.953544 |  | Government share | 497.625016 |
| Nova Scotia | Levothyroxine 50 mcg daily | 2213192 | 90 | 90 |  |  | 2.844 | 0.29862 | 11.95 | 15.09262 | 12.24862 | 10.564834 | 4.527786 |  | Patient share | 3913.846873 |
| Nova Scotia | Atenolol 50mg daily | 2255545 | 90 | 90 | 0.1107 |  | 9.963 | 0.79704 | 11.95 | 22.71004 | 12.74704 | 15.897028 | 6.813012 |  |  |  |
| Nova Scotia | Liraglutide inj 1.8mg daily | 2351064 | 90 | 27 |  | 29.7367 | 802.8909 | 84.3035445 | 11.95 | 899.1444445 | 96.2535445 | 0 | 899.1444445 |  |  |  |
| Nova Scotia | Lorazepam 1mg QHS | 655759 | 90 | 90 | 0.0447 |  | 4.023 | 0.32184 | 11.95 | 16.29484 | 12.27184 | 11.406388 | 4.888452 |  |  |  |
| Nova Scotia | Calcium 500mg / Vitamin D 1000U BID | 80017748 | 90 | 180 |  | 0.09389 | 16.9002 | 9.100107692 | 0 | 26.00030769 | 9.100107692 | 0 | 26.00030769 |  |  |  |
| Nova Scotia/ Total Q1 |  |  |  |  |  |  |  |  |  |  | 196.0378722 | 124.406254 | 978.4617182 |  |  |  |
| Ontario | Metformin 1000mg BID | 2167786 | 90 | 360 |  | 0.0247 | 8.892 | 0.71136 | 8.83 | 18.43336 | 9.54136 | 12.32336 | 6.11 |  |  |  |
| Ontario | Atorvastatin 40mg daily | 2295296 | 90 | 90 |  | 0.2342 | 21.078 | 1.68624 | 8.83 | 31.59424 | 10.51624 | 25.48424 | 6.11 |  | Ontario /Annual costs |  |
| Ontario | Omeprazole 20mg daily | 2245058 | 90 | 90 |  | 0.2287 | 20.583 | 1.64664 | 8.83 | 31.05964 | 10.47664 | 24.94964 | 6.11 |  | Pharmacy Margin | 603.7379988 |
| Ontario | Irbesartan/HCTZ 300 mg/25 mg daily | 2447894 | 90 | 90 |  | 0.2184 | 19.656 | 1.57248 | 8.83 | 30.05848 | 10.40248 | 23.94848 | 6.11 |  | Government share | 452.16848 |
| Ontario | Levothyroxine 50 mcg daily | 2213192 | 90 | 90 |  | 0.0316 | 2.844 | 0.22752 | 8.83 | 11.90152 | 9.05752 | 5.79152 | 6.11 |  | Patient share | 3778.889919 |
| Ontario | Atenolol 50mg daily | 2255545 | 90 | 90 |  | 0.1107 | 9.963 | 0.79704 | 8.83 | 19.59004 | 9.62704 | 13.48004 | 6.11 |  |  |  |
| Ontario | Liraglutide inj 1.8mg daily | 2351064 | 90 | 27 |  | 29.7367 | 802.8909 | 64.231272 | 8.83 | 875.952172 | 73.061272 | 0 | 875.952172 |  |  |  |
| Ontario | Lorazepam 1mg QHS | 655759 | 90 | 90 |  | 0.0447 | 4.023 | 0.32184 | 8.83 | 13.17484 | 9.15184 | 7.06484 | 6.11 |  |  |  |
| Ontario | Calcium 500mg / Vitamin D 1000U BID | 80017748 | 90 | 180 |  | 0.09389 | 16.9002 | 9.100107692 | 0 | 26.00030769 | 9.100107692 | 0 | 26.00030769 |  |  |  |
| Ontario/ Total Q1 |  |  |  |  |  |  |  |  |  |  | 150.9344997 | 113.04212 | 944.7224797 |  |  |  |
| Prince Edward Island | Metformin 1000mg BID | 2167786 | 90 | 360 | 0.0247 |  | 8.892 | 0.53352 | 12.36 | 21.78552 | 12.89352 | 5.84552 | 15.94 |  |  |  |
| Prince Edward Island | Atorvastatin 40mg daily | 2295296 | 90 | 90 | 0.2342 |  | 21.078 | 1.26468 | 12.36 | 34.70268 | 13.62468 | 18.76268 | 15.94 |  | Prince Edward Island / Annual costs |  |
| Prince Edward Island | Omeprazole 20mg daily | 2245058 | 90 | 90 | 0.2287 |  | 20.583 | 1.23498 | 12.36 | 34.17798 | 13.59498 | 18.23798 | 15.94 |  | Pharmacy Margin | 645.4906468 |
| Prince Edward Island | Irbesartan/HCTZ 300 mg/25 mg daily | 2447894 | 90 | 90 | 0.2184 |  | 19.656 | 1.17936 | 12.36 | 33.19536 | 13.53936 | 17.25536 | 15.94 |  | Government share | 307.3244 |
| Prince Edward Island | Levothyroxine 50 mcg daily | 2213192 | 90 | 90 |  | 0.031 | 2.79 | 0.1674 | 12.36 | 15.3174 | 12.5274 | 4.8374 | 10.48 |  | Patient share | 3965.270647 |
| Prince Edward Island | Atenolol 50mg daily | 2255545 | 90 | 90 | 0.1107 |  | 9.963 | 0.59778 | 12.36 | 22.92078 | 12.95778 | 6.98078 | 15.94 |  |  |  |
| Prince Edward Island | Liraglutide inj 1.8mg daily | 2351064 | 90 | 27 |  | 29.7367 | 802.8909 | 48.173454 | 12.36 | 863.424354 | 60.533454 | 0 | 863.424354 |  |  |  |
| Prince Edward Island | Lorazepam 1mg QHS | 655759 | 90 | 90 | 0.0447 |  | 4.023 | 0.24138 | 12.36 | 16.62438 | 12.60138 | 4.91138 | 11.713 |  |  |  |
| Prince Edward Island | Calcium 500mg / Vitamin D 1000U BID | 80017748 | 90 | 180 |  | 0.09389 | 16.9002 | 9.100107692 | 0 | 26.00030769 | 9.100107692 | 0 | 26.00030769 |  |  |  |
| Prince Edward Island/ Total Q1 |  |  |  |  |  |  |  |  |  |  | 161.3726617 | 76.8311 | 991.3176617 |  |  |  |
| Quebec | Metformin 1000mg BID | 2167786 | 90 | 360 |  | 0.0247 | 8.892 | 0.57798 | 27 | 36.46998 | 27.57798 | 23.74195698 | 12.72802302 |  |  |  |
| Quebec | Atorvastatin 40mg daily | 2295296 | 90 | 90 |  | 0.2342 | 21.078 | 1.37007 | 27 | 49.44807 | 28.37007 | 32.19069357 | 17.25737643 |  | Quebec /Annual costs |  |
| Quebec | Omeprazole 20mg daily | 2245058 | 90 | 90 |  | 0.2287 | 20.583 | 1.337895 | 27 | 48.920895 | 28.337895 | 31.84750265 | 17.07339236 |  | Pharmacy Margin | 1083.198891 |
| Quebec | Irbesartan/HCTZ 300 mg/25 mg daily | 2447894 | 90 | 90 |  | 0.2184 | 19.656 | 1.27764 | 27 | 47.93364 | 28.27764 | 31.20479964 | 16.72884036 |  | Government share | 2512.262681 |
| Quebec | Levothyroxine 50 mcg daily | 2213192 | 90 | 90 |  | 0.0274 | 2.466 | 0.16029 | 27 | 29.62629 | 27.16029 | 19.28671479 | 10.33957521 |  | Patient share | 1450.821009 |
| Quebec | Atenolol 50mg daily | 2255545 | 90 | 90 |  | 0.1107 | 9.963 | 0.647595 | 27 | 37.610595 | 27.647595 | 24.48449735 | 13.12609766 |  |  |  |
| Quebec | Liraglutide inj 1.8mg daily | 2351064 | 90 | 27 |  | 22.83 | 616.41 | 40.06665 | 27 | 683.47665 | 67.06665 | 444.9432992 | 238.5333509 |  |  |  |
| Quebec | Lorazepam 1mg QHS | 655759 | 90 | 90 |  | 0.0447 | 4.023 | 0.261495 | 27 | 31.284495 | 27.261495 | 20.36620625 | 10.91828876 |  |  |  |
| Quebec | Calcium 500mg / Vitamin D 1000U BID | 80017748 | 90 | 180 |  | 0.09389 | 16.9002 | 9.100107692 | 0 | 26.00030769 | 9.100107692 | 0 | 26.00030769 |  |  |  |
| Quebec/Total Q1 |  |  |  |  |  |  |  |  |  |  | 270.7997227 | 628.0656704 | 362.7052523 |  |  |  |
| Saskatchewan | Metformin 1000mg BID | 2167786 | 90 | 360 |  | 0.0247 | 8.892 | 1.3338 | 11.4 | 21.6258 | 12.7338 | 0 | 21.6258 |  |  |  |
| Saskatchewan | Atorvastatin 40mg daily | 2295296 | 90 | 90 |  | 0.2342 | 21.078 | 2.1078 | 11.4 | 34.5858 | 13.5078 | 9.5858 | 25 |  | Saskatchewan /Annual costs |  |
| Saskatchewan | Omeprazole 20mg daily | 2245058 | 90 | 90 |  | 0.2287 | 20.583 | 2.0583 | 11.4 | 34.0413 | 13.4583 | 9.0413 | 25 |  | Pharmacy Margin | 525.2806308 |
| Saskatchewan | Irbesartan/HCTZ 300 mg/25 mg daily | 2447894 | 90 | 90 |  | 0.2184 | 19.656 | 1.9656 | 11.4 | 33.0216 | 13.3656 | 8.0216 | 25 |  | Government share | 106.5948 |
| Saskatchewan | Levothyroxine 50 mcg daily | 2213192 | 90 | 90 |  | 0.0316 | 2.844 | 0.8532 | 11.4 | 15.0972 | 12.2532 | 0 | 15.0972 |  | Patient share | 4046.006231 |
| Saskatchewan | Atenolol 50mg daily | 2255545 | 90 | 90 |  | 0.1107 | 9.963 | 1.49445 | 11.4 | 22.85745 | 12.89445 | 0 | 22.85745 |  |  |  |
| Saskatchewan | Liraglutide inj 1.8mg daily | 2351064 | 90 | 27 |  | 29.7367 | 802.8909 | 20 | 11.4 | 834.2909 | 31.4 | 0 | 834.2909 |  |  |  |
| Saskatchewan | Lorazepam 1mg QHS | 655759 | 90 | 90 |  | 0.0447 | 4.023 | 1.2069 | 11.4 | 16.6299 | 12.6069 | 0 | 16.6299 |  |  |  |
| Saskatchewan | Calcium 500mg / Vitamin D 1000U BID | 80017748 | 90 | 180 |  | 0.09389 | 16.9002 | 9.100107692 | 0 | 26.00030769 | 9.100107692 | 0 | 26.00030769 |  |  |  |
| Saskatchewan/ Total Q1 |  |  |  |  |  |  |  |  |  |  | 131.3201577 | 26.6487 | 1011.501558 |  |  |  |
| Northwest Territories | Metformin 1000mg BID | 2167786 | 90 | 360 | 0 | 0.0247 | 8.892 | 1.6325712 | 12.72 | 23.2445712 | 14.3525712 | 23.2445712 | 0 |  |  |  |
| Northwest Territories | Atorvastatin 40mg daily | 2295296 | 90 | 90 | 0 | 0.2342 | 21.078 | 3.8699208 | 12.72 | 37.6679208 | 16.5899208 | 37.6679208 | 0 |  | Northwest Territories /Annual costs |  |
| Northwest Territories | Omeprazole 20mg daily | 2245058 | 90 | 90 | 0 | 0.2287 | 20.583 | 3.7790388 | 12.72 | 37.0820388 | 16.4990388 | 37.0820388 | 0 |  | Pharmacy Margin | 707.3222148 |
| Northwest Territories | Irbesartan/HCTZ 300 mg/25 mg daily | 2447894 | 90 | 90 | 0 | 0.2184 | 19.656 | 3.6088416 | 12.72 | 35.9848416 | 16.3288416 | 35.9848416 | 0 |  | Government share | 767.981784 |
| Northwest Territories | Levothyroxine 50 mcg daily | 2213192 | 90 | 90 | 0 | 0.031 | 2.79 | 0.512244 | 12.72 | 16.022244 | 13.232244 | 16.022244 | 0 |  | Patient share | 3566.444831 |
| Northwest Territories | Atenolol 50mg daily | 2255545 | 90 | 90 | 0 | 0.1107 | 9.963 | 1.8292068 | 12.72 | 24.5122068 | 14.5492068 | 24.5122068 | 0 |  |  |  |
| Northwest Territories | Liraglutide inj 1.8mg daily | 2351064 | 90 | 27 | 0 | 29.7367 | 802.8909 | 50 | 12.72 | 865.6109 | 62.72 | 0 | 865.6109 |  |  |  |
| Northwest Territories | Lorazepam 1mg QHS | 655759 | 90 | 90 | 0 | 0.0447 | 4.023 | 0.7386228 | 12.72 | 17.4816228 | 13.4586228 | 17.4816228 | 0 |  |  |  |
| Northwest Territories | Calcium 500mg / Vitamin D 1000U BID | 80017748 | 90 | 180 | 0 | 0.09389 | 16.9002 | 9.100107692 | 0 | 26.00030769 | 9.100107692 | 0 | 26.00030769 |  |  |  |
| Northwest Territories |  |  |  |  |  |  |  |  |  |  | 176.8305537 | 191.995446 | 891.6112077 |  |  |  |
| Nunavut | Metformin 1000mg BID | 2167786 | 90 | 360 | 0 | 0.0247 | 8.892 | 2.1127392 | 16.95 | 27.9547392 | 19.0627392 | 27.9547392 | 0 |  |  |  |
| Nunavut | Atorvastatin 40mg daily | 2295296 | 90 | 90 | 0 | 0.2342 | 21.078 | 5.0081328 | 16.95 | 43.0361328 | 21.9581328 | 43.0361328 | 0 |  | Nunavut /Annual costs |  |
| Nunavut | Omeprazole 20mg daily | 2245058 | 90 | 90 | 0 | 0.2287 | 20.583 | 4.8905208 | 16.95 | 42.4235208 | 21.8405208 | 42.4235208 | 0 |  | Pharmacy Margin | 861.4709748 |
| Nunavut | Irbesartan/HCTZ 300 mg/25 mg daily | 2447894 | 90 | 90 | 0 | 0.2184 | 19.656 | 4.6702656 | 16.95 | 41.2762656 | 21.6202656 | 41.2762656 | 0 |  | Government share | 905.210544 |
| Nunavut | Levothyroxine 50 mcg daily | 2213192 | 90 | 90 | 0 | 0.031 | 2.79 | 0.662904 | 16.95 | 20.402904 | 17.612904 | 20.402904 | 0 |  | Patient share | 3583.364831 |
| Nunavut | Atenolol 50mg daily | 2255545 | 90 | 90 | 0 | 0.1107 | 9.963 | 2.3672088 | 16.95 | 29.2802088 | 19.3172088 | 29.2802088 | 0 |  |  |  |
| Nunavut | Liraglutide inj 1.8mg daily | 2351064 | 90 | 27 | 0 | 29.7367 | 802.8909 | 50 | 16.95 | 869.8409 | 66.95 | 0 | 869.8409 |  |  |  |
| Nunavut | Lorazepam 1mg QHS | 655759 | 90 | 90 | 0 | 0.0447 | 4.023 | 0.9558648 | 16.95 | 21.9288648 | 17.9058648 | 21.9288648 | 0 |  |  |  |
| Nunavut | Calcium 500mg / Vitamin D 1000U BID | 80017748 | 90 | 180 | 0 | 0.09389 | 16.9002 | 9.100107692 | 0 | 26.00030769 | 9.100107692 | 0 | 26.00030769 |  |  |  |
| Nunavut |  |  |  |  |  |  |  |  |  |  | 215.3677437 | 226.302636 | 895.8412077 |  |  |  |
| Yukon | Metformin 1000mg BID | 2167786 | 90 | 360 | 0 | 0.0247 | 8.892 | 2.1127392 | 12.72 | 23.7247392 | 14.8327392 | 23.7247392 | 0 |  |  |  |
| Yukon | Atorvastatin 40mg daily | 2295296 | 90 | 90 | 0 | 0.2342 | 21.078 | 5.0081328 | 12.72 | 38.8061328 | 17.7281328 | 38.8061328 | 0 |  | Yukon /Annual costs |  |
| Yukon | Omeprazole 20mg daily | 2245058 | 90 | 90 | 0 | 0.2287 | 20.583 | 4.8905208 | 12.72 | 38.1935208 | 17.6105208 | 38.1935208 | 0 |  | Pharmacy Margin | 725.6234196 |
| Yukon | Irbesartan/HCTZ 300 mg/25 mg daily | 2357410 | 90 | 90 | 0 | 0.2184 | 19.656 | 4.6702656 | 12.72 | 37.0462656 | 17.3902656 | 37.0462656 | 0 |  | Government share | 784.2309888 |
| Yukon | Levothyroxine 50 mcg daily | 2213192 | 90 | 90 | 0 | 0.03 | 2.7 | 0.64152 | 12.72 | 16.06152 | 13.36152 | 16.06152 | 0 |  | Patient share | 3566.444831 |
| Yukon | Atenolol 50mg daily | 2255545 | 90 | 90 | 0 | 0.1107 | 9.963 | 2.3672088 | 12.72 | 25.0502088 | 15.0872088 | 25.0502088 | 0 |  |  |  |
| Yukon | Liraglutide inj 1.8mg daily | 2351064 | 90 | 27 | 0 | 29.7367 | 802.8909 | 50 | 12.72 | 865.6109 | 62.72 | 0 | 865.6109 |  |  |  |
| Yukon | Lorazepam 1mg QHS | 655759 | 90 | 90 | 0 | 0.04 | 3.6 | 0.85536 | 12.72 | 17.17536 | 13.57536 | 17.17536 | 0 |  |  |  |
| Yukon | Calcium 500mg / Vitamin D 1000U BID | 80017748 | 90 | 180 | 0 | 0.09389 | 16.9002 | 9.100107692 | 0 | 26.00030769 | 9.100107692 | 0 | 26.00030769 |  |  |  |
| Yukon |  |  |  |  |  |  |  |  |  |  | 181.4058549 | 196.0577472 | 891.6112077 |  |  |  |
|  |  |  |  |  |  |  |  |  |  |  |  |  |  |  |  |  |
|  |  |  |  |  |  |  |  |  |  |  |  |  |  |  |  |  |
|  |  |  |  |  |  |  |  |  |  |  |  |  |  |  |  |  |
|  |  |  |  |  |  |  |  |  |  |  |  |  |  |  |  |  |
|  |  |  |  |  |  |  |  |  |  |  |  |  |  |  |  |  |

Supplementary Table 3 – Scenario 2

| **Province** | **Drug** | **DIN** | **Days Supply** | **Quantity** | **MAC/Unit** | **Drug Cost / Unit** | **Drug Cost** | **Markup** | **Dispensing Fee** | **Total Cost** | **Pharmacy Margin $** | **Government Share** | **Patient Share** |  |  |  |
| --- | --- | --- | --- | --- | --- | --- | --- | --- | --- | --- | --- | --- | --- | --- | --- | --- |
| Alberta | Metformin 1000mg BID | 2167786 | 90 | 360 |  | 0.0247 | 8.892 | 0.9078732 | 12.15 | 21.9498732 | 13.0578732 | 15.36491124 | 6.58496196 |  | Alberta /Annual costs |  |
| Alberta | Omeprazole 20mg daily | 2245058 | 90 | 90 |  | 0.2287 | 20.583 | 2.1015243 | 12.15 | 34.8345243 | 14.2515243 | 24.38416701 | 10.45035729 |  | Pharmacy Margin | 907.2804343 |
| Alberta | Irbesartan/HCTZ 300 mg/25 mg daily | 2447894 | 90 | 90 |  | 0.2184 | 19.656 | 2.0068776 | 12.15 | 33.8128776 | 14.1568776 | 23.66901432 | 10.14386328 |  | Government share | 407.5010932 |
| Alberta | Levothyroxine 50 mcg daily | 2213192 | 90 | 90 |  | 0.031 | 2.79 | 0.284859 | 12.15 | 15.224859 | 12.434859 | 10.6574013 | 4.5674577 |  | Patient share | 4081.991741 |
| Alberta | Atenolol 50mg daily | 2255545 | 90 | 90 |  | 0.1107 | 9.963 | 1.0172223 | 12.15 | 23.1302223 | 13.1672223 | 16.19115561 | 6.93906669 |  |  |  |
| Alberta | Liraglutide inj 1.8mg daily | 2351064 | 90 | 27 |  | 29.7367 | 802.8909 | 120.6343577 | 12.15 | 935.6752577 | 132.7843577 | 0 | 935.6752577 |  |  |  |
| Alberta | Lorazepam 1mg QHS | 655759 | 90 | 90 |  | 0.0447 | 4.023 | 0.4107483 | 12.15 | 16.5837483 | 12.5607483 | 11.60862381 | 4.97512449 |  |  |  |
| Alberta | ASAEC 81mg daily | 2237726 | 90 | 90 |  | 0.1095 | 9.855 | 5.306538462 | 0 | 15.16153846 | 5.306538462 | 0 | 15.16153846 |  |  |  |
| Alberta | Calcium 500mg / Vitamin D 1000U BID | 80017748 | 90 | 180 |  | 0.09389 | 16.9002 | 9.100107692 | 0 | 26.00030769 | 9.100107692 | 0 | 26.00030769 |  |  |  |
| Alberta/ Total Q1 |  |  |  |  |  |  |  |  |  |  | 226.8201086 | 101.8752733 | 1020.497935 |  |  |  |
| British Columbia | Metformin 1000mg BID | 2167786 | 90 | 360 | 0.0267 |  | 8.9 | 0.712 | 10 | 19.612 | 10.712 | 0 | 19.612 |  | British Columbia /Annual costs |  |
| British Columbia | Omeprazole 20mg daily | 2245058 | 90 | 90 | 0.2025 |  | 16.875 | 1.35 | 10 | 28.225 | 11.35 | 0 | 28.225 |  | Pharmacy Margin | 636.9708521 |
| British Columbia | Irbesartan/HCTZ 300 mg/25 mg daily | 2447894 | 90 | 90 | 0.2719 |  | 22.658333 | 1.812666667 | 10 | 34.471 | 11.81266667 | 0 | 34.471 |  | Government share | 0 |
| British Columbia | Levothyroxine 50 mcg daily | 2213192 | 90 | 90 | 0.0341 |  | 2.8416667 | 0.227333333 | 10 | 13.069 | 10.22733333 | 0 | 13.069 |  | Patient share | 4211.701919 |
| British Columbia | Atenolol 50mg daily | 2255545 | 90 | 90 | 0.1196 |  | 9.9666667 | 0.797333333 | 10 | 20.764 | 10.79733333 | 0 | 20.764 |  |  |  |
| British Columbia | Liraglutide inj 1.8mg daily | 2351064 | 90 | 27 |  | 29.7367 | 802.8909 | 64.231272 | 10 | 877.122172 | 74.231272 | 0 | 877.122172 |  |  |  |
| British Columbia | Lorazepam 1mg QHS | 655759 | 90 | 90 | 0.0483 |  | 4.025 | 0.322 | 10 | 14.347 | 10.322 | 0 | 14.347 |  |  |  |
| British Columbia | ASAEC 81mg daily | 2237726 | 90 | 90 | 0.1035 |  | 8.625 | 0.69 | 10 | 19.315 | 10.69 | 0 | 19.315 |  |  |  |
| British Columbia | Calcium 500mg / Vitamin D 1000U BID | 80017748 | 90 | 180 |  | 0.09389 | 16.9002 | 9.100107692 | 0 | 26.00030769 | 9.100107692 | 0 | 26.00030769 |  |  |  |
| British Columbia/ Total Q1 |  |  |  |  |  |  |  |  |  |  | 159.242713 | 0 | 1052.92548 |  |  |  |
| Manitoba | Metformin 1000mg BID | 2167786 | 90 | 360 |  | 0.0259 | 9.324 | 0 | 13.65 | 22.974 | 13.65 | 22.974 | 0 |  | Manitoba /Annual costs |  |
| Manitoba | Omeprazole 20mg daily | 2245058 | 90 | 90 |  | 0.2401 | 21.609 | 0 | 13.65 | 35.259 | 13.65 | 35.259 | 0 |  | Pharmacy Margin | 439.8265846 |
| Manitoba | Irbesartan/HCTZ 300 mg/25 mg daily | 2447894 | 90 | 90 |  | 0.2293 | 20.637 | 0 | 13.65 | 34.287 | 13.65 | 34.287 | 0 |  | Government share | 604.584 |
| Manitoba | Levothyroxine 50 mcg daily | 2213192 | 90 | 90 |  | 0.031 | 2.79 | 0 | 13.65 | 16.44 | 13.65 | 16.44 | 0 |  | Patient share | 3430.810985 |
| Manitoba | Atenolol 50mg daily | 2255545 | 90 | 90 |  | 0.1162 | 10.458 | 0 | 13.65 | 24.108 | 13.65 | 24.108 | 0 |  |  |  |
| Manitoba | Liraglutide inj 1.8mg daily | 2351064 | 90 | 27 |  | 29.7367 | 802.8909 | 0 | 13.65 | 816.5409 | 13.65 | 0 | 816.5409 |  |  |  |
| Manitoba | Lorazepam 1mg QHS | 655759 | 90 | 90 |  | 0.0492 | 4.428 | 0 | 13.65 | 18.078 | 13.65 | 18.078 | 0 |  |  |  |
| Manitoba | ASAEC 81mg daily | 2237726 | 90 | 90 |  | 0.1095 | 9.855 | 5.306538462 | 0 | 15.16153846 | 5.306538462 | 0 | 15.16153846 |  |  |  |
| Manitoba | Calcium 500mg / Vitamin D 1000U BID | 80017748 | 90 | 180 |  | 0.09389 | 16.9002 | 9.100107692 | 0 | 26.00030769 | 9.100107692 | 0 | 26.00030769 |  |  |  |
| Manitoba/ Total Q1 |  |  |  |  |  |  |  |  |  |  | 109.9566462 | 151.146 | 857.7027462 |  |  |  |
| New Brunswick | Metformin 1000mg BID | 2167786 | 90 | 360 | 0.0247 |  | 8.892 | 0.71136 | 11 | 20.60336 | 11.71136 | 14.422352 | 6.181008 |  | New Brunswick /Annual costs |  |
| New Brunswick | Omeprazole 20mg daily | 2245058 | 90 | 90 | 0.2287 |  | 20.583 | 1.64664 | 11 | 33.22964 | 12.64664 | 23.260748 | 9.968892 |  | Pharmacy Margin | 642.7491126 |
| New Brunswick | Irbesartan/HCTZ 300 mg/25 mg daily | 2447894 | 90 | 90 | 0.2184 |  | 19.656 | 1.57248 | 11 | 32.22848 | 12.57248 | 22.559936 | 9.668544 |  | Government share | 383.727008 |
| New Brunswick | Levothyroxine 50 mcg daily | 2213192 | 90 | 90 |  | 0.0311 | 2.879 | 0 | 11 | 13.879 | 11 | 9.7153 | 4.1637 |  | Patient share | 3841.590505 |
| New Brunswick | Atenolol 50mg daily | 2255545 | 90 | 90 | 0.1107 |  | 9.963 | 0.79704 | 11 | 21.76004 | 11.79704 | 15.232028 | 6.528012 |  |  |  |
| New Brunswick | Liraglutide inj 1.8mg daily | 2351064 | 90 | 27 |  | 29.7367 | 802.8909 | 64.231272 | 11 | 878.122172 | 75.231272 | 0 | 878.122172 |  |  |  |
| New Brunswick | Lorazepam 1mg QHS | 655759 | 90 | 90 | 0.0447 |  | 4.023 | 0.32184 | 11 | 15.34484 | 11.32184 | 10.741388 | 4.603452 |  |  |  |
| New Brunswick | ASAEC 81mg daily | 2237726 | 90 | 90 |  | 0.1095 | 9.855 | 5.306538462 | 0 | 15.16153846 | 5.306538462 | 0 | 15.16153846 |  |  |  |
| New Brunswick | Calcium 500mg / Vitamin D 1000U BID | 80017748 | 90 | 180 |  | 0.09389 | 16.9002 | 9.100107692 | 0 | 26.00030769 | 9.100107692 | 0 | 26.00030769 |  |  |  |
| New Brunswick/ Total Q1 |  |  |  |  |  |  |  |  |  |  | 160.6872782 | 95.931752 | 960.3976262 |  |  |  |
| Newfoundland and Labrador | Metformin 1000mg BID | 2167786 | 90 | 360 |  | 0.0269 | 8.9253456 | 0.758654378 | 12 | 21.684 | 12.75865438 | 15.684 | 6 |  | Newfoundland and Labrador / Annual costs |  |
| Newfoundland and Labrador | Omeprazole 20mg daily | 2245058 | 90 | 90 |  | 0.2493 | 20.679263 | 1.757737327 | 12 | 34.437 | 13.75773733 | 28.437 | 6 |  | Pharmacy Margin | 817.1787556 |
| Newfoundland and Labrador | Irbesartan/HCTZ 300 mg/25 mg daily | 2447894 | 90 | 90 |  | 0.2381 | 19.75023 | 1.678769585 | 12 | 33.429 | 13.67876959 | 27.429 | 6 |  | Government share | 431.352 |
| Newfoundland and Labrador | Levothyroxine 50 mcg daily | 2213192 | 90 | 90 |  | 0.0338 | 2.8036866 | 0.238313364 | 12 | 15.042 | 12.23831336 | 9.042 | 6 |  | Patient share | 3969.251709 |
| Newfoundland and Labrador | Atenolol 50mg daily | 2255545 | 90 | 90 |  | 0.1207 | 10.011982 | 0.851018433 | 12 | 22.863 | 12.85101843 | 16.863 | 6 |  |  |  |
| Newfoundland and Labrador | Liraglutide inj 1.8mg daily | 2351064 | 90 | 27 |  | 29.7367 | 802.8909 | 72.260181 | 40 | 915.151081 | 112.260181 | 0 | 915.151081 |  |  |  |
| Newfoundland and Labrador | Lorazepam 1mg QHS | 655759 | 90 | 90 |  | 0.0487 | 4.0396313 | 0.343368664 | 12 | 16.383 | 12.34336866 | 10.383 | 6 |  |  |  |
| Newfoundland and Labrador | ASAEC 81mg daily | 2237726 | 90 | 90 |  | 0.1095 | 9.855 | 5.306538462 | 0 | 15.16153846 | 5.306538462 | 0 | 15.16153846 |  |  |  |
| Newfoundland and Labrador | Calcium 500mg / Vitamin D 1000U BID | 80017748 | 90 | 180 |  | 0.09389 | 16.9002 | 9.100107692 | 0 | 26.00030769 | 9.100107692 | 0 | 26.00030769 |  |  |  |
| Newfoundland and Labrador/ Total Q1 |  |  |  |  |  |  |  |  |  |  | 204.2946889 | 107.838 | 992.3129272 |  |  |  |
| Nova Scotia | Metformin 1000mg BID | 2167786 | 90 | 360 | 0.0247 |  | 8.892 | 0.71136 | 11.95 | 21.55336 | 12.66136 | 15.087352 | 6.466008 |  | Nova Scotia /Annual costs |  |
| Nova Scotia | Omeprazole 20mg daily | 2245058 | 90 | 90 | 0.2287 |  | 20.583 | 1.64664 | 11.95 | 34.17964 | 13.59664 | 23.925748 | 10.253892 |  | Pharmacy Margin | 750.8326826 |
| Nova Scotia | Irbesartan/HCTZ 300 mg/25 mg daily | 2447894 | 90 | 90 | 0.2184 |  | 19.656 | 1.57248 | 11.95 | 33.17848 | 13.52248 | 23.224936 | 9.953544 |  | Government share | 400.425144 |
| Nova Scotia | Levothyroxine 50 mcg daily | 2213192 | 90 | 90 |  |  | 2.844 | 0.29862 | 11.95 | 15.09262 | 12.24862 | 10.564834 | 4.527786 |  | Patient share | 3932.835939 |
| Nova Scotia | Atenolol 50mg daily | 2255545 | 90 | 90 | 0.1107 |  | 9.963 | 0.79704 | 11.95 | 22.71004 | 12.74704 | 15.897028 | 6.813012 |  |  |  |
| Nova Scotia | Liraglutide inj 1.8mg daily | 2351064 | 90 | 27 |  | 29.7367 | 802.8909 | 84.3035445 | 11.95 | 899.1444445 | 96.2535445 | 0 | 899.1444445 |  |  |  |
| Nova Scotia | Lorazepam 1mg QHS | 655759 | 90 | 90 | 0.0447 |  | 4.023 | 0.32184 | 11.95 | 16.29484 | 12.27184 | 11.406388 | 4.888452 |  |  |  |
| Nova Scotia | ASAEC 81mg daily | 2237726 | 90 | 90 |  | 0.1095 | 9.855 | 5.306538462 | 0 | 15.16153846 | 5.306538462 | 0 | 15.16153846 |  |  |  |
| Nova Scotia | Calcium 500mg / Vitamin D 1000U BID | 80017748 | 90 | 180 |  | 0.09389 | 16.9002 | 9.100107692 | 0 | 26.00030769 | 9.100107692 | 0 | 26.00030769 |  |  |  |
| Nova Scotia/ Total Q1 |  |  |  |  |  |  |  |  |  |  | 187.7081707 | 100.106286 | 983.2089847 |  |  |  |
| Ontario | Metformin 1000mg BID | 2167786 | 90 | 360 |  | 0.0247 | 8.892 | 0.71136 | 8.83 | 18.43336 | 9.54136 | 12.32336 | 6.11 |  | Ontario /Annual costs |  |
| Ontario | Omeprazole 20mg daily | 2245058 | 90 | 90 |  | 0.2287 | 20.583 | 1.64664 | 8.83 | 31.05964 | 10.47664 | 24.94964 | 6.11 |  | Pharmacy Margin | 582.8991926 |
| Ontario | Irbesartan/HCTZ 300 mg/25 mg daily | 2447894 | 90 | 90 |  | 0.2184 | 19.656 | 1.57248 | 8.83 | 30.05848 | 10.40248 | 23.94848 | 6.11 |  | Government share | 350.23152 |
| Ontario | Levothyroxine 50 mcg daily | 2213192 | 90 | 90 |  | 0.0316 | 2.844 | 0.22752 | 8.83 | 11.90152 | 9.05752 | 5.79152 | 6.11 |  | Patient share | 3815.096073 |
| Ontario | Atenolol 50mg daily | 2255545 | 90 | 90 |  | 0.1107 | 9.963 | 0.79704 | 8.83 | 19.59004 | 9.62704 | 13.48004 | 6.11 |  |  |  |
| Ontario | Liraglutide inj 1.8mg daily | 2351064 | 90 | 27 |  | 29.7367 | 802.8909 | 64.231272 | 8.83 | 875.952172 | 73.061272 | 0 | 875.952172 |  |  |  |
| Ontario | Lorazepam 1mg QHS | 655759 | 90 | 90 |  | 0.0447 | 4.023 | 0.32184 | 8.83 | 13.17484 | 9.15184 | 7.06484 | 6.11 |  |  |  |
| Ontario | ASAEC 81mg daily | 2237726 | 90 | 90 |  | 0.1095 | 9.855 | 5.306538462 | 0 | 15.16153846 | 5.306538462 | 0 | 15.16153846 |  |  |  |
| Ontario | Calcium 500mg / Vitamin D 1000U BID | 80017748 | 90 | 180 |  | 0.09389 | 16.9002 | 9.100107692 | 0 | 26.00030769 | 9.100107692 | 0 | 26.00030769 |  |  |  |
| Ontario/ Total Q1 |  |  |  |  |  |  |  |  |  |  | 145.7247982 | 87.55788 | 953.7740182 |  |  |  |
| Prince Edward Island | Metformin 1000mg BID | 2167786 | 90 | 360 | 0.0247 |  | 8.892 | 0.53352 | 12.36 | 21.78552 | 12.89352 | 5.84552 | 15.94 |  | Prince Edward Island / Annual costs |  |
| Prince Edward Island | Omeprazole 20mg daily | 2245058 | 90 | 90 | 0.2287 |  | 20.583 | 1.23498 | 12.36 | 34.17798 | 13.59498 | 18.23798 | 15.94 |  | Pharmacy Margin | 612.2180806 |
| Prince Edward Island | Irbesartan/HCTZ 300 mg/25 mg daily | 2447894 | 90 | 90 | 0.2184 |  | 19.656 | 1.17936 | 12.36 | 33.19536 | 13.53936 | 17.25536 | 15.94 |  | Government share | 232.27368 |
| Prince Edward Island | Levothyroxine 50 mcg daily | 2213192 | 90 | 90 |  | 0.031 | 2.79 | 0.1674 | 12.36 | 15.3174 | 12.5274 | 4.8374 | 10.48 |  | Patient share | 3962.156801 |
| Prince Edward Island | Atenolol 50mg daily | 2255545 | 90 | 90 | 0.1107 |  | 9.963 | 0.59778 | 12.36 | 22.92078 | 12.95778 | 6.98078 | 15.94 |  |  |  |
| Prince Edward Island | Liraglutide inj 1.8mg daily | 2351064 | 90 | 27 |  | 29.7367 | 802.8909 | 48.173454 | 12.36 | 863.424354 | 60.533454 | 0 | 863.424354 |  |  |  |
| Prince Edward Island | Lorazepam 1mg QHS | 655759 | 90 | 90 | 0.0447 |  | 4.023 | 0.24138 | 12.36 | 16.62438 | 12.60138 | 4.91138 | 11.713 |  |  |  |
| Prince Edward Island | ASAEC 81mg daily | 2237726 | 90 | 90 |  | 0.1095 | 9.855 | 5.306538462 | 0 | 15.16153846 | 5.306538462 | 0 | 15.16153846 |  |  |  |
| Prince Edward Island | Calcium 500mg / Vitamin D 1000U BID | 80017748 | 90 | 180 |  | 0.09389 | 16.9002 | 9.100107692 | 0 | 26.00030769 | 9.100107692 | 0 | 26.00030769 |  |  |  |
| Prince Edward Island/ Total Q1 |  |  |  |  |  |  |  |  |  |  | 153.0545202 | 58.06842 | 990.5392002 |  |  |  |
| Quebec | Metformin 1000mg BID | 2167786 | 90 | 360 |  | 0.0247 | 8.892 | 0.57798 | 27 | 36.46998 | 27.57798 | 23.74195698 | 12.72802302 |  | Quebec /Annual costs |  |
| Quebec | Omeprazole 20mg daily | 2245058 | 90 | 90 |  | 0.2287 | 20.583 | 1.337895 | 27 | 48.920895 | 28.337895 | 31.84750265 | 17.07339236 |  | Pharmacy Margin | 990.9447646 |
| Quebec | Irbesartan/HCTZ 300 mg/25 mg daily | 2447894 | 90 | 90 |  | 0.2184 | 19.656 | 1.27764 | 27 | 47.93364 | 28.27764 | 31.20479964 | 16.72884036 |  | Government share | 2383.499907 |
| Quebec | Levothyroxine 50 mcg daily | 2213192 | 90 | 90 |  | 0.0274 | 2.466 | 0.16029 | 27 | 29.62629 | 27.16029 | 19.28671479 | 10.33957521 |  | Patient share | 1442.437657 |
| Quebec | Atenolol 50mg daily | 2255545 | 90 | 90 |  | 0.1107 | 9.963 | 0.647595 | 27 | 37.610595 | 27.647595 | 24.48449735 | 13.12609766 |  |  |  |
| Quebec | Liraglutide inj 1.8mg daily | 2351064 | 90 | 27 |  | 22.83 | 616.41 | 40.06665 | 27 | 683.47665 | 67.06665 | 444.9432992 | 238.5333509 |  |  |  |
| Quebec | Lorazepam 1mg QHS | 655759 | 90 | 90 |  | 0.0447 | 4.023 | 0.261495 | 27 | 31.284495 | 27.261495 | 20.36620625 | 10.91828876 |  |  |  |
| Quebec | ASAEC 81mg daily | 2237726 | 90 | 90 |  | 0.1095 | 9.855 | 5.306538462 | 0 | 15.16153846 | 5.306538462 | 0 | 15.16153846 |  |  |  |
| Quebec | Calcium 500mg / Vitamin D 1000U BID | 80017748 | 90 | 180 |  | 0.09389 | 16.9002 | 9.100107692 | 0 | 26.00030769 | 9.100107692 | 0 | 26.00030769 |  |  |  |
| Quebec/Total Q1 |  |  |  |  |  |  |  |  |  |  | 247.7361912 | 595.8749768 | 360.6094144 |  |  |  |
| Saskatchewan | Metformin 1000mg BID | 2167786 | 90 | 360 |  | 0.0247 | 8.892 | 1.3338 | 11.4 | 21.6258 | 12.7338 | 0 | 21.6258 |  | Saskatchewan /Annual costs |  |
| Saskatchewan | Omeprazole 20mg daily | 2245058 | 90 | 90 |  | 0.2287 | 20.583 | 2.0583 | 11.4 | 34.0413 | 13.4583 | 9.0413 | 25 |  | Pharmacy Margin | 492.4755846 |
| Saskatchewan | Irbesartan/HCTZ 300 mg/25 mg daily | 2447894 | 90 | 90 |  | 0.2184 | 19.656 | 1.9656 | 11.4 | 33.0216 | 13.3656 | 8.0216 | 25 |  | Government share | 68.2516 |
| Saskatchewan | Levothyroxine 50 mcg daily | 2213192 | 90 | 90 |  | 0.0316 | 2.844 | 0.8532 | 11.4 | 15.0972 | 12.2532 | 0 | 15.0972 |  | Patient share | 4006.652385 |
| Saskatchewan | Atenolol 50mg daily | 2255545 | 90 | 90 |  | 0.1107 | 9.963 | 1.49445 | 11.4 | 22.85745 | 12.89445 | 0 | 22.85745 |  |  |  |
| Saskatchewan | Liraglutide inj 1.8mg daily | 2351064 | 90 | 27 |  | 29.7367 | 802.8909 | 20 | 11.4 | 834.2909 | 31.4 | 0 | 834.2909 |  |  |  |
| Saskatchewan | Lorazepam 1mg QHS | 655759 | 90 | 90 |  | 0.0447 | 4.023 | 1.2069 | 11.4 | 16.6299 | 12.6069 | 0 | 16.6299 |  |  |  |
| Saskatchewan | ASAEC 81mg daily | 2237726 | 90 | 90 |  | 0.1095 | 9.855 | 5.306538462 | 0 | 15.16153846 | 5.306538462 | 0 | 15.16153846 |  |  |  |
| Saskatchewan | Calcium 500mg / Vitamin D 1000U BID | 80017748 | 90 | 180 |  | 0.09389 | 16.9002 | 9.100107692 | 0 | 26.00030769 | 9.100107692 | 0 | 26.00030769 |  |  |  |
| Saskatchewan/ Total Q1 |  |  |  |  |  |  |  |  |  |  | 123.1188962 | 17.0629 | 1001.663096 |  |  |  |
| Northwest Territories | Metformin 1000mg BID | 2167786 | 90 | 360 | 0 | 0.0247 | 8.892 | 1.6325712 | 12.72 | 23.2445712 | 14.3525712 | 23.2445712 | 0 |  | Northwest Territories /Annual costs |  |
| Northwest Territories | Omeprazole 20mg daily | 2245058 | 90 | 90 | 0 | 0.2287 | 20.583 | 3.7790388 | 12.72 | 37.0820388 | 16.4990388 | 37.0820388 | 0 |  | Pharmacy Margin | 662.1886854 |
| Northwest Territories | Irbesartan/HCTZ 300 mg/25 mg daily | 2447894 | 90 | 90 | 0 | 0.2184 | 19.656 | 3.6088416 | 12.72 | 35.9848416 | 16.3288416 | 35.9848416 | 0 |  | Government share | 617.3101008 |
| Northwest Territories | Levothyroxine 50 mcg daily | 2213192 | 90 | 90 | 0 | 0.031 | 2.79 | 0.512244 | 12.72 | 16.022244 | 13.232244 | 16.022244 | 0 |  | Patient share | 3627.090985 |
| Northwest Territories | Atenolol 50mg daily | 2255545 | 90 | 90 | 0 | 0.1107 | 9.963 | 1.8292068 | 12.72 | 24.5122068 | 14.5492068 | 24.5122068 | 0 |  |  |  |
| Northwest Territories | Liraglutide inj 1.8mg daily | 2351064 | 90 | 27 | 0 | 29.7367 | 802.8909 | 50 | 12.72 | 865.6109 | 62.72 | 0 | 865.6109 |  |  |  |
| Northwest Territories | Lorazepam 1mg QHS | 655759 | 90 | 90 | 0 | 0.0447 | 4.023 | 0.7386228 | 12.72 | 17.4816228 | 13.4586228 | 17.4816228 | 0 |  |  |  |
| Northwest Territories | ASAEC 81mg daily | 2237726 | 90 | 90 | 0 | 0.1095 | 9.855 | 5.306538462 | 0 | 15.16153846 | 5.306538462 | 0 | 15.16153846 |  |  |  |
| Northwest Territories | Calcium 500mg / Vitamin D 1000U BID | 80017748 | 90 | 180 | 0 | 0.09389 | 16.9002 | 9.100107692 | 0 | 26.00030769 | 9.100107692 | 0 | 26.00030769 |  |  |  |
| Northwest Territories |  |  |  |  |  |  |  |  |  |  | 165.5471714 | 154.3275252 | 906.7727462 |  |  |  |
| Nunavut | Metformin 1000mg BID | 2167786 | 90 | 360 | 0 | 0.0247 | 8.892 | 2.1127392 | 16.95 | 27.9547392 | 19.0627392 | 27.9547392 | 0 |  | Nunavut /Annual costs |  |
| Nunavut | Omeprazole 20mg daily | 2245058 | 90 | 90 | 0 | 0.2287 | 20.583 | 4.8905208 | 16.95 | 42.4235208 | 21.8405208 | 42.4235208 | 0 |  | Pharmacy Margin | 794.8645974 |
| Nunavut | Irbesartan/HCTZ 300 mg/25 mg daily | 2447894 | 90 | 90 | 0 | 0.2184 | 19.656 | 4.6702656 | 16.95 | 41.2762656 | 21.6202656 | 41.2762656 | 0 |  | Government share | 733.0660128 |
| Nunavut | Levothyroxine 50 mcg daily | 2213192 | 90 | 90 | 0 | 0.031 | 2.79 | 0.662904 | 16.95 | 20.402904 | 17.612904 | 20.402904 | 0 |  | Patient share | 3644.010985 |
| Nunavut | Atenolol 50mg daily | 2255545 | 90 | 90 | 0 | 0.1107 | 9.963 | 2.3672088 | 16.95 | 29.2802088 | 19.3172088 | 29.2802088 | 0 |  |  |  |
| Nunavut | Liraglutide inj 1.8mg daily | 2351064 | 90 | 27 | 0 | 29.7367 | 802.8909 | 50 | 16.95 | 869.8409 | 66.95 | 0 | 869.8409 |  |  |  |
| Nunavut | Lorazepam 1mg QHS | 655759 | 90 | 90 | 0 | 0.0447 | 4.023 | 0.9558648 | 16.95 | 21.9288648 | 17.9058648 | 21.9288648 | 0 |  |  |  |
| Nunavut | ASAEC 81mg daily | 2237726 | 90 | 90 | 0 | 0.1095 | 9.855 | 5.306538462 | 0 | 15.16153846 | 5.306538462 | 0 | 15.16153846 |  |  |  |
| Nunavut | Calcium 500mg / Vitamin D 1000U BID | 80017748 | 90 | 180 | 0 | 0.09389 | 16.9002 | 9.100107692 | 0 | 26.00030769 | 9.100107692 | 0 | 26.00030769 |  |  |  |
| Nunavut |  |  |  |  |  |  |  |  |  |  | 198.7161494 | 183.2665032 | 911.0027462 |  |  |  |
| Yukon | Metformin 1000mg BID | 2167786 | 90 | 360 |  | 0.0247 | 8.892 | 2.1127392 | 12.72 | 23.7247392 | 14.8327392 | 23.7247392 | 0 |  | **Yukon /Annual costs** |  |
| Yukon | Omeprazole 20mg daily | 2245058 | 90 | 90 |  | 0.2287 | 20.583 | 4.8905208 | 12.72 | 38.1935208 | 17.6105208 | 38.1935208 | 0 |  | Pharmacy Margin | 712.4337684 |
| Yukon | Irbesartan/HCTZ 300 mg/25 mg daily | 2357410 | 90 | 90 |  | 0.2184 | 19.656 | 4.6702656 | 12.72 | 37.0462656 | 17.3902656 | 37.0462656 | 0 |  | Government share | 715.5293376 |
| Yukon | Levothyroxine 50 mcg daily | 2213192 | 90 | 90 |  | 0.03 | 2.7 | 0.64152 | 12.72 | 16.06152 | 13.36152 | 16.06152 | 0 |  | Patient share | 3566.444831 |
| Yukon | Atenolol 50mg daily | 2255545 | 90 | 90 |  | 0.1107 | 9.963 | 2.3672088 | 12.72 | 25.0502088 | 15.0872088 | 25.0502088 | 0 |  |  |  |
| Yukon | Liraglutide inj 1.8mg daily | 2351064 | 90 | 27 |  | 29.7367 | 802.8909 | 50 | 12.72 | 865.6109 | 62.72 | 0 | 865.6109 |  |  |  |
| Yukon | Lorazepam 1mg QHS | 655759 | 90 | 90 |  | 0.04 | 3.6 | 0.85536 | 12.72 | 17.17536 | 13.57536 | 17.17536 | 0 |  |  |  |
| Yukon | ASAEC 81mg daily | 2237726 | 90 | 90 |  | 0.08 | 7.2 | 1.71072 | 12.72 | 21.63072 | 14.43072 | 21.63072 | 0 |  |  |  |
| Yukon | Calcium 500mg / Vitamin D 1000U BID | 80017748 | 90 | 180 |  | 0.09389 | 16.9002 | 9.100107692 | 0 | 26.00030769 | 9.100107692 | 0 | 26.00030769 |  |  |  |
| Yukon |  |  |  |  |  |  |  |  |  |  | 178.1084421 | 178.8823344 | 891.6112077 |  |  |  |

Supplementary Table 4 – Scenario 3

| Province | Drug | DIN | Days Supply | Quantity | MAC/Unit | Drug Cost / Unit | Drug Cost | Markup | Dispensing Fee | Total Cost | Pharmacy Margin $ | Government Share | Patient Share |  |  |  |
| --- | --- | --- | --- | --- | --- | --- | --- | --- | --- | --- | --- | --- | --- | --- | --- | --- |
| Alberta | Metformin 1000mg BID | 2167786 | 90 | 360 |  | 0.0247 | 8.892 | 0.9078732 | 12.15 | 21.9498732 | 13.0578732 | 15.36491124 | 6.58496196 |  |  |  |
| Alberta | Atorvastatin 40mg daily | 2295296 | 90 | 90 |  | 0.2342 | 21.078 | 2.1520638 | 12.15 | 35.3800638 | 14.3020638 | 24.76604466 | 10.61401914 |  | Alberta /Annual costs |  |
| Alberta | Omeprazole 20mg daily | 2245058 | 90 | 90 | 0 | 0.2287 | 20.583 | 2.1015243 | 12.15 | 34.8345243 | 14.2515243 | 24.38416701 | 10.45035729 |  | Pharmacy Margin | 951.448735 |
| Alberta | Irbesartan/HCTZ 300 mg/25 mg daily | 2447894 | 90 | 90 |  | 0.2184 | 19.656 | 2.0068776 | 12.15 | 33.8128776 | 14.1568776 | 23.66901432 | 10.14386328 |  | Government Share | 491.3357535 |
| Alberta | Levothyroxine 50 mcg daily | 2213192 | 90 | 90 |  | 0.031 | 2.79 | 0.284859 | 12.15 | 15.224859 | 12.434859 | 10.6574013 | 4.5674577 |  | Patient Share | 4117.920881 |
| Alberta | Atenolol 50mg daily | 2255545 | 90 | 90 |  | 0.1107 | 9.963 | 1.0172223 | 12.15 | 23.1302223 | 13.1672223 | 16.19115561 | 6.93906669 |  |  |  |
| Alberta | Liraglutide inj 1.8mg daily | 2351064 | 90 | 27 |  | 29.7367 | 802.8909 | 120.6343577 | 12.15 | 935.6752577 | 132.7843577 | 0 | 935.6752577 |  |  |  |
| Alberta | Lorazepam 1mg QHS | 655759 | 90 | 90 |  | 0.0447 | 4.023 | 0.4107483 | 12.15 | 16.5837483 | 12.5607483 | 11.60862381 | 4.97512449 |  |  |  |
| ***Q2*** | Lorazepam 1mg QHS |  |  | **45** |  | 0.0447 | 2.0115 | 0.20537415 | 12.15 | 14.36687415 | 12.35537415 | 10.05681191 | 4.310062245 |  |  |  |
| ***Q3*** | Lorazepam 1mg QHS |  |  | **30** |  | 0.0447 | 1.341 | 0.1369161 | 12.15 | 13.6279161 | 12.2869161 | 9.53954127 | 4.08837483 |  |  |  |
| ***Q4*** | Lorazepam 1mg QHS |  |  | **0** |  | 0.0447 | 0 | 0 | 0 | 0 | 0 | 0 | 0 |  |  |  |
| Alberta | ASAEC 81mg daily | 2237726 | 90 | 90 |  | 0.1095 | 9.855 | 5.306538462 | 0 | 15.16153846 | 5.306538462 | 0 | 15.16153846 |  |  |  |
| Alberta | Calcium 500mg / Vitamin D 1000U BID | 80017748 | 90 | 180 |  | 0.09389 | 16.9002 | 9.100107692 | 0 | 26.00030769 | 9.100107692 | 0 | 26.00030769 |  |  |  |
| Alberta/ Total Q1 |  |  |  |  |  |  |  |  |  |  | 241.1221724 | 126.641318 | 1031.111954 |  |  |  |
| British Columbia | Metformin 1000mg BID | 2167786 | 90 | 360 | 0.0267 |  | 8.9 | 0.712 | 10 | 19.612 | 10.712 | 0 | 19.612 |  |  |  |
| British Columbia | Atorvastatin 40mg daily | 2295296 | 90 | 90 | 0.2529 |  | 21.075 | 1.686 | 10 | 32.761 | 11.686 | 0 | 32.761 |  | British Columbia /Annual costs |  |
| British Columbia | Omeprazole 20mg daily | 2245058 | 90 | 90 | 0.2025 |  | 16.875 | 1.35 | 10 | 28.225 | 11.35 | 0 | 28.225 |  | Pharmacy Margin | 673.0171854 |
| British Columbia | Irbesartan/HCTZ 300 mg/25 mg daily | 2447894 | 90 | 90 | 0.2719 |  | 22.65833 | 1.812666667 | 10 | 34.471 | 11.81266667 | 0 | 34.471 |  | Government Share | 0 |
| British Columbia | Levothyroxine 50 mcg daily | 2213192 | 90 | 90 | 0.0341 |  | 2.841667 | 0.227333333 | 10 | 13.069 | 10.22733333 | 0 | 13.069 |  | Patient Share | 4323.32742 |
| British Columbia | Atenolol 50mg daily | 2255545 | 90 | 90 | 0.1196 |  | 9.966667 | 0.797333333 | 10 | 20.764 | 10.79733333 | 0 | 20.764 |  |  |  |
| British Columbia | Liraglutide inj 1.8mg daily | 2351064 | 90 | 27 |  | 29.7367 | 802.8909 | 64.231272 | 10 | 877.122172 | 74.231272 | 0 | 877.122172 |  |  |  |
| British Columbia | Lorazepam 1mg QHS | 655759 | 90 | 90 | 0.0483 |  | 4.025 | 0.322 | 10 | 14.347 | 10.322 | 0 | 14.347 |  |  |  |
| ***Q2*** | Lorazepam 1mg QHS |  |  | **45** | 0.0483 |  | 2.0125 | 0.161 | 10 | 12.1735 | 10.161 | 0 | 12.1735 |  |  |  |
| ***Q3*** | Lorazepam 1mg QHS |  |  | **30** | 0.0483 |  | 1.341667 | 0.107333333 | 10 | 11.449 | 10.10733333 | 0 | 11.449 |  |  |  |
| ***Q4*** | Lorazepam 1mg QHS |  |  | **0** |  |  | 0 | 0 | 0 | 0 | 0 | 0 | 0 |  |  |  |
| British Columbia | ASAEC 81mg daily | 2237726 | 90 | 90 | 0.1035 |  | 8.625 | 0.69 | 10 | 19.315 | 10.69 | 0 | 19.315 |  |  |  |
| British Columbia | Calcium 500mg / Vitamin D 1000U BID | 80017748 | 90 | 180 |  | 0.09389 | 16.9002 | 9.100107692 | 0 | 26.00030769 | 9.100107692 | 0 | 26.00030769 |  |  |  |
| British Columbia/ Total Q1 |  |  |  |  |  |  |  |  |  |  | 170.928713 | 0 | 1085.68648 |  |  |  |
| Manitoba | Metformin 1000mg BID | 2167786 | 90 | 360 |  | 0.0259 | 9.324 | 0 | 13.65 | 22.974 | 13.65 | 22.974 | 0 |  |  |  |
| Manitoba | Atorvastatin 40mg daily | 2295296 | 90 | 90 |  | 0.2459 | 22.131 | 0 | 13.65 | 35.781 | 13.65 | 35.781 | 0 |  | Manitoba /Annual costs |  |
| Manitoba | Omeprazole 20mg daily | 2245058 | 90 | 90 |  | 0.2401 | 21.609 | 0 | 13.65 | 35.259 | 13.65 | 35.259 | 0 |  | Pharmacy Margin | 480.2306032 |
| Manitoba | Irbesartan/HCTZ 300 mg/25 mg daily | 2447894 | 90 | 90 |  | 0.2293 | 20.637 | 0 | 13.65 | 34.287 | 13.65 | 34.287 | 0 |  | Government Share | 724.464 |
| Manitoba | Levothyroxine 50 mcg daily | 2213192 | 90 | 90 |  | 0.031 | 2.79 | 0 | 13.65 | 16.44 | 13.65 | 16.44 | 0 |  | Patient Share | 3430.810985 |
| Manitoba | Atenolol 50mg daily | 2255545 | 90 | 90 |  | 0.1162 | 10.458 | 0 | 13.65 | 24.108 | 13.65 | 24.108 | 0 |  |  |  |
| Manitoba | Liraglutide inj 1.8mg daily | 2351064 | 90 | 27 |  | 29.7367 | 802.8909 | 0 | 13.65 | 816.5409 | 13.65 | 0 | 816.5409 |  |  |  |
| Manitoba | Lorazepam 1mg QHS | 655759 | 90 | 90 |  | 0.0492 | 4.428 | 0 | 13.65 | 18.078 | 13.65 | 18.078 | 0 |  |  |  |
| ***Q2*** | Lorazepam 1mg QHS |  |  | **45** |  | 0.0492 | 2.214 | 0 | 13.65 | 15.864 | 13.65 | 15.864 | 0 |  |  |  |
| ***Q3*** | Lorazepam 1mg QHS |  |  | **30** |  | 0.0492 | 1.476 | 0 | 13.65 | 15.126 | 13.65 | 15.126 | 0 |  |  |  |
| ***Q4*** | Lorazepam 1mg QHS |  |  | **0** |  | 0.0492 | 0 | 0 | 0 | 0 | 0 | 0 | 0 |  |  |  |
| Manitoba | ASAEC 81mg daily | 2237726 | 90 | 90 |  | 0.1095 | 9.855 | 5.306538462 | 0 | 15.16153846 | 5.306538462 | 0 | 15.16153846 |  |  |  |
| Manitoba | Calcium 500mg / Vitamin D 1000U BID | 80017748 | 90 | 180 |  | 0.09389 | 16.9002 | 9.100107692 | 0 | 26.00030769 | 9.100107692 | 0 | 26.00030769 |  |  |  |
| Manitoba/ Total Q1 |  |  |  |  |  |  |  |  |  |  | 123.6066462 | 186.927 | 857.7027462 |  |  |  |
| New Brunswick | Metformin 1000mg BID | 2167786 | 90 | 360 | 0.0247 |  | 8.892 | 0.71136 | 11 | 20.60336 | 11.71136 | 14.422352 | 6.181008 |  |  |  |
| New Brunswick | Atorvastatin 40mg daily | 2295296 | 90 | 90 | 0.2342 |  | 21.078 | 1.68624 | 11 | 33.76424 | 12.68624 | 23.634968 | 10.129272 |  | New Brunswick /Annual costs |  |
| New Brunswick | Omeprazole 20mg daily | 2245058 | 90 | 90 | 0.2287 |  | 20.583 | 1.64664 | 11 | 33.22964 | 12.64664 | 23.260748 | 9.968892 |  | Pharmacy Margin | 681.7967528 |
| New Brunswick | Irbesartan/HCTZ 300 mg/25 mg daily | 2447894 | 90 | 90 | 0.2184 |  | 19.656 | 1.57248 | 11 | 32.22848 | 12.57248 | 22.559936 | 9.668544 |  | Government Share | 463.977206 |
| New Brunswick | Levothyroxine 50 mcg daily | 2213192 | 90 | 90 |  | 0.0311 | 2.879 | 0 | 11 | 13.879 | 11 | 9.7153 | 4.1637 |  | Patient Share | 3875.983447 |
| New Brunswick | Atenolol 50mg daily | 2255545 | 90 | 90 | 0.1107 |  | 9.963 | 0.79704 | 11 | 21.76004 | 11.79704 | 15.232028 | 6.528012 |  |  |  |
| New Brunswick | Liraglutide inj 1.8mg daily | 2351064 | 90 | 27 |  | 29.7367 | 802.8909 | 64.231272 | 11 | 878.122172 | 75.231272 | 0 | 878.122172 |  |  |  |
| New Brunswick | Lorazepam 1mg QHS | 655759 | 90 | 90 | 0.0447 |  | 4.023 | 0.32184 | 11 | 15.34484 | 11.32184 | 10.741388 | 4.603452 |  |  |  |
| ***Q2*** | Lorazepam 1mg QHS |  |  | **45** | 0.0447 |  | 2.0115 | 0.16092 | 11 | 13.17242 | 11.16092 | 9.220694 | 3.951726 |  |  |  |
| ***Q3*** | Lorazepam 1mg QHS |  |  | **30** | 0.0447 |  | 1.341 | 0.10728 | 11 | 12.44828 | 11.10728 | 8.713796 | 3.734484 |  |  |  |
| ***Q4*** | Lorazepam 1mg QHS |  |  | **0** | 0.0447 |  | 0 | 0 | 0 | 0 | 0 | 0 | 0 |  |  |  |
| New Brunswick | ASAEC 81mg daily | 2237726 | 90 | 90 |  | 0.1095 | 9.855 | 5.306538462 | 0 | 15.16153846 | 5.306538462 | 0 | 15.16153846 |  |  |  |
| New Brunswick | Calcium 500mg / Vitamin D 1000U BID | 80017748 | 90 | 180 |  | 0.09389 | 16.9002 | 9.100107692 | 0 | 26.00030769 | 9.100107692 | 0 | 26.00030769 |  |  |  |
| New Brunswick/ Total Q1 |  |  |  |  |  |  |  |  |  |  | 173.3735182 | 119.56672 | 970.5268982 |  |  |  |
| Newfoundland and Labrador | Metformin 1000mg BID | 2167786 | 90 | 360 |  | 0.0269 | 8.925346 | 0.758654378 | 12 | 21.684 | 12.75865438 | 15.684 | 6 |  |  |  |
| Newfoundland and Labrador | Atorvastatin 40mg daily | 2295296 | 90 | 90 |  | 0.2553 | 21.17696 | 1.800041475 | 12 | 34.977 | 13.80004147 | 28.977 | 6 |  | Newfoundland and Labrador / Annual costs |  |
| Newfoundland and Labrador | Omeprazole 20mg daily | 2245058 | 90 | 90 |  | 0.2493 | 20.67926 | 1.757737327 | 12 | 34.437 | 13.75773733 | 28.437 | 6 |  | Pharmacy Margin | 859.634956 |
| Newfoundland and Labrador | Irbesartan/HCTZ 300 mg/25 mg daily | 2447894 | 90 | 90 |  | 0.2381 | 19.75023 | 1.678769585 | 12 | 33.429 | 13.67876959 | 27.429 | 6 |  | Government Share | 531.7635 |
| Newfoundland and Labrador | Levothyroxine 50 mcg daily | 2213192 | 90 | 90 |  | 0.0338 | 2.803687 | 0.238313364 | 12 | 15.042 | 12.23831336 | 9.042 | 6 |  | Patient Share | 3987.251709 |
| Newfoundland and Labrador | Atenolol 50mg daily | 2255545 | 90 | 90 |  | 0.1207 | 10.01198 | 0.851018433 | 12 | 22.863 | 12.85101843 | 16.863 | 6 |  |  |  |
| Newfoundland and Labrador | Liraglutide inj 1.8mg daily | 2351064 | 90 | 27 |  | 29.7367 | 802.8909 | 72.260181 | 40 | 915.151081 | 112.260181 | 0 | 915.151081 |  |  |  |
| Newfoundland and Labrador | Lorazepam 1mg QHS | 655759 | 90 | 90 |  | 0.0487 | 4.039631 | 0.343368664 | 12 | 16.383 | 12.34336866 | 10.383 | 6 |  |  |  |
| ***Q2*** | Lorazepam 1mg QHS |  |  | **45** |  | 0.0487 | 2.019816 | 0.171684332 | 12 | 14.1915 | 12.17168433 | 8.1915 | 6 |  |  |  |
| ***Q3*** | Lorazepam 1mg QHS |  |  | **30** |  | 0.0487 | 1.346544 | 0.114456221 | 12 | 13.461 | 12.11445622 | 7.461 | 6 |  |  |  |
| ***Q4*** | Lorazepam 1mg QHS |  |  | **0** |  | 0.0487 | 0 | 0 | 0 | 0 | 0 | 0 | 0 |  |  |  |
| Newfoundland and Labrador | ASAEC 81mg daily | 2237726 | 90 | 90 |  | 0.1095 | 9.855 | 5.306538462 | 0 | 15.16153846 | 5.306538462 | 0 | 15.16153846 |  |  |  |
| Newfoundland and Labrador | Calcium 500mg / Vitamin D 1000U BID | 80017748 | 90 | 180 |  | 0.09389 | 16.9002 | 9.100107692 | 0 | 26.00030769 | 9.100107692 | 0 | 26.00030769 |  |  |  |
| Newfoundland and Labrador/ Total Q1 |  |  |  |  |  |  |  |  |  |  | 218.0947304 | 136.815 | 998.3129272 |  |  |  |
| Nova Scotia | Metformin 1000mg BID | 2167786 | 90 | 360 | 0.0247 |  | 8.892 | 0.71136 | 11.95 | 21.55336 | 12.66136 | 15.087352 | 6.466008 |  |  |  |
| Nova Scotia | Atorvastatin 40mg daily | 2295296 | 90 | 90 | 0.2342 |  | 21.078 | 1.68624 | 11.95 | 34.71424 | 13.63624 | 24.299968 | 10.414272 |  | Nova Scotia /Annual costs |  |
| Nova Scotia | Omeprazole 20mg daily | 2245058 | 90 | 90 | 0.2287 |  | 20.583 | 1.64664 | 11.95 | 34.17964 | 13.59664 | 23.925748 | 10.253892 |  | Pharmacy Margin | 792.7303228 |
| Nova Scotia | Irbesartan/HCTZ 300 mg/25 mg daily | 2447894 | 90 | 90 | 0.2184 |  | 19.656 | 1.57248 | 11.95 | 33.17848 | 13.52248 | 23.224936 | 9.953544 |  | Government Share | 482.670342 |
| Nova Scotia | Levothyroxine 50 mcg daily | 2213192 | 90 | 90 |  |  | 2.844 | 0.29862 | 11.95 | 15.09262 | 12.24862 | 10.564834 | 4.527786 |  | Patient Share | 3968.083881 |
| Nova Scotia | Atenolol 50mg daily | 2255545 | 90 | 90 | 0.1107 |  | 9.963 | 0.79704 | 11.95 | 22.71004 | 12.74704 | 15.897028 | 6.813012 |  |  |  |
| Nova Scotia | Liraglutide inj 1.8mg daily | 2351064 | 90 | 27 |  | 29.7367 | 802.8909 | 84.3035445 | 11.95 | 899.1444445 | 96.2535445 | 0 | 899.1444445 |  |  |  |
| Nova Scotia | Lorazepam 1mg QHS | 655759 | 90 | 90 | 0.0447 |  | 4.023 | 0.32184 | 11.95 | 16.29484 | 12.27184 | 11.406388 | 4.888452 |  |  |  |
| ***Q2*** | Lorazepam 1mg QHS |  |  | **45** | 0.0447 |  | 2.0115 | 0.16092 | 11.95 | 14.12242 | 12.11092 | 9.885694 | 4.236726 |  |  |  |
| ***Q3*** | Lorazepam 1mg QHS |  |  | **30** | 0.0447 |  | 1.341 | 0.10728 | 11.95 | 13.39828 | 12.05728 | 9.378796 | 4.019484 |  |  |  |
| ***Q4*** | Lorazepam 1mg QHS |  |  | **0** | 0.0447 |  | 0 | 0 | 0 | 0 | 0 | 0 | 0 |  |  |  |
| Nova Scotia | ASAEC 81mg daily | 2237726 | 90 | 90 |  | 0.1095 | 9.855 | 5.306538462 | 0 | 15.16153846 | 5.306538462 | 0 | 15.16153846 |  |  |  |
| Nova Scotia | Calcium 500mg / Vitamin D 1000U BID | 80017748 | 90 | 180 |  | 0.09389 | 16.9002 | 9.100107692 | 0 | 26.00030769 | 9.100107692 | 0 | 26.00030769 |  |  |  |
| Nova Scotia/ Total Q1 |  |  |  |  |  |  |  |  |  |  | 201.3444107 | 124.406254 | 993.6232567 |  |  |  |
| Ontario | Metformin 1000mg BID | 2167786 | 90 | 360 |  | 0.0247 | 8.892 | 0.71136 | 8.83 | 18.43336 | 9.54136 | 12.32336 | 6.11 |  |  |  |
| Ontario | Atorvastatin 40mg daily | 2295296 | 90 | 90 |  | 0.2342 | 21.078 | 1.68624 | 8.83 | 31.59424 | 10.51624 | 25.48424 | 6.11 |  | Ontario /Annual costs |  |
| Ontario | Omeprazole 20mg daily | 2245058 | 90 | 90 |  | 0.2287 | 20.583 | 1.64664 | 8.83 | 31.05964 | 10.47664 | 24.94964 | 6.11 |  | Pharmacy Margin | 615.4368328 |
| Ontario | Irbesartan/HCTZ 300 mg/25 mg daily | 2447894 | 90 | 90 |  | 0.2184 | 19.656 | 1.57248 | 8.83 | 30.05848 | 10.40248 | 23.94848 | 6.11 |  | Government Share | 440.03466 |
| Ontario | Levothyroxine 50 mcg daily | 2213192 | 90 | 90 |  | 0.0316 | 2.844 | 0.22752 | 8.83 | 11.90152 | 9.05752 | 5.79152 | 6.11 |  | Patient Share | 3833.426073 |
| Ontario | Atenolol 50mg daily | 2255545 | 90 | 90 |  | 0.1107 | 9.963 | 0.79704 | 8.83 | 19.59004 | 9.62704 | 13.48004 | 6.11 |  |  |  |
| Ontario | Liraglutide inj 1.8mg daily | 2351064 | 90 | 27 |  | 29.7367 | 802.8909 | 64.231272 | 8.83 | 875.952172 | 73.061272 | 0 | 875.952172 |  |  |  |
| Ontario | Lorazepam 1mg QHS | 655759 | 90 | 90 |  | 0.0447 | 4.023 | 0.32184 | 8.83 | 13.17484 | 9.15184 | 7.06484 | 6.11 |  |  |  |
| ***Q2*** | Lorazepam 1mg QHS |  |  | **45** |  | 0.0447 | 2.0115 | 0.16092 | 8.83 | 11.00242 | 8.99092 | 4.89242 | 6.11 |  |  |  |
| ***Q3*** | Lorazepam 1mg QHS |  |  | **30** |  | 0.0447 | 1.341 | 0.10728 | 8.83 | 10.27828 | 8.93728 | 4.16828 | 6.11 |  |  |  |
| ***Q4*** | Lorazepam 1mg QHS |  |  | **0** |  | 0.0447 | 0 | 0 | 0 | 0 | 0 | 0 | 0 |  |  |  |
| Ontario | ASAEC 81mg daily | 2237726 | 90 | 90 |  | 0.1095 | 9.855 | 5.306538462 | 0 | 15.16153846 | 5.306538462 | 0 | 15.16153846 |  |  |  |
| Ontario | Calcium 500mg / Vitamin D 1000U BID | 80017748 | 90 | 180 |  | 0.09389 | 16.9002 | 9.100107692 | 0 | 26.00030769 | 9.100107692 | 0 | 26.00030769 |  |  |  |
| Ontario/ Total Q1 |  |  |  |  |  |  |  |  |  |  | 156.2410382 | 113.04212 | 959.8840182 |  |  |  |
| Prince Edward Island | Metformin 1000mg BID | 2167786 | 90 | 360 | 0.0247 |  | 8.892 | 0.53352 | 12.36 | 21.78552 | 12.89352 | 5.84552 | 15.94 |  |  |  |
| Prince Edward Island | Atorvastatin 40mg daily | 2295296 | 90 | 90 | 0.2342 |  | 21.078 | 1.26468 | 12.36 | 34.70268 | 13.62468 | 18.76268 | 15.94 |  | Prince Edward Island / Annual costs |  |
| Prince Edward Island | Omeprazole 20mg daily | 2245058 | 90 | 90 | 0.2287 |  | 20.583 | 1.23498 | 12.36 | 34.17798 | 13.59498 | 18.23798 | 15.94 |  | Pharmacy Margin | 653.8338108 |
| Prince Edward Island | Irbesartan/HCTZ 300 mg/25 mg daily | 2447894 | 90 | 90 | 0.2184 |  | 19.656 | 1.17936 | 12.36 | 33.19536 | 13.53936 | 17.25536 | 15.94 |  | Government Share | 302.13141 |
| Prince Edward Island | Levothyroxine 50 mcg daily | 2213192 | 90 | 90 | 0 | 0.031 | 2.79 | 0.1674 | 12.36 | 15.3174 | 12.5274 | 4.8374 | 10.48 |  | Patient Share | 4009.5103 |
| Prince Edward Island | Atenolol 50mg daily | 2255545 | 90 | 90 | 0.1107 |  | 9.963 | 0.59778 | 12.36 | 22.92078 | 12.95778 | 6.98078 | 15.94 |  |  |  |
| Prince Edward Island | Liraglutide inj 1.8mg daily | 2351064 | 90 | 27 |  | 29.7367 | 802.8909 | 48.173454 | 12.36 | 863.424354 | 60.533454 | 0 | 863.424354 |  |  |  |
| Prince Edward Island | Lorazepam 1mg QHS | 655759 | 90 | 90 | 0.0447 |  | 4.023 | 0.24138 | 12.36 | 16.62438 | 12.60138 | 4.91138 | 11.713 |  |  |  |
| ***Q2*** | Lorazepam 1mg QHS |  |  | **45** | 0.0447 |  | 2.0115 | 0.12069 | 12.36 | 14.49219 | 12.48069 | 4.79069 | 9.7015 |  |  |  |
| ***Q3*** | Lorazepam 1mg QHS |  |  | **30** | 0.0447 |  | 1.341 | 0.08046 | 12.36 | 13.78146 | 12.44046 | 4.75046 | 9.031 |  |  |  |
| ***Q4*** | Lorazepam 1mg QHS |  |  | **0** | 0.0447 |  | 0 | 0 | 0 | 0 | 0 | 0 | 0 |  |  |  |
| Prince Edward Island | ASAEC 81mg daily | 2237726 | 90 | 90 |  | 0.1095 | 9.855 | 5.306538462 | 0 | 15.16153846 | 5.306538462 | 0 | 15.16153846 |  |  |  |
| Prince Edward Island | Calcium 500mg / Vitamin D 1000U BID | 80017748 | 90 | 180 |  | 0.09389 | 16.9002 | 9.100107692 | 0 | 26.00030769 | 9.100107692 | 0 | 26.00030769 |  |  |  |
| Prince Edward Island/ Total Q1 |  |  |  |  |  |  |  |  |  |  | 166.6792002 | 76.8311 | 1006.4792 |  |  |  |
| Quebec | Metformin 1000mg BID | 2167786 | 90 | 360 |  | 0.0247 | 8.892 | 0.57798 | 27 | 36.46998 | 27.57798 | 23.74195698 | 12.72802302 |  |  |  |
| Quebec | Atorvastatin 40mg daily | 2295296 | 90 | 90 |  | 0.2342 | 21.078 | 1.37007 | 27 | 49.44807 | 28.37007 | 32.19069357 | 17.25737643 |  | Quebec /Annual costs |  |
| Quebec | Omeprazole 20mg daily | 2245058 | 90 | 90 |  | 0.2287 | 20.583 | 1.337895 | 27 | 48.920895 | 28.337895 | 31.84750265 | 17.07339236 |  | Pharmacy Margin | 1076.858472 |
| Quebec | Irbesartan/HCTZ 300 mg/25 mg daily | 2447894 | 90 | 90 |  | 0.2184 | 19.656 | 1.27764 | 27 | 47.93364 | 28.27764 | 31.20479964 | 16.72884036 |  | Government Share | 2488.642401 |
| Quebec | Levothyroxine 50 mcg daily | 2213192 | 90 | 90 |  | 0.0274 | 2.466 | 0.16029 | 27 | 29.62629 | 27.16029 | 19.28671479 | 10.33957521 |  | Patient Share | 1498.804371 |
| Quebec | Atenolol 50mg daily | 2255545 | 90 | 90 |  | 0.1107 | 9.963 | 0.647595 | 27 | 37.610595 | 27.647595 | 24.48449735 | 13.12609766 |  |  |  |
| Quebec | Liraglutide inj 1.8mg daily | 2351064 | 90 | 27 |  | 22.83 | 616.41 | 40.06665 | 27 | 683.47665 | 67.06665 | 444.9432992 | 238.5333509 |  |  |  |
| Quebec | Lorazepam 1mg QHS | 655759 | 90 | 90 |  | 0.0447 | 4.023 | 0.261495 | 27 | 31.284495 | 27.261495 | 20.36620625 | 10.91828876 |  |  |  |
| ***Q2*** | Lorazepam 1mg QHS |  |  | **45** |  | 0.0447 | 2.0115 | 0.1307475 | 27 | 29.1422475 | 27.1307475 | 18.97160312 | 10.17064438 |  |  |  |
| ***Q3*** | Lorazepam 1mg QHS |  |  | **30** |  | 0.0447 | 1.341 | 0.087165 | 27 | 28.428165 | 27.087165 | 18.50673542 | 9.921429585 |  |  |  |
| ***Q4*** | Lorazepam 1mg QHS |  |  | **0** |  | 0.0447 | 0 | 0 | 0 | 0 | 0 | 0 | 0 |  |  |  |
| Quebec | ASAEC 81mg daily | 2237726 | 90 | 90 |  | 0.1095 | 9.855 | 5.306538462 | 0 | 15.16153846 | 5.306538462 | 0 | 15.16153846 |  |  |  |
| Quebec | Calcium 500mg / Vitamin D 1000U BID | 80017748 | 90 | 180 |  | 0.09389 | 16.9002 | 9.100107692 | 0 | 26.00030769 | 9.100107692 | 0 | 26.00030769 |  |  |  |
| Quebec/Total Q1 |  |  |  |  |  |  |  |  |  |  | 276.1062612 | 628.0656704 | 377.8667908 |  |  |  |
| Saskatchewan | Metformin 1000mg BID | 2167786 | 90 | 360 |  | 0.0247 | 8.892 | 1.3338 | 11.4 | 21.6258 | 12.7338 | 0 | 21.6258 |  |  |  |
| Saskatchewan | Atorvastatin 40mg daily | 2295296 | 90 | 90 |  | 0.2342 | 21.078 | 2.1078 | 11.4 | 34.5858 | 13.5078 | 9.5858 | 25 |  | Saskatchewan /Annual costs |  |
| Saskatchewan | Omeprazole 20mg daily | 2245058 | 90 | 90 |  | 0.2287 | 20.583 | 2.0583 | 11.4 | 34.0413 | 13.4583 | 9.0413 | 25 |  | Pharmacy Margin | 532.4918348 |
| Saskatchewan | Irbesartan/HCTZ 300 mg/25 mg daily | 2447894 | 90 | 90 |  | 0.2184 | 19.656 | 1.9656 | 11.4 | 33.0216 | 13.3656 | 8.0216 | 25 |  | Government Share | 106.6331 |
| Saskatchewan | Levothyroxine 50 mcg daily | 2213192 | 90 | 90 |  | 0.0316 | 2.844 | 0.8532 | 11.4 | 15.0972 | 12.2532 | 0 | 15.0972 |  | Patient Share | 4083.920934 |
| Saskatchewan | Atenolol 50mg daily | 2255545 | 90 | 90 |  | 0.1107 | 9.963 | 1.49445 | 11.4 | 22.85745 | 12.89445 | 0 | 22.85745 |  |  |  |
| Saskatchewan | Liraglutide inj 1.8mg daily | 2351064 | 90 | 27 |  | 29.7367 | 802.8909 | 20 | 11.4 | 834.2909 | 31.4 | 0 | 834.2909 |  |  |  |
| Saskatchewan | Lorazepam 1mg QHS | 655759 | 90 | 90 |  | 0.0447 | 4.023 | 1.2069 | 11.4 | 16.6299 | 12.6069 | 0 | 16.6299 |  |  |  |
| ***Q2*** | Lorazepam 1mg QHS |  |  | **45** |  | 0.0447 | 2.0115 | 0.60345 | 11.4 | 14.01495 | 12.00345 | 0 | 14.01495 |  |  |  |
| ***Q3*** | Lorazepam 1mg QHS |  |  | **30** |  | 0.0447 | 1.341 | 0.4023 | 11.4 | 13.1433 | 11.8023 | 0 | 13.1433 |  |  |  |
| ***Q4*** | Lorazepam 1mg QHS |  |  | **0** |  | 0.0447 | 0 | 0 | 0 | 0 | 0 | 0 | 0 |  |  |  |
| Saskatchewan | ASAEC 81mg daily | 2237726 | 90 | 90 |  | 0.1095 | 9.855 | 5.306538462 | 0 | 15.16153846 | 5.306538462 | 0 | 15.16153846 |  |  |  |
| Saskatchewan | Calcium 500mg / Vitamin D 1000U BID | 80017748 | 90 | 180 |  | 0.09389 | 16.9002 | 9.100107692 | 0 | 26.00030769 | 9.100107692 | 0 | 26.00030769 |  |  |  |
| Saskatchewan/ Total Q1 |  |  |  |  |  |  |  |  |  |  | 136.6266962 | 26.6487 | 1026.663096 |  |  |  |
| Northwest Territories | Metformin 1000mg BID | 2167786 | 90 | 360 |  | 0.0247 | 8.892 | 1.6325712 | 12.72 | 23.2445712 | 14.3525712 | 23.2445712 | 0 |  |  |  |
| Northwest Territories | Atorvastatin 40mg daily | 2295296 | 90 | 90 |  | 0.2342 | 21.078 | 3.8699208 | 12.72 | 37.6679208 | 16.5899208 | 37.6679208 | 0 |  | Northwest Territories /Annual costs |  |
| Northwest Territories | Omeprazole 20mg daily | 2245058 | 90 | 90 |  | 0.2287 | 20.583 | 3.7790388 | 12.72 | 37.0820388 | 16.4990388 | 37.0820388 | 0 |  | Pharmacy Margin | 714.2280194 |
| Northwest Territories | Irbesartan/HCTZ 300 mg/25 mg daily | 2447894 | 90 | 90 |  | 0.2184 | 19.656 | 3.6088416 | 12.72 | 35.9848416 | 16.3288416 | 35.9848416 | 0 |  | Government share | 744.9449346 |
| Northwest Territories | Levothyroxine 50 mcg daily | 2213192 | 90 | 90 |  | 0.031 | 2.79 | 0.512244 | 12.72 | 16.022244 | 13.232244 | 16.022244 | 0 |  | Patient share | 3627.090985 |
| Northwest Territories | Atenolol 50mg daily | 2255545 | 90 | 90 |  | 0.1107 | 9.963 | 1.8292068 | 12.72 | 24.5122068 | 14.5492068 | 24.5122068 | 0 |  |  |  |
| Northwest Territories | Liraglutide inj 1.8mg daily | 2351064 | 90 | 27 |  | 29.7367 | 802.8909 | 50 | 12.72 | 865.6109 | 62.72 | 0 | 865.6109 |  |  |  |
| Northwest Territories | Lorazepam 1mg QHS | 655759 | 90 | 90 |  | 0.0447 | 4.023 | 0.7386228 | 12.72 | 17.4816228 | 13.4586228 | 17.4816228 | 0 |  |  |  |
| ***Q2*** | Lorazepam 1mg QHS |  |  | **45** |  | 0.0447 | 2.0115 | 0.3693114 | 12.72 | 15.1008114 | 13.0893114 | 15.1008114 | 0 |  |  |  |
| ***Q3*** | Lorazepam 1mg QHS |  |  | **30** |  | 0.0447 | 1.341 | 0.2462076 | 12.72 | 14.3072076 | 12.9662076 | 14.3072076 | 0 |  |  |  |
| ***Q4*** | Lorazepam 1mg QHS |  |  | **0** |  | 0.0447 | 0 | 0 | 0 | 0 | 0 | 0 | 0 |  |  |  |
| Northwest Territories | ASAEC 81mg daily | 2237726 | 90 | 90 |  | 0.1095 | 9.855 | 5.306538462 | 0 | 15.16153846 | 5.306538462 | 0 | 15.16153846 |  |  |  |
| Northwest Territories | Calcium 500mg / Vitamin D 1000U BID | 80017748 | 90 | 180 |  | 0.09389 | 16.9002 | 9.100107692 | 0 | 26.00030769 | 9.100107692 | 0 | 26.00030769 |  |  |  |
| Northwest Territories |  |  |  |  |  |  |  |  |  |  | 182.1370922 | 191.995446 | 906.7727462 |  |  |  |
| Nunavut | Metformin 1000mg BID | 2167786 | 90 | 360 |  | 0.0247 | 8.892 | 2.1127392 | 16.95 | 27.9547392 | 19.0627392 | 27.9547392 | 0 |  |  |  |
| Nunavut | Atorvastatin 40mg daily | 2295296 | 90 | 90 |  | 0.2342 | 21.078 | 5.0081328 | 16.95 | 43.0361328 | 21.9581328 | 43.0361328 | 0 |  | Nunavut /Annual costs |  |
| Nunavut | Omeprazole 20mg daily | 2245058 | 90 | 90 |  | 0.2287 | 20.583 | 4.8905208 | 16.95 | 42.4235208 | 21.8405208 | 42.4235208 | 0 |  | Pharmacy Margin | 863.6760884 |
| Nunavut | Irbesartan/HCTZ 300 mg/25 mg daily | 2447894 | 90 | 90 |  | 0.2184 | 19.656 | 4.6702656 | 16.95 | 41.2762656 | 21.6202656 | 41.2762656 | 0 |  | Government share | 877.4730036 |
| Nunavut | Levothyroxine 50 mcg daily | 2213192 | 90 | 90 |  | 0.031 | 2.79 | 0.662904 | 16.95 | 20.402904 | 17.612904 | 20.402904 | 0 |  | Patient share | 3644.010985 |
| Nunavut | Atenolol 50mg daily | 2255545 | 90 | 90 |  | 0.1107 | 9.963 | 2.3672088 | 16.95 | 29.2802088 | 19.3172088 | 29.2802088 | 0 |  |  |  |
| Nunavut | Liraglutide inj 1.8mg daily | 2351064 | 90 | 27 |  | 29.7367 | 802.8909 | 50 | 16.95 | 869.8409 | 66.95 | 0 | 869.8409 |  |  |  |
| Nunavut | Lorazepam 1mg QHS | 655759 | 90 | 90 |  | 0.0447 | 4.023 | 0.9558648 | 16.95 | 21.9288648 | 17.9058648 | 21.9288648 | 0 |  |  |  |
| ***Q2*** | Lorazepam 1mg QHS |  |  | **45** |  | 0.0447 | 2.0115 | 0.4779324 | 16.95 | 19.4394324 | 17.4279324 | 19.4394324 | 0 |  |  |  |
| ***Q3*** | Lorazepam 1mg QHS |  |  | **30** |  | 0.0447 | 1.341 | 0.3186216 | 16.95 | 18.6096216 | 17.2686216 | 18.6096216 | 0 |  |  |  |
| ***Q4*** | Lorazepam 1mg QHS |  |  | **0** |  | 0.0447 | 0 | 0 | 0 | 0 | 0 | 0 | 0 |  |  |  |
| Nunavut | ASAEC 81mg daily | 2237726 | 90 | 90 |  | 0.1095 | 9.855 | 5.306538462 | 0 | 15.16153846 | 5.306538462 | 0 | 15.16153846 |  |  |  |
| Nunavut | Calcium 500mg / Vitamin D 1000U BID | 80017748 | 90 | 180 |  | 0.09389 | 16.9002 | 9.100107692 | 0 | 26.00030769 | 9.100107692 | 0 | 26.00030769 |  |  |  |
| Nunavut |  |  |  |  |  |  |  |  |  |  | 220.6742822 | 226.302636 | 911.0027462 |  |  |  |
| Yukon | Metformin 1000mg BID | 2167786 | 90 | 360 |  | 0.0247 | 8.892 | 2.1127392 | 12.72 | 23.7247392 | 14.8327392 | 23.7247392 | 0 |  |  |  |
| Yukon | Atorvastatin 40mg daily | 2295296 | 90 | 90 |  | 0.2342 | 21.078 | 5.0081328 | 12.72 | 38.8061328 | 17.7281328 | 38.8061328 | 0 |  | Yukon /Annual costs |  |
| Yukon | Omeprazole 20mg daily | 2245058 | 90 | 90 |  | 0.2287 | 20.583 | 4.8905208 | 12.72 | 38.1935208 | 17.6105208 | 38.1935208 | 0 |  | Pharmacy Margin | 768.7730196 |
| Yukon | Irbesartan/HCTZ 300 mg/25 mg daily | 2357410 | 90 | 90 |  | 0.2184 | 19.656 | 4.6702656 | 12.72 | 37.0462656 | 17.3902656 | 37.0462656 | 0 |  | Government Share | 848.3605888 |
| Yukon | Levothyroxine 50 mcg daily | 2213192 | 90 | 90 |  | 0.03 | 2.7 | 0.64152 | 12.72 | 16.06152 | 13.36152 | 16.06152 | 0 |  | Patient Share | 3566.444831 |
| Yukon | Atenolol 50mg daily | 2255545 | 90 | 90 |  | 0.1107 | 9.963 | 2.3672088 | 12.72 | 25.0502088 | 15.0872088 | 25.0502088 | 0 |  |  |  |
| Yukon | Liraglutide inj 1.8mg daily | 2351064 | 90 | 27 |  | 29.7367 | 802.8909 | 50 | 12.72 | 865.6109 | 62.72 | 0 | 865.6109 |  |  |  |
| Yukon | Lorazepam 1mg QHS | 655759 | 90 | 90 |  | 0.04 | 3.6 | 0.85536 | 12.72 | 17.17536 | 13.57536 | 17.17536 | 0 |  |  |  |
| ***Q2*** | Lorazepam 1mg QHS |  |  | **45** |  | 0.04 | 1.8 | 0.42768 | 12.72 | 14.94768 | 13.14768 | 14.94768 | 0 |  |  |  |
| ***Q3*** | Lorazepam 1mg QHS |  |  | **30** |  | 0.04 | 1.2 | 0.28512 | 12.72 | 14.20512 | 13.00512 | 14.20512 | 0 |  |  |  |
| ***Q4*** | Lorazepam 1mg QHS |  |  | **0** |  | 0.04 | 0 | 0 | 0 | 0 | 0 | 0 | 0 |  |  |  |
| Yukon | ASAEC 81mg daily | 2237726 | 90 | 90 |  | 0.08 | 7.2 | 1.71072 | 12.72 | 21.63072 | 14.43072 | 21.63072 | 0 |  |  |  |
| Yukon | Calcium 500mg / Vitamin D 1000U BID | 80017748 | 90 | 180 |  | 0.09389 | 16.9002 | 9.100107692 | 0 | 26.00030769 | 9.100107692 | 0 | 26.00030769 |  |  |  |
| Yukon |  |  |  |  |  |  |  |  |  |  | 195.8365749 | 217.6884672 | 891.6112077 |  |  |  |

Supplementary Table 5 – Scenario 4

| Province | Drug | DIN | Days Supply | Quantity | MAC/Unit | Drug Cost / Unit | Drug Cost | Markup | Dispensing Fee | Total Cost | Pharmacy Margin $ | Government Share | Patient Share |  |  |  |
| --- | --- | --- | --- | --- | --- | --- | --- | --- | --- | --- | --- | --- | --- | --- | --- | --- |
| Alberta | Metformin 1000mg BID | 2167786 | 90 | 360 |  | 0.0247 | 8.892 | 0.9078732 | 12.15 | 21.9498732 | 13.0578732 | 15.36491124 | 6.58496196 |  |  |  |
| Alberta | Atorvastatin 40mg daily | 2295296 | 90 | 90 |  | 0.2342 | 21.078 | 2.1520638 | 12.15 | 35.3800638 | 14.3020638 | 24.76604466 | 10.61401914 |  | Alberta/ Annual costs |  |
| Alberta | Omeprazole 20mg daily | 2245058 | 90 | 90 |  | 0.2287 | 20.583 | 2.1015243 | 12.15 | 34.8345243 | 14.2515243 | 24.38416701 | 10.45035729 |  | Pharmacy Margin | 934.4988608 |
| **Q2** | Omeprazole 10 mg daily | 2230737 |  | 90 | 0.0669 | 1.894 | 6.021 | 0.6147441 | 12.15 | 18.7857441 | 12.7647441 | 13.15002087 | 5.63572323 |  | Government Share | 446.5627916 |
| **Q3** |  |  |  | 0 |  | 0 | 0 | 0 | 0 | 0 | 0 | 0 | 0 |  | Patient Share | 4098.732468 |
| **Q4** |  |  |  | 0 |  | 0 | 0 | 0 | 0 | 0 | 0 | 0 | 0 |  |  |  |
| Alberta | Irbesartan/HCTZ 300 mg/25 mg daily | 2447894 | 90 | 90 |  | 0.2184 | 19.656 | 2.0068776 | 12.15 | 33.8128776 | 14.1568776 | 23.66901432 | 10.14386328 |  |  |  |
| Alberta | Levothyroxine 50 mcg daily | 2213192 | 90 | 90 |  | 0.031 | 2.79 | 0.284859 | 12.15 | 15.224859 | 12.434859 | 10.6574013 | 4.5674577 |  |  |  |
| Alberta | Atenolol 50mg daily | 2255545 | 90 | 90 |  | 0.1107 | 9.963 | 1.0172223 | 12.15 | 23.1302223 | 13.1672223 | 16.19115561 | 6.93906669 |  |  |  |
| Alberta | Liraglutide inj 1.8mg daily | 2351064 | 90 | 27 |  | 29.7367 | 802.8909 | 120.6343577 | 12.15 | 935.6752577 | 132.7843577 | 0 | 935.6752577 |  |  |  |
| Alberta | Lorazepam 1mg QHS | 655759 | 90 | 90 |  | 0.0447 | 4.023 | 0.4107483 | 12.15 | 16.5837483 | 12.5607483 | 11.60862381 | 4.97512449 |  |  |  |
| Alberta | ASAEC 81mg daily | 2237726 | 90 | 90 |  | 0.1095 | 9.855 | 5.306538462 | 0 | 15.16153846 | 5.306538462 | 0 | 15.16153846 |  |  |  |
| Alberta | Calcium 500mg / Vitamin D 1000U BID | 80017748 | 90 | 180 |  | 0.09389 | 16.9002 | 9.100107692 | 0 | 26.00030769 | 9.100107692 | 0 | 26.00030769 |  |  |  |
| Alberta/ Total Q1 |  |  |  |  |  |  |  |  |  |  | 241.1221724 | 126.641318 | 1031.111954 |  |  |  |
| British Columbia | Metformin 1000mg BID | 2167786 | 90 | 360 | 0.0267 |  | 8.9 | 0.712 | 10 | 19.612 | 10.712 | 0 | 19.612 |  |  |  |
| British Columbia | Atorvastatin 40mg daily | 2295296 | 90 | 90 | 0.2529 |  | 21.075 | 1.686 | 10 | 32.761 | 11.686 | 0 | 32.761 |  | British Columbia/ Annual costs |  |
| British Columbia | Omeprazole 20mg daily | 2245058 | 90 | 90 | 0.2025 |  | 16.875 | 1.35 | 10 | 28.225 | 11.35 | 0 | 28.225 |  | Pharmacy Margin | 672.2915187 |
| **Q2** | Omeprazole 10 mg daily | 2230737 |  | 90 |  | 1.894 | 170.46 | 12.62666667 | 10 | 180.46 | 22.62666667 | 0 | 180.46 |  | Government Share | 0 |
| **Q3** |  |  |  | 0 | 0 |  | 0 | 0 | 0 | 0 | 0 | 0 | 0 |  | Patient Share | 4438.53092 |
| **Q4** |  |  |  | 0 | 0 |  | 0 | 0 | 0 | 0 | 0 | 0 | 0 |  |  |  |
| British Columbia | Irbesartan/HCTZ 300 mg/25 mg daily | 2447894 | 90 | 90 | 0.2719 |  | 22.65833 | 1.812666667 | 10 | 34.471 | 11.81266667 | 0 | 34.471 |  |  |  |
| British Columbia | Levothyroxine 50 mcg daily | 2213192 | 90 | 90 | 0.0341 |  | 2.841667 | 0.227333333 | 10 | 13.069 | 10.22733333 | 0 | 13.069 |  |  |  |
| British Columbia | Atenolol 50mg daily | 2255545 | 90 | 90 | 0.1196 |  | 9.966667 | 0.797333333 | 10 | 20.764 | 10.79733333 | 0 | 20.764 |  |  |  |
| British Columbia | Liraglutide inj 1.8mg daily | 2351064 | 90 | 27 |  | 29.7367 | 802.8909 | 64.231272 | 10 | 877.122172 | 74.231272 | 0 | 877.122172 |  |  |  |
| British Columbia | Lorazepam 1mg QHS | 655759 | 90 | 90 | 0.0483 |  | 4.025 | 0.322 | 10 | 14.347 | 10.322 | 0 | 14.347 |  |  |  |
| British Columbia | ASAEC 81mg daily | 2237726 | 90 | 90 | 0.1035 |  | 8.625 | 0.69 | 10 | 19.315 | 10.69 | 0 | 19.315 |  |  |  |
| British Columbia | Calcium 500mg / Vitamin D 1000U BID | 80017748 | 90 | 180 | 0 | 0.09389 | 16.9002 | 9.100107692 | 0 | 26.00030769 | 9.100107692 | 0 | 26.00030769 |  |  |  |
| British Columbia/ Total Q1 |  |  |  |  |  |  |  |  |  |  | 170.928713 | 0 | 1085.68648 |  |  |  |
| Manitoba | Metformin 1000mg BID | 2167786 | 90 | 360 |  | 0.0259 | 9.324 | 0 | 13.65 | 22.974 | 13.65 | 22.974 | 0 |  |  |  |
| Manitoba | Atorvastatin 40mg daily | 2295296 | 90 | 90 |  | 0.2459 | 22.131 | 0 | 13.65 | 35.781 | 13.65 | 35.781 | 0 |  |  |  |
| Manitoba | Omeprazole 20mg daily | 2245058 | 90 | 90 |  | 0.2401 | 21.609 | 0 | 13.65 | 35.259 | 13.65 | 35.259 | 0 |  | Manitoba/ Annual costs |  |
| **Q2** | Omeprazole 10 mg daily | 2230737 | 90 | 90 |  | 1.894 | 170.46 | 0 | 13.65 | 184.11 | 13.65 | 0 | 184.11 |  | Pharmacy Margin | 467.1265848 |
| **Q3** |  |  |  | 0 |  | 0 | 0 | 0 | 0 | 0 | 0 | 0 | 0 |  | Government Share | 641.931 |
| **Q4** |  |  |  | 0 |  | 0 | 0 | 0 | 0 | 0 | 0 | 0 | 0 |  | Patient Share | 3614.920985 |
| Manitoba | Irbesartan/HCTZ 300 mg/25 mg daily | 2447894 | 90 | 90 |  | 0.2293 | 20.637 | 0 | 13.65 | 34.287 | 13.65 | 34.287 | 0 |  |  |  |
| Manitoba | Levothyroxine 50 mcg daily | 2213192 | 90 | 90 |  | 0.031 | 2.79 | 0 | 13.65 | 16.44 | 13.65 | 16.44 | 0 |  |  |  |
| Manitoba | Atenolol 50mg daily | 2255545 | 90 | 90 |  | 0.1162 | 10.458 | 0 | 13.65 | 24.108 | 13.65 | 24.108 | 0 |  |  |  |
| Manitoba | Liraglutide inj 1.8mg daily | 2351064 | 90 | 27 |  | 29.7367 | 802.8909 | 0 | 13.65 | 816.5409 | 13.65 | 0 | 816.5409 |  |  |  |
| Manitoba | Lorazepam 1mg QHS | 655759 | 90 | 90 |  | 0.0492 | 4.428 | 0 | 13.65 | 18.078 | 13.65 | 18.078 | 0 |  |  |  |
| Manitoba | ASAEC 81mg daily | 2237726 | 90 | 90 |  | 0.1095 | 9.855 | 5.306538462 | 0 | 15.16153846 | 5.306538462 | 0 | 15.16153846 |  |  |  |
| Manitoba | Calcium 500mg / Vitamin D 1000U BID | 80017748 | 90 | 180 |  | 0.09389 | 16.9002 | 9.100107692 | 0 | 26.00030769 | 9.100107692 | 0 | 26.00030769 |  |  |  |
| Manitoba/ Total Q1 |  |  |  |  |  |  |  |  |  |  | 123.6066462 | 186.927 | 857.7027462 |  |  |  |
| New Brunswick | Metformin 1000mg BID | 2167786 | 90 | 360 | 0.0247 |  | 8.892 | 0.71136 | 11 | 20.60336 | 11.71136 | 14.422352 | 6.181008 |  |  |  |
| New Brunswick | Atorvastatin 40mg daily | 2295296 | 90 | 90 | 0.2342 |  | 21.078 | 1.68624 | 11 | 33.76424 | 12.68624 | 23.634968 | 10.129272 |  |  |  |
| New Brunswick | Omeprazole 20mg daily | 2245058 | 90 | 90 | 0.2287 |  | 20.583 | 1.64664 | 11 | 33.22964 | 12.64664 | 23.260748 | 9.968892 |  | New Brunswick/ Annual costs |  |
| **Q2** | Omeprazole 10 mg daily | 2230737 | 90 | 90 |  | 1.894 | 170.46 | 13.6368 | 11 | 195.0968 | 24.6368 | 0 | 195.0968 |  | Pharmacy Margin | 680.1909528 |
| **Q3** |  |  |  | 0 | 0 | 0 | 0 | 0 | 0 | 0 | 0 | 0 | 0 |  | Government Share | 408.484636 |
| **Q4** |  |  |  | 0 | 0 | 0 | 0 | 0 | 0 | 0 | 0 | 0 | 0 |  | Patient Share | 4047.297717 |
| New Brunswick | Irbesartan/HCTZ 300 mg/25 mg daily | 2447894 | 90 | 90 | 0.2184 |  | 19.656 | 1.57248 | 11 | 32.22848 | 12.57248 | 22.559936 | 9.668544 |  |  |  |
| New Brunswick | Levothyroxine 50 mcg daily | 2213192 | 90 | 90 |  | 0.0311 | 2.879 | 0 | 11 | 13.879 | 11 | 9.7153 | 4.1637 |  |  |  |
| New Brunswick | Atenolol 50mg daily | 2255545 | 90 | 90 | 0.1107 |  | 9.963 | 0.79704 | 11 | 21.76004 | 11.79704 | 15.232028 | 6.528012 |  |  |  |
| New Brunswick | Liraglutide inj 1.8mg daily | 2351064 | 90 | 27 | 0 | 29.7367 | 802.8909 | 64.231272 | 11 | 878.122172 | 75.231272 | 0 | 878.122172 |  |  |  |
| New Brunswick | Lorazepam 1mg QHS | 655759 | 90 | 90 | 0.0447 |  | 4.023 | 0.32184 | 11 | 15.34484 | 11.32184 | 10.741388 | 4.603452 |  |  |  |
| New Brunswick | ASAEC 81mg daily | 2237726 | 90 | 90 |  | 0.1095 | 9.855 | 5.306538462 | 0 | 15.16153846 | 5.306538462 | 0 | 15.16153846 |  |  |  |
| New Brunswick | Calcium 500mg / Vitamin D 1000U BID | 80017748 | 90 | 180 |  | 0.09389 | 16.9002 | 9.100107692 | 0 | 26.00030769 | 9.100107692 | 0 | 26.00030769 |  |  |  |
| New Brunswick/ Total Q1 |  |  |  |  |  |  |  |  |  |  | 173.3735182 | 119.56672 | 970.5268982 |  |  |  |
| Newfoundland and Labrador | Metformin 1000mg BID | 2167786 | 90 | 360 |  | 0.0269 | 8.925346 | 0.758654378 | 12 | 21.684 | 12.75865438 | 15.684 | 6 |  |  |  |
| Newfoundland and Labrador | Atorvastatin 40mg daily | 2295296 | 90 | 90 |  | 0.2553 | 21.17696 | 1.800041475 | 12 | 34.977 | 13.80004147 | 28.977 | 6 |  |  |  |
| Newfoundland and Labrador | Omeprazole 20mg daily | 2245058 | 90 | 90 |  | 0.2493 | 20.67926 | 1.757737327 | 12 | 34.437 | 13.75773733 | 28.437 | 6 |  | Newfoundland and Labrador/ Annual costs |  |
| **Q2** | Omeprazole 10 mg daily | 2230737 |  | 90 |  | 2.0645 | 171.2488 | 14.55615207 | 12 | 197.805 | 26.55615207 | 191.805 | 6 |  | Pharmacy Margin | 857.6618617 |
| **Q3** |  |  |  | 0 |  | 0 | 0 | 0 | 0 | 0 | 0 | 0 | 0 |  | Government Share | 653.754 |
| **Q4** |  |  |  | 0 |  | 0 | 0 | 0 | 0 | 0 | 0 | 0 | 0 |  | Patient Share | 3981.251709 |
| Newfoundland and Labrador | Irbesartan/HCTZ 300 mg/25 mg daily | 2447894 | 90 | 90 |  | 0.2381 | 19.75023 | 1.678769585 | 12 | 33.429 | 13.67876959 | 27.429 | 6 |  |  |  |
| Newfoundland and Labrador | Levothyroxine 50 mcg daily | 2213192 | 90 | 90 |  | 0.0338 | 2.803687 | 0.238313364 | 12 | 15.042 | 12.23831336 | 9.042 | 6 |  |  |  |
| Newfoundland and Labrador | Atenolol 50mg daily | 2255545 | 90 | 90 |  | 0.1207 | 10.01198 | 0.851018433 | 12 | 22.863 | 12.85101843 | 16.863 | 6 |  |  |  |
| Newfoundland and Labrador | Liraglutide inj 1.8mg daily | 2351064 | 90 | 27 |  | 29.7367 | 802.8909 | 72.260181 | 40 | 915.151081 | 112.260181 | 0 | 915.151081 |  |  |  |
| Newfoundland and Labrador | Lorazepam 1mg QHS | 655759 | 90 | 90 |  | 0.0487 | 4.039631 | 0.343368664 | 12 | 16.383 | 12.34336866 | 10.383 | 6 |  |  |  |
| Newfoundland and Labrador | ASAEC 81mg daily | 2237726 | 90 | 90 |  | 0.1095 | 9.855 | 5.306538462 | 0 | 15.16153846 | 5.306538462 | 0 | 15.16153846 |  |  |  |
| Newfoundland and Labrador | Calcium 500mg / Vitamin D 1000U BID | 80017748 | 90 | 180 |  | 0.09389 | 16.9002 | 9.100107692 | 0 | 26.00030769 | 9.100107692 | 0 | 26.00030769 |  |  |  |
| Newfoundland and Labrador/ Total Q1 |  |  |  |  |  |  |  |  |  |  | 218.0947304 | 136.815 | 998.3129272 |  |  |  |
| Nova Scotia | Metformin 1000mg BID | 2167786 | 90 | 360 | 0.0247 |  | 8.892 | 0.71136 | 11.95 | 21.55336 | 12.66136 | 15.087352 | 6.466008 |  |  |  |
| Nova Scotia | Atorvastatin 40mg daily | 2295296 | 90 | 90 | 0.2342 |  | 21.078 | 1.68624 | 11.95 | 34.71424 | 13.63624 | 24.299968 | 10.414272 |  |  |  |
| Nova Scotia | Omeprazole 20mg daily | 2245058 | 90 | 90 | 0.2287 |  | 20.583 | 1.64664 | 11.95 | 34.17964 | 13.59664 | 23.925748 | 10.253892 |  | Nova Scotia/ Annual costs |  |
| **Q2** | Omeprazole 10 mg daily | 2230737 |  | 90 | 0.2059 |  | 18.531 | 1.48248 | 11.95 | 31.96348 | 13.43248 | 22.374436 | 9.589044 |  | Pharmacy Margin | 778.0202028 |
| **Q3** |  |  |  | 0 | 0 |  | 0 | 0 | 0 | 0 | 0 | 0 | 0 |  | Government Share | 448.222208 |
| **Q4** |  |  |  | 0 | 0 |  | 0 | 0 | 0 | 0 | 0 | 0 | 0 |  | Patient Share | 3953.320395 |
| Nova Scotia | Irbesartan/HCTZ 300 mg/25 mg daily | 2447894 | 90 | 90 | 0.2184 |  | 19.656 | 1.57248 | 11.95 | 33.17848 | 13.52248 | 23.224936 | 9.953544 |  |  |  |
| Nova Scotia | Levothyroxine 50 mcg daily | 2213192 | 90 | 90 | 0 | 0.0316 | 2.844 | 0.29862 | 11.95 | 15.09262 | 12.24862 | 10.564834 | 4.527786 |  |  |  |
| Nova Scotia | Atenolol 50mg daily | 2255545 | 90 | 90 | 0.1107 | 0 | 9.963 | 0.79704 | 11.95 | 22.71004 | 12.74704 | 15.897028 | 6.813012 |  |  |  |
| Nova Scotia | Liraglutide inj 1.8mg daily | 2351064 | 90 | 27 | 0 | 29.7367 | 802.8909 | 84.3035445 | 11.95 | 899.1444445 | 96.2535445 | 0 | 899.1444445 |  |  |  |
| Nova Scotia | Lorazepam 1mg QHS | 655759 | 90 | 90 | 0.0447 | 0 | 4.023 | 0.32184 | 11.95 | 16.29484 | 12.27184 | 11.406388 | 4.888452 |  |  |  |
| Nova Scotia | ASAEC 81mg daily | 2237726 | 90 | 90 | 0 | 0.1095 | 9.855 | 5.306538462 | 0 | 15.16153846 | 5.306538462 | 0 | 15.16153846 |  |  |  |
| Nova Scotia | Calcium 500mg / Vitamin D 1000U BID | 80017748 | 90 | 180 | 0 | 0.09389 | 16.9002 | 9.100107692 | 0 | 26.00030769 | 9.100107692 | 0 | 26.00030769 |  |  |  |
| Nova Scotia/ Total Q1 |  |  |  |  |  |  |  |  |  |  | 201.3444107 | 124.406254 | 993.6232567 |  |  |  |
| Ontario | Metformin 1000mg BID | 2167786 | 90 | 360 | 0 | 0.0247 | 8.892 | 0.71136 | 8.83 | 18.43336 | 9.54136 | 12.32336 | 6.11 |  |  |  |
| Ontario | Atorvastatin 40mg daily | 2295296 | 90 | 90 | 0 | 0.2342 | 21.078 | 1.68624 | 8.83 | 31.59424 | 10.51624 | 25.48424 | 6.11 |  |  |  |
| Ontario | Omeprazole 20mg daily | 2245058 | 90 | 90 | 0 | 0.2287 | 20.583 | 1.64664 | 8.83 | 31.05964 | 10.47664 | 24.94964 | 6.11 |  | Ontario/ Annual costs |  |
| **Q2** | Omeprazole 10 mg daily | 2230737 | 90 | 90 |  | 1.894 | 170.46 | 13.6368 | 8.83 | 192.9268 | 22.4668 | 0 | 192.9268 |  | Pharmacy Margin | 616.0010328 |
| **Q3** |  |  |  | 0 | 0 | 0 | 0 | 0 | 0 | 0 | 0 | 0 | 0 |  | Government Share | 377.31956 |
| **Q4** |  |  |  | 0 | 0 | 0 | 0 | 0 | 0 | 0 | 0 | 0 | 0 |  | Patient Share | 4014.132873 |
| Ontario | Irbesartan/HCTZ 300 mg/25 mg daily | 2447894 | 90 | 90 | 0 | 0.2184 | 19.656 | 1.57248 | 8.83 | 30.05848 | 10.40248 | 23.94848 | 6.11 |  |  |  |
| Ontario | Levothyroxine 50 mcg daily | 2213192 | 90 | 90 | 0 | 0.0316 | 2.844 | 0.22752 | 8.83 | 11.90152 | 9.05752 | 5.79152 | 6.11 |  |  |  |
| Ontario | Atenolol 50mg daily | 2255545 | 90 | 90 | 0 | 0.1107 | 9.963 | 0.79704 | 8.83 | 19.59004 | 9.62704 | 13.48004 | 6.11 |  |  |  |
| Ontario | Liraglutide inj 1.8mg daily | 2351064 | 90 | 27 | 0 | 29.7367 | 802.8909 | 64.231272 | 8.83 | 875.952172 | 73.061272 | 0 | 875.952172 |  |  |  |
| Ontario | Lorazepam 1mg QHS | 655759 | 90 | 90 | 0 | 0.0447 | 4.023 | 0.32184 | 8.83 | 13.17484 | 9.15184 | 7.06484 | 6.11 |  |  |  |
| Ontario | ASAEC 81mg daily | 2237726 | 90 | 90 | 0 | 0.1095 | 9.855 | 5.306538462 | 0 | 15.16153846 | 5.306538462 | 0 | 15.16153846 |  |  |  |
| Ontario | Calcium 500mg / Vitamin D 1000U BID | 80017748 | 90 | 180 | 0 | 0.09389 | 16.9002 | 9.100107692 | 0 | 26.00030769 | 9.100107692 | 0 | 26.00030769 |  |  |  |
| Ontario/ Total Q1 |  |  |  |  |  |  |  |  |  |  | 156.2410382 | 113.04212 | 959.8840182 |  |  |  |
| Prince Edward Island | Metformin 1000mg BID | 2167786 | 90 | 360 | 0.0247 |  | 8.892 | 0.53352 | 12.36 | 21.78552 | 12.89352 | 5.84552 | 15.94 |  |  |  |
| Prince Edward Island | Atorvastatin 40mg daily | 2295296 | 90 | 90 | 0.2342 |  | 21.078 | 1.26468 | 12.36 | 34.70268 | 13.62468 | 18.76268 | 15.94 |  |  |  |
| Prince Edward Island | Omeprazole 20mg daily | 2245058 | 90 | 90 | 0.2287 |  | 20.583 | 1.23498 | 12.36 | 34.17798 | 13.59498 | 18.23798 | 15.94 |  | Prince Edward Island/ Annual costs |  |
| **Q2** | Omeprazole 10 mg daily | 2230737 |  | 90 |  | 1.894 | 170.46 | 10.2276 | 12.36 | 193.0476 | 22.5876 | 0 | 193.0476 |  | Pharmacy Margin | 648.5194608 |
| **Q3** |  |  |  | 0 | 0 | 0 | 0 | 0 | 0 | 0 | 0 | 0 | 0 |  | Government Share | 252.61046 |
| **Q4** |  |  |  | 0 | 0 | 0 | 0 | 0 | 0 | 0 | 0 | 0 | 0 |  | Patient Share | 4171.1444 |
| Prince Edward Island | Irbesartan/HCTZ 300 mg/25 mg daily | 2447894 | 90 | 90 | 0.2184 |  | 19.656 | 1.17936 | 12.36 | 33.19536 | 13.53936 | 17.25536 | 15.94 |  |  |  |
| Prince Edward Island | Levothyroxine 50 mcg daily | 2213192 | 90 | 90 |  | 0.031 | 2.79 | 0.1674 | 12.36 | 15.3174 | 12.5274 | 4.8374 | 10.48 |  |  |  |
| Prince Edward Island | Atenolol 50mg daily | 2255545 | 90 | 90 | 0.1107 |  | 9.963 | 0.59778 | 12.36 | 22.92078 | 12.95778 | 6.98078 | 15.94 |  |  |  |
| Prince Edward Island | Liraglutide inj 1.8mg daily | 2351064 | 90 | 27 |  | 29.7367 | 802.8909 | 48.173454 | 12.36 | 863.424354 | 60.533454 | 0 | 863.424354 |  |  |  |
| Prince Edward Island | Lorazepam 1mg QHS | 655759 | 90 | 90 | 0.0447 |  | 4.023 | 0.24138 | 12.36 | 16.62438 | 12.60138 | 4.91138 | 11.713 |  |  |  |
| Prince Edward Island | ASAEC 81mg daily | 2237726 | 90 | 90 |  | 0.1095 | 9.855 | 5.306538462 | 0 | 15.16153846 | 5.306538462 | 0 | 15.16153846 |  |  |  |
| Prince Edward Island | Calcium 500mg / Vitamin D 1000U BID | 80017748 | 90 | 180 |  | 0.09389 | 16.9002 | 9.100107692 | 0 | 26.00030769 | 9.100107692 | 0 | 26.00030769 |  |  |  |
| Prince Edward Island/ Total Q1 |  |  |  |  |  |  |  |  |  |  | 166.6792002 | 76.8311 | 1006.4792 |  |  |  |
| Quebec | Metformin 1000mg BID | 2167786 | 90 | 360 |  | 0.0247 | 8.892 | 0.57798 | 27 | 36.46998 | 27.57798 | 23.74195698 | 12.72802302 |  |  |  |
| Quebec | Atorvastatin 40mg daily | 2295296 | 90 | 90 |  | 0.2342 | 21.078 | 1.37007 | 27 | 49.44807 | 28.37007 | 32.19069357 | 17.25737643 |  |  |  |
| Quebec | Omeprazole 20mg daily | 2245058 | 90 | 90 |  | 0.2287 | 20.583 | 1.337895 | 27 | 48.920895 | 28.337895 | 31.84750265 | 17.07339236 |  | Quebec/ Annual costs |  |
| **Q2** | Omeprazole 10 mg daily | 2230737 |  | 90 |  | 1.894 | 170.46 | 11.0799 | 27 | 208.5399 | 38.0799 | 0 | 208.5399 |  | Pharmacy Margin | 1057.49126 |
| **Q3** |  |  |  | 0 |  | 0 | 0 | 0 | 0 | 0 | 0 | 0 | 0 |  | Government Share | 2416.720174 |
| **Q4** |  |  |  | 0 |  | 0 | 0 | 0 | 0 | 0 | 0 | 0 | 0 |  | Patient Share | 1695.786886 |
| Quebec | Irbesartan/HCTZ 300 mg/25 mg daily | 2447894 | 90 | 90 |  | 0.2184 | 19.656 | 1.27764 | 27 | 47.93364 | 28.27764 | 31.20479964 | 16.72884036 |  |  |  |
| Quebec | Levothyroxine 50 mcg daily | 2213192 | 90 | 90 |  | 0.0274 | 2.466 | 0.16029 | 27 | 29.62629 | 27.16029 | 19.28671479 | 10.33957521 |  |  |  |
| Quebec | Atenolol 50mg daily | 2255545 | 90 | 90 |  | 0.1107 | 9.963 | 0.647595 | 27 | 37.610595 | 27.647595 | 24.48449735 | 13.12609766 |  |  |  |
| Quebec | Liraglutide inj 1.8mg daily | 2351064 | 90 | 27 |  | 22.83 | 616.41 | 40.06665 | 27 | 683.47665 | 67.06665 | 444.9432992 | 238.5333509 |  |  |  |
| Quebec | Lorazepam 1mg QHS | 655759 | 90 | 90 |  | 0.0447 | 4.023 | 0.261495 | 27 | 31.284495 | 27.261495 | 20.36620625 | 10.91828876 |  |  |  |
| Quebec | ASAEC 81mg daily | 2237726 | 90 | 90 |  | 0.1095 | 9.855 | 5.306538462 | 0 | 15.16153846 | 5.306538462 | 0 | 15.16153846 |  |  |  |
| Quebec | Calcium 500mg / Vitamin D 1000U BID | 80017748 | 90 | 180 |  | 0.09389 | 16.9002 | 9.100107692 | 0 | 26.00030769 | 9.100107692 | 0 | 26.00030769 |  |  |  |
| Quebec/Total Q1 |  |  |  |  |  |  |  |  |  |  | 276.1062612 | 628.0656704 | 377.8667908 |  |  |  |
| Saskatchewan | Metformin 1000mg BID | 2167786 | 90 | 360 |  | 0.0247 | 8.892 | 1.3338 | 11.4 | 21.6258 | 12.7338 | 0 | 21.6258 |  |  |  |
| Saskatchewan | Atorvastatin 40mg daily | 2295296 | 90 | 90 |  | 0.2342 | 21.078 | 2.1078 | 11.4 | 34.5858 | 13.5078 | 9.5858 | 25 |  |  |  |
| Saskatchewan | Omeprazole 20mg daily | 2245058 | 90 | 90 |  | 0.2287 | 20.583 | 2.0583 | 11.4 | 34.0413 | 13.4583 | 9.0413 | 25 |  | Saskatchewan/ Annual costs |  |
| **Q2** | Omeprazole 10 mg daily | 2230737 |  | 90 |  | 1.894 | 170.46 | 17.046 | 11.4 | 198.906 | 28.446 | 173.906 | 25 |  | Pharmacy Margin | 534.5778848 |
| **Q3** |  |  |  | 0 | 0 | 0 | 0 | 0 | 0 | 0 | 0 | 0 | 0 |  | Government Share | 253.3769 |
| **Q4** |  |  |  | 0 | 0 | 0 | 0 | 0 | 0 | 0 | 0 | 0 | 0 |  | Patient Share | 4056.652384 |
| Saskatchewan | Irbesartan/HCTZ 300 mg/25 mg daily | 2447894 | 90 | 90 |  | 0.2184 | 19.656 | 1.9656 | 11.4 | 33.0216 | 13.3656 | 8.0216 | 25 |  |  |  |
| Saskatchewan | Levothyroxine 50 mcg daily | 2213192 | 90 | 90 |  | 0.0316 | 2.844 | 0.8532 | 11.4 | 15.0972 | 12.2532 | 0 | 15.0972 |  |  |  |
| Saskatchewan | Atenolol 50mg daily | 2255545 | 90 | 90 |  | 0.1107 | 9.963 | 1.49445 | 11.4 | 22.85745 | 12.89445 | 0 | 22.85745 |  |  |  |
| Saskatchewan | Liraglutide inj 1.8mg daily | 2351064 | 90 | 27 |  | 29.7367 | 802.8909 | 20 | 11.4 | 834.2909 | 31.4 | 0 | 834.2909 |  |  |  |
| Saskatchewan | Lorazepam 1mg QHS | 655759 | 90 | 90 |  | 0.0447 | 4.023 | 1.2069 | 11.4 | 16.6299 | 12.6069 | 0 | 16.6299 |  |  |  |
| Saskatchewan | ASAEC 81mg daily | 2237726 | 90 | 90 |  | 0.1095 | 9.855 | 5.306538462 | 0 | 15.16153846 | 5.306538462 | 0 | 15.16153846 |  |  |  |
| Saskatchewan | Calcium 500mg / Vitamin D 1000U BID | 80017748 | 90 | 180 |  | 0.09389 | 16.9002 | 9.100107692 | 0 | 26.00030769 | 9.100107692 | 0 | 26.00030769 |  |  |  |
| Saskatchewan/ Total Q1 |  |  |  |  |  |  |  |  |  |  | 136.6266962 | 26.6487 | 1026.663096 |  |  |  |
| Northwest Territories | Metformin 1000mg BID | 2167786 | 90 | 360 |  | 0.0247 | 8.892 | 1.6325712 | 12.72 | 23.2445712 | 14.3525712 | 23.2445712 | 0 |  |  |  |
| Northwest Territories | Atorvastatin 40mg daily | 2295296 | 90 | 90 |  | 0.2342 | 21.078 | 3.8699208 | 12.72 | 37.6679208 | 16.5899208 | 37.6679208 | 0 |  |  |  |
| Northwest Territories | Omeprazole 20mg daily | 2245058 | 90 | 90 |  | 0.2287 | 20.583 | 3.7790388 | 12.72 | 37.0820388 | 16.4990388 | 37.0820388 | 0 |  | Northwest Territories/Annual costs |  |
| **Q2** | Omeprazole 10 mg daily |  |  | 90 | 0.0669 | 1.894 | 6.021 | 1.1054556 | 12.72 | 19.8464556 | 13.8254556 | 19.8464556 | 0 |  | Pharmacy Margin | 692.876708 |
| **Q3** |  |  |  | 0 |  | 0 | 0 | 0 | 0 | 0 | 0 | 0 | 0 |  | Government Share | 676.5821228 |
| **Q4** |  |  |  | 0 |  | 0 | 0 | 0 | 0 | 0 | 0 | 0 | 0 |  | Patient Share | 3627.090985 |
| Northwest Territories | Irbesartan/HCTZ 300 mg/25 mg daily | 2447894 | 90 | 90 |  | 0.2184 | 19.656 | 3.6088416 | 12.72 | 35.9848416 | 16.3288416 | 35.9848416 | 0 |  |  |  |
| Northwest Territories | Levothyroxine 50 mcg daily | 2213192 | 90 | 90 |  | 0.031 | 2.79 | 0.512244 | 12.72 | 16.022244 | 13.232244 | 16.022244 | 0 |  |  |  |
| Northwest Territories | Atenolol 50mg daily | 2255545 | 90 | 90 |  | 0.1107 | 9.963 | 1.8292068 | 12.72 | 24.5122068 | 14.5492068 | 24.5122068 | 0 |  |  |  |
| Northwest Territories | Liraglutide inj 1.8mg daily | 2351064 | 90 | 27 |  | 29.7367 | 802.8909 | 50 | 12.72 | 865.6109 | 62.72 | 0 | 865.6109 |  |  |  |
| Northwest Territories | Lorazepam 1mg QHS | 655759 | 90 | 90 |  | 0.0447 | 4.023 | 0.7386228 | 12.72 | 17.4816228 | 13.4586228 | 17.4816228 | 0 |  |  |  |
| Northwest Territories | ASAEC 81mg daily | 2237726 | 90 | 90 |  | 0.1095 | 9.855 | 5.306538462 | 0 | 15.16153846 | 5.306538462 | 0 | 15.16153846 |  |  |  |
| Northwest Territories | Calcium 500mg / Vitamin D 1000U BID | 80017748 | 90 | 180 |  | 0.09389 | 16.9002 | 9.100107692 | 0 | 26.00030769 | 9.100107692 | 0 | 26.00030769 |  |  |  |
| Northwest Territories |  |  |  |  |  |  |  |  |  |  | 182.1370922 | 191.995446 | 906.7727462 |  |  |  |
| Nunavut | Metformin 1000mg BID | 2167786 | 90 | 360 |  | 0.0247 | 8.892 | 2.1127392 | 16.95 | 27.9547392 | 19.0627392 | 27.9547392 | 0 |  |  |  |
| Nunavut | Atorvastatin 40mg daily | 2295296 | 90 | 90 |  | 0.2342 | 21.078 | 5.0081328 | 16.95 | 43.0361328 | 21.9581328 | 43.0361328 | 0 |  |  |  |
| Nunavut | Omeprazole 20mg daily | 2245058 | 90 | 90 |  | 0.2287 | 20.583 | 4.8905208 | 16.95 | 42.4235208 | 21.8405208 | 42.4235208 | 0 |  | Nunavut/ Annual costs |  |
| **Q2** | Omeprazole 10 mg daily |  |  | 90 | 0.0669 | 1.894 | 6.021 | 1.4305896 | 16.95 | 24.4015896 | 18.3805896 | 24.4015896 | 0 |  | Pharmacy Margin | 835.556156 |
| **Q3** |  |  |  | 0 |  | 0 | 0 | 0 | 0 | 0 | 0 | 0 | 0 |  | Government Share | 802.3415712 |
| **Q4** |  |  |  | 0 |  | 0 | 0 | 0 | 0 | 0 | 0 | 0 | 0 |  | Patient Share | 3644.010985 |
| Nunavut | Irbesartan/HCTZ 300 mg/25 mg daily | 2447894 | 90 | 90 |  | 0.2184 | 19.656 | 4.6702656 | 16.95 | 41.2762656 | 21.6202656 | 41.2762656 | 0 |  |  |  |
| Nunavut | Levothyroxine 50 mcg daily | 2213192 | 90 | 90 |  | 0.031 | 2.79 | 0.662904 | 16.95 | 20.402904 | 17.612904 | 20.402904 | 0 |  |  |  |
| Nunavut | Atenolol 50mg daily | 2255545 | 90 | 90 |  | 0.1107 | 9.963 | 2.3672088 | 16.95 | 29.2802088 | 19.3172088 | 29.2802088 | 0 |  |  |  |
| Nunavut | Liraglutide inj 1.8mg daily | 2351064 | 90 | 27 |  | 29.7367 | 802.8909 | 50 | 16.95 | 869.8409 | 66.95 | 0 | 869.8409 |  |  |  |
| Nunavut | Lorazepam 1mg QHS | 655759 | 90 | 90 |  | 0.0447 | 4.023 | 0.9558648 | 16.95 | 21.9288648 | 17.9058648 | 21.9288648 | 0 |  |  |  |
| Nunavut | ASAEC 81mg daily | 2237726 | 90 | 90 |  | 0.1095 | 9.855 | 5.306538462 | 0 | 15.16153846 | 5.306538462 | 0 | 15.16153846 |  |  |  |
| Nunavut | Calcium 500mg / Vitamin D 1000U BID | 80017748 | 90 | 180 |  | 0.09389 | 16.9002 | 9.100107692 | 0 | 26.00030769 | 9.100107692 | 0 | 26.00030769 |  |  |  |
| Nunavut |  |  |  |  |  |  |  |  |  |  | 220.6742822 | 226.302636 | 911.0027462 |  |  |  |
| Yukon | Metformin 1000mg BID | 2167786 | 90 | 360 |  | 0.0247 | 8.892 | 2.1127392 | 12.72 | 23.7247392 | 14.8327392 | 23.7247392 | 0 |  |  |  |
| Yukon | Atorvastatin 40mg daily | 2295296 | 90 | 90 |  | 0.2342 | 21.078 | 5.0081328 | 12.72 | 38.8061328 | 17.7281328 | 38.8061328 | 0 |  |  |  |
| Yukon | Omeprazole 20mg daily | 2245058 | 90 | 90 |  | 0.2287 | 20.583 | 4.8905208 | 12.72 | 38.1935208 | 17.6105208 | 38.1935208 | 0 |  | Yukon/ Annual costs |  |
| **Q2** | Omeprazole 10 mg daily | 2230737 |  | 90 | 0.82 |  | 73.8 | 1.29888 | 12.72 | 87.81888 | 14.01888 | 87.81888 | 0 |  | Pharmacy Margin | 760.7696172 |
| **Q3** |  |  |  | 0 | 0 | 0 | 0 | 0 | 0 | 0 | 0 | 0 | 0 |  | Government Share | 860.1592941 |
| **Q4** |  |  |  | 0 | 0 | 0 | 0 | 0 | 0 | 0 | 0 | 0 | 0 |  | Patient Share | 3566.444831 |
| Yukon | Irbesartan/HCTZ 300 mg/25 mg daily | 2357410 | 90 | 90 |  | 0.2184 | 19.656 | 4.6702656 | 12.72 | 37.0462656 | 17.3902656 | 37.0462656 | 0 |  |  |  |
| Yukon | Levothyroxine 50 mcg daily | 2213192 | 90 | 90 |  | 0.03 | 2.7 | 0.64152 | 12.72 | 16.06152 | 13.36152 | 16.06152 | 0 |  |  |  |
| Yukon | Atenolol 50mg daily | 2255545 | 90 | 90 |  | 0.1107 | 9.963 | 2.3672088 | 12.72 | 25.0502088 | 15.0872088 | 25.0502088 | 0 |  |  |  |
| Yukon | Liraglutide inj 1.8mg daily | 2351064 | 90 | 27 |  | 29.7367 | 802.8909 | 50 | 12.72 | 865.6109 | 62.72 | 0 | 865.6109 |  |  |  |
| Yukon | Lorazepam 1mg QHS | 655759 | 90 | 90 |  | 0.04 | 3.6 | 0.85536 | 12.72 | 17.17536 | 13.57536 | 17.17536 | 0 |  |  |  |
| Yukon | ASAEC 81mg daily | 2237726 | 90 | 90 |  | 0.08 | 7.2 | 1.71072 | 12.72 | 21.63072 | 14.43072 | 21.63072 | 0 |  |  |  |
| Yukon | Calcium 500mg / Vitamin D 1000U BID | 80017748 | 90 | 180 |  | 0.09389 | 16.9002 | 9.100107692 | 0 | 26.00030769 | 9.100107692 | 0 | 26.00030769 |  |  |  |
| Yukon |  |  |  |  |  |  |  |  |  |  | 195.8365749 | 217.6884672 | 891.6112077 |  |  |  |

Supplementary Table 6 – Scenario 5

| Province | Drug | DIN | Days Supply | Quantity | MAC/Unit | Drug Cost / Unit | Drug Cost | Markup | Dispensing Fee | Total Cost | Pharmacy Margin $ | Government Share | Patient Share |  |  |  |
| --- | --- | --- | --- | --- | --- | --- | --- | --- | --- | --- | --- | --- | --- | --- | --- | --- |
| Alberta | Metformin 1000mg BID | 2167786 | 90 | 360 |  | 0.0247 | 8.892 | 0.9078732 | 12.15 | 21.9498732 | 13.0578732 | 15.36491124 | 6.58496196 |  |  |  |
| Alberta | Atorvastatin 40mg daily | 2295296 | 90 | 90 |  | 0.2342 | 21.078 | 2.1520638 | 12.15 | 35.3800638 | 14.3020638 | 24.76604466 | 10.61401914 |  | Alberta /Annual costs |  |
| Alberta | Omeprazole 20mg daily | 2245058 | 90 | 90 |  | 0.2287 | 20.583 | 2.1015243 | 12.15 | 34.8345243 | 14.2515243 | 24.38416701 | 10.45035729 |  | Pharmacy Margin | 930.8776963 |
| Alberta | Irbesartan/HCTZ 300 mg/25 mg daily | 2447894 | 90 | 90 |  | 0.2184 | 19.656 | 2.0068776 | 12.15 | 33.8128776 | 14.1568776 | 23.66901432 | 10.14386328 |  | Government Share | 460.1307766 |
| Alberta | Levothyroxine 50 mcg daily | 2213192 | 90 | 90 |  | 0.031 | 2.79 | 0.284859 | 12.15 | 15.224859 | 12.434859 | 10.6574013 | 4.5674577 |  | Patient Share | 4152.06732 |
| Alberta | Atenolol 50mg daily | 2255545 | 90 | 90 |  | 0.1107 | 9.963 | 1.0172223 | 12.15 | 23.1302223 | 13.1672223 | 16.19115561 | 6.93906669 |  |  |  |
| Alberta | Liraglutide inj 1.8mg daily | 2351064 | 90 | 27 |  | 29.7367 | 802.8909 | 120.6343577 | 12.15 | 935.6752577 | 132.7843577 | 0 | 935.6752577 |  |  |  |
| Alberta | Melatonin 5 mg | 80021179 | 90 | 90 |  | 0.0858 | 7.722 | 4.158 | 0 | 11.88 | 4.158 | 0 | 11.88 |  |  |  |
| Alberta | ASAEC 81mg daily | 2237726 | 90 | 90 |  | 0.1095 | 9.855 | 5.306538462 | 0 | 15.16153846 | 5.306538462 | 0 | 15.16153846 |  |  |  |
| Alberta | Calcium 500mg / Vitamin D 1000U BID | 80017748 | 90 | 180 |  | 0.09389 | 16.9002 | 9.100107692 | 0 | 26.00030769 | 9.100107692 | 0 | 26.00030769 |  |  |  |
| Alberta/ Total Q1 |  |  |  |  |  |  |  |  |  |  | 232.7194241 | 115.0326941 | 1038.01683 |  |  |  |
| British Columbia | Metformin 1000mg BID | 2167786 | 90 | 360 | 0.0267 |  | 8.9 | 0.712 | 10 | 19.612 | 10.712 | 0 | 19.612 |  |  |  |
| British Columbia | Atorvastatin 40mg daily | 2295296 | 90 | 90 | 0.2529 |  | 21.075 | 1.686 | 10 | 32.761 | 11.686 | 0 | 32.761 |  | British Columbia /Annual costs |  |
| British Columbia | Omeprazole 20mg daily | 2245058 | 90 | 90 | 0.2025 |  | 16.875 | 1.35 | 10 | 28.225 | 11.35 | 0 | 28.225 |  | Pharmacy Margin | 659.0588521 |
| British Columbia | Irbesartan/HCTZ 300 mg/25 mg daily | 2447894 | 90 | 90 | 0.2719 |  | 22.65833 | 1.812666667 | 10 | 34.471 | 11.81266667 | 0 | 34.471 |  | Government Share | 0 |
| British Columbia | Levothyroxine 50 mcg daily | 2213192 | 90 | 90 | 0.0341 |  | 2.841667 | 0.227333333 | 10 | 13.069 | 10.22733333 | 0 | 13.069 |  | Patient Share | 4332.877919 |
| British Columbia | Atenolol 50mg daily | 2255545 | 90 | 90 | 0.1196 |  | 9.966667 | 0.797333333 | 10 | 20.764 | 10.79733333 | 0 | 20.764 |  |  |  |
| British Columbia | Liraglutide inj 1.8mg daily | 2351064 | 90 | 27 |  | 29.7367 | 802.8909 | 64.231272 | 10 | 877.122172 | 74.231272 | 0 | 877.122172 |  |  |  |
| British Columbia | Melatonin 5 mg | 80021179 | 90 | 90 |  | 0.0858 | 7.722 | 4.158 | 0 | 11.88 | 4.158 | 0 | 11.88 |  |  |  |
| British Columbia | ASAEC 81mg daily | 2237726 | 90 | 90 | 0.1035 |  | 8.625 | 0.69 | 10 | 19.315 | 10.69 | 0 | 19.315 |  |  |  |
| British Columbia | Calcium 500mg / Vitamin D 1000U BID | 80017748 | 90 | 180 |  | 0.09389 | 16.9002 | 9.100107692 | 0 | 26.00030769 | 9.100107692 | 0 | 26.00030769 |  |  |  |
| British Columbia/ Total Q1 |  |  |  |  |  |  |  |  |  |  | 164.764713 | 0 | 1083.21948 |  |  |  |
| Manitoba | Metformin 1000mg BID | 2167786 | 90 | 360 |  | 0.0259 | 9.324 | 0 | 13.65 | 22.974 | 13.65 | 22.974 | 0 |  |  |  |
| Manitoba | Atorvastatin 40mg daily | 2295296 | 90 | 90 |  | 0.2459 | 22.131 | 0 | 13.65 | 35.781 | 13.65 | 35.781 | 0 |  | Manitoba /Annual costs |  |
| Manitoba | Omeprazole 20mg daily | 2245058 | 90 | 90 |  | 0.2401 | 21.609 | 0 | 13.65 | 35.259 | 13.65 | 35.259 | 0 |  | Pharmacy Margin | 456.4585846 |
| Manitoba | Irbesartan/HCTZ 300 mg/25 mg daily | 2447894 | 90 | 90 |  | 0.2293 | 20.637 | 0 | 13.65 | 34.287 | 13.65 | 34.287 | 0 |  | Government Share | 675.396 |
| Manitoba | Levothyroxine 50 mcg daily | 2213192 | 90 | 90 |  | 0.031 | 2.79 | 0 | 13.65 | 16.44 | 13.65 | 16.44 | 0 |  | Patient Share | 3478.330985 |
| Manitoba | Atenolol 50mg daily | 2255545 | 90 | 90 |  | 0.1162 | 10.458 | 0 | 13.65 | 24.108 | 13.65 | 24.108 | 0 |  |  |  |
| Manitoba | Liraglutide inj 1.8mg daily | 2351064 | 90 | 27 |  | 29.7367 | 802.8909 | 0 | 13.65 | 816.5409 | 13.65 | 0 | 816.5409 |  |  |  |
| Manitoba | Melatonin 5 mg | 80021179 | 90 | 90 |  | 0.0858 | 7.722 | 4.158 | 0 | 11.88 | 4.158 | 0 | 11.88 |  |  |  |
| Manitoba | ASAEC 81mg daily | 2237726 | 90 | 90 |  | 0.1095 | 9.855 | 5.306538462 | 0 | 15.16153846 | 5.306538462 | 0 | 15.16153846 |  |  |  |
| Manitoba | Calcium 500mg / Vitamin D 1000U BID | 80017748 | 90 | 180 |  | 0.09389 | 16.9002 | 9.100107692 | 0 | 26.00030769 | 9.100107692 | 0 | 26.00030769 |  |  |  |
| Manitoba/ Total Q1 |  |  |  |  |  |  |  |  |  |  | 114.1146462 | 168.849 | 869.5827462 |  |  |  |
| New Brunswick | Metformin 1000mg BID | 2167786 | 90 | 360 | 0.0247 |  | 8.892 | 0.71136 | 11 | 20.60336 | 11.71136 | 14.422352 | 6.181008 |  |  |  |
| New Brunswick | Atorvastatin 40mg daily | 2295296 | 90 | 90 | 0.2342 |  | 21.078 | 1.68624 | 11 | 33.76424 | 12.68624 | 23.634968 | 10.129272 |  | New Brunswick /Annual costs |  |
| New Brunswick | Omeprazole 20mg daily | 2245058 | 90 | 90 | 0.2287 |  | 20.583 | 1.64664 | 11 | 33.22964 | 12.64664 | 23.260748 | 9.968892 |  | Pharmacy Margin | 664.8387126 |
| New Brunswick | Irbesartan/HCTZ 300 mg/25 mg daily | 2447894 | 90 | 90 | 0.2184 |  | 19.656 | 1.57248 | 11 | 32.22848 | 12.57248 | 22.559936 | 9.668544 |  | Government Share | 435.301328 |
| New Brunswick | Levothyroxine 50 mcg daily | 2213192 | 90 | 90 |  | 0.0311 | 2.879 | 0 | 11 | 13.879 | 11 | 9.7153 | 4.1637 |  | Patient Share | 3911.213785 |
| New Brunswick | Atenolol 50mg daily | 2255545 | 90 | 90 | 0.1107 |  | 9.963 | 0.79704 | 11 | 21.76004 | 11.79704 | 15.232028 | 6.528012 |  |  |  |
| New Brunswick | Liraglutide inj 1.8mg daily | 2351064 | 90 | 27 |  | 29.7367 | 802.8909 | 64.231272 | 11 | 878.122172 | 75.231272 | 0 | 878.122172 |  |  |  |
| New Brunswick | Melatonin 5 mg | 80021179 | 90 | 90 |  | 0.0858 | 7.722 | 4.158 | 0 | 11.88 | 4.158 | 0 | 11.88 |  |  |  |
| New Brunswick | ASAEC 81mg daily | 2237726 | 90 | 90 |  | 0.1095 | 9.855 | 5.306538462 | 0 | 15.16153846 | 5.306538462 | 0 | 15.16153846 |  |  |  |
| New Brunswick | Calcium 500mg / Vitamin D 1000U BID | 80017748 | 90 | 180 |  | 0.09389 | 16.9002 | 9.100107692 | 0 | 26.00030769 | 9.100107692 | 0 | 26.00030769 |  |  |  |
| New Brunswick/ Total Q1 |  |  |  |  |  |  |  |  |  |  | 166.2096782 | 108.825332 | 977.8034462 |  |  |  |
| Newfoundland and Labrador | Metformin 1000mg BID | 2167786 | 90 | 360 |  | 0.0269 | 8.925346 | 0.758654378 | 12 | 21.684 | 12.75865438 | 15.684 | 6 |  |  |  |
| Newfoundland and Labrador | Atorvastatin 40mg daily | 2295296 | 90 | 90 |  | 0.2553 | 21.17696 | 1.800041475 | 12 | 34.977 | 13.80004147 | 28.977 | 6 |  | Newfoundland and Labrador / Annual costs |  |
| Newfoundland and Labrador | Omeprazole 20mg daily | 2245058 | 90 | 90 |  | 0.2493 | 20.67926 | 1.757737327 | 12 | 34.437 | 13.75773733 | 28.437 | 6 |  | Pharmacy Margin | 839.6374469 |
| Newfoundland and Labrador | Irbesartan/HCTZ 300 mg/25 mg daily | 2447894 | 90 | 90 |  | 0.2381 | 19.75023 | 1.678769585 | 12 | 33.429 | 13.67876959 | 27.429 | 6 |  | Government Share | 505.728 |
| Newfoundland and Labrador | Levothyroxine 50 mcg daily | 2213192 | 90 | 90 |  | 0.0338 | 2.803687 | 0.238313364 | 12 | 15.042 | 12.23831336 | 9.042 | 6 |  | Patient Share | 4016.771709 |
| Newfoundland and Labrador | Atenolol 50mg daily | 2255545 | 90 | 90 |  | 0.1207 | 10.01198 | 0.851018433 | 12 | 22.863 | 12.85101843 | 16.863 | 6 |  |  |  |
| Newfoundland and Labrador | Liraglutide inj 1.8mg daily | 2351064 | 90 | 27 |  | 29.7367 | 802.8909 | 72.260181 | 40 | 915.151081 | 112.260181 | 0 | 915.151081 |  |  |  |
| Newfoundland and Labrador | Melatonin 5 mg | 80021179 | 90 | 90 |  | 0.0858 | 7.722 | 4.158 | 0 | 11.88 | 4.158 | 0 | 11.88 |  |  |  |
| Newfoundland and Labrador | ASAEC 81mg daily | 2237726 | 90 | 90 |  | 0.1095 | 9.855 | 5.306538462 | 0 | 15.16153846 | 5.306538462 | 0 | 15.16153846 |  |  |  |
| Newfoundland and Labrador | Calcium 500mg / Vitamin D 1000U BID | 80017748 | 90 | 180 |  | 0.09389 | 16.9002 | 9.100107692 | 0 | 26.00030769 | 9.100107692 | 0 | 26.00030769 |  |  |  |
| Newfoundland and Labrador/ Total Q1 |  |  |  |  |  |  |  |  |  |  | 209.9093617 | 126.432 | 1004.192927 |  |  |  |
| Nova Scotia | Metformin 1000mg BID | 2167786 | 90 | 360 | 0.0247 |  | 8.892 | 0.71136 | 11.95 | 21.55336 | 12.66136 | 15.087352 | 6.466008 |  |  |  |
| Nova Scotia | Atorvastatin 40mg daily | 2295296 | 90 | 90 | 0.2342 |  | 21.078 | 1.68624 | 11.95 | 34.71424 | 13.63624 | 24.299968 | 10.414272 |  | Nova Scotia /Annual costs |  |
| Nova Scotia | Omeprazole 20mg daily | 2245058 | 90 | 90 | 0.2287 |  | 20.583 | 1.64664 | 11.95 | 34.17964 | 13.59664 | 23.925748 | 10.253892 |  | Pharmacy Margin | 772.9222826 |
| Nova Scotia | Irbesartan/HCTZ 300 mg/25 mg daily | 2447894 | 90 | 90 | 0.2184 |  | 19.656 | 1.57248 | 11.95 | 33.17848 | 13.52248 | 23.224936 | 9.953544 |  | Government Share | 451.999464 |
| Nova Scotia | Levothyroxine 50 mcg daily | 2213192 | 90 | 90 |  |  | 2.844 | 0.29862 | 11.95 | 15.09262 | 12.24862 | 10.564834 | 4.527786 |  | Patient Share | 4002.459219 |
| Nova Scotia | Atenolol 50mg daily | 2255545 | 90 | 90 | 0.1107 |  | 9.963 | 0.79704 | 11.95 | 22.71004 | 12.74704 | 15.897028 | 6.813012 |  |  |  |
| Nova Scotia | Liraglutide inj 1.8mg daily | 2351064 | 90 | 27 |  | 29.7367 | 802.8909 | 84.3035445 | 11.95 | 899.1444445 | 96.2535445 | 0 | 899.1444445 |  |  |  |
| Nova Scotia | Melatonin 5 mg | 80021179 | 90 | 90 |  | 0.0858 | 7.722 | 4.158 | 0 | 11.88 | 4.158 | 0 | 11.88 |  |  |  |
| Nova Scotia | ASAEC 81mg daily | 2237726 | 90 | 90 |  | 0.1095 | 9.855 | 5.306538462 | 0 | 15.16153846 | 5.306538462 | 0 | 15.16153846 |  |  |  |
| Nova Scotia | Calcium 500mg / Vitamin D 1000U BID | 80017748 | 90 | 180 |  | 0.09389 | 16.9002 | 9.100107692 | 0 | 26.00030769 | 9.100107692 | 0 | 26.00030769 |  |  |  |
| Nova Scotia/ Total Q1 |  |  |  |  |  |  |  |  |  |  | 193.2305707 | 112.999866 | 1000.614805 |  |  |  |
| Ontario | Metformin 1000mg BID | 2167786 | 90 | 360 |  | 0.0247 | 8.892 | 0.71136 | 8.83 | 18.43336 | 9.54136 | 12.32336 | 6.11 |  |  |  |
| Ontario | Atorvastatin 40mg daily | 2295296 | 90 | 90 |  | 0.2342 | 21.078 | 1.68624 | 8.83 | 31.59424 | 10.51624 | 25.48424 | 6.11 |  | Ontario /Annual costs |  |
| Ontario | Omeprazole 20mg daily | 2245058 | 90 | 90 |  | 0.2287 | 20.583 | 1.64664 | 8.83 | 31.05964 | 10.47664 | 24.94964 | 6.11 |  | Pharmacy Margin | 604.9887926 |
| Ontario | Irbesartan/HCTZ 300 mg/25 mg daily | 2447894 | 90 | 90 |  | 0.2184 | 19.656 | 1.57248 | 8.83 | 30.05848 | 10.40248 | 23.94848 | 6.11 |  | Government Share | 423.90912 |
| Ontario | Levothyroxine 50 mcg daily | 2213192 | 90 | 90 |  | 0.0316 | 2.844 | 0.22752 | 8.83 | 11.90152 | 9.05752 | 5.79152 | 6.11 |  | Patient Share | 3862.616073 |
| Ontario | Atenolol 50mg daily | 2255545 | 90 | 90 |  | 0.1107 | 9.963 | 0.79704 | 8.83 | 19.59004 | 9.62704 | 13.48004 | 6.11 |  |  |  |
| Ontario | Liraglutide inj 1.8mg daily | 2351064 | 90 | 27 |  | 29.7367 | 802.8909 | 64.231272 | 8.83 | 875.952172 | 73.061272 | 0 | 875.952172 |  |  |  |
| Ontario | Melatonin 5 mg | 80021179 | 90 | 90 |  | 0.0858 | 7.722 | 4.158 | 0 | 11.88 | 4.158 | 0 | 11.88 |  |  |  |
| Ontario | ASAEC 81mg daily | 2237726 | 90 | 90 |  | 0.1095 | 9.855 | 5.306538462 | 0 | 15.16153846 | 5.306538462 | 0 | 15.16153846 |  |  |  |
| Ontario | Calcium 500mg / Vitamin D 1000U BID | 80017748 | 90 | 180 |  | 0.09389 | 16.9002 | 9.100107692 | 0 | 26.00030769 | 9.100107692 | 0 | 26.00030769 |  |  |  |
| Ontario/ Total Q1 |  |  |  |  |  |  |  |  |  |  | 151.2471982 | 105.97728 | 965.6540182 |  |  |  |
| Prince Edward Island | Metformin 1000mg BID | 2167786 | 90 | 360 | 0.0247 |  | 8.892 | 0.53352 | 12.36 | 21.78552 | 12.89352 | 5.84552 | 15.94 |  |  |  |
| Prince Edward Island | Atorvastatin 40mg daily | 2295296 | 90 | 90 | 0.2342 |  | 21.078 | 1.26468 | 12.36 | 34.70268 | 13.62468 | 18.76268 | 15.94 |  | Prince Edward Island / Annual costs |  |
| Prince Edward Island | Omeprazole 20mg daily | 2245058 | 90 | 90 | 0.2287 |  | 20.583 | 1.23498 | 12.36 | 34.17798 | 13.59498 | 18.23798 | 15.94 |  | Pharmacy Margin | 632.9432806 |
| Prince Edward Island | Irbesartan/HCTZ 300 mg/25 mg daily | 2447894 | 90 | 90 | 0.2184 |  | 19.656 | 1.17936 | 12.36 | 33.19536 | 13.53936 | 17.25536 | 15.94 |  | Government Share | 287.67888 |
| Prince Edward Island | Levothyroxine 50 mcg daily | 2213192 | 90 | 90 |  | 0.031 | 2.79 | 0.1674 | 12.36 | 15.3174 | 12.5274 | 4.8374 | 10.48 |  | Patient Share | 4026.584801 |
| Prince Edward Island | Atenolol 50mg daily | 2255545 | 90 | 90 | 0.1107 |  | 9.963 | 0.59778 | 12.36 | 22.92078 | 12.95778 | 6.98078 | 15.94 |  |  |  |
| Prince Edward Island | Liraglutide inj 1.8mg daily | 2351064 | 90 | 27 |  | 29.7367 | 802.8909 | 48.173454 | 12.36 | 863.424354 | 60.533454 | 0 | 863.424354 |  |  |  |
| Prince Edward Island | Melatonin 5 mg | 80021179 | 90 | 90 |  | 0.0858 | 7.722 | 4.158 | 0 | 11.88 | 4.158 | 0 | 11.88 |  |  |  |
| Prince Edward Island | ASAEC 81mg daily | 2237726 | 90 | 90 |  | 0.1095 | 9.855 | 5.306538462 | 0 | 15.16153846 | 5.306538462 | 0 | 15.16153846 |  |  |  |
| Prince Edward Island | Calcium 500mg / Vitamin D 1000U BID | 80017748 | 90 | 180 |  | 0.09389 | 16.9002 | 9.100107692 | 0 | 26.00030769 | 9.100107692 | 0 | 26.00030769 |  |  |  |
| Prince Edward Island/ Total Q1 | 0 |  |  |  |  |  |  |  |  |  | 158.2358202 | 71.91972 | 1006.6462 |  |  |  |
| Quebec | Metformin 1000mg BID | 2167786 | 90 | 360 |  | 0.0247 | 8.892 | 0.57798 | 27 | 36.46998 | 27.57798 | 23.74195698 | 12.72802302 |  |  |  |
| Quebec | Atorvastatin 40mg daily | 2295296 | 90 | 90 |  | 0.2342 | 21.078 | 1.37007 | 27 | 49.44807 | 28.37007 | 32.19069357 | 17.25737643 |  | Quebec /Annual costs |  |
| Quebec | Omeprazole 20mg daily | 2245058 | 90 | 90 |  | 0.2287 | 20.583 | 1.337895 | 27 | 48.920895 | 28.337895 | 31.84750265 | 17.07339236 |  | Pharmacy Margin | 1012.011065 |
| Quebec | Irbesartan/HCTZ 300 mg/25 mg daily | 2447894 | 90 | 90 |  | 0.2184 | 19.656 | 1.27764 | 27 | 47.93364 | 28.27764 | 31.20479964 | 16.72884036 |  | Government Share | 2430.797856 |
| Quebec | Levothyroxine 50 mcg daily | 2213192 | 90 | 90 |  | 0.0274 | 2.466 | 0.16029 | 27 | 29.62629 | 27.16029 | 19.28671479 | 10.33957521 |  | Patient Share | 1515.314008 |
| Quebec | Atenolol 50mg daily | 2255545 | 90 | 90 |  | 0.1107 | 9.963 | 0.647595 | 27 | 37.610595 | 27.647595 | 24.48449735 | 13.12609766 |  |  |  |
| Quebec | Liraglutide inj 1.8mg daily | 2351064 | 90 | 27 |  | 22.83 | 616.41 | 40.06665 | 27 | 683.47665 | 67.06665 | 444.9432992 | 238.5333509 |  |  |  |
| Quebec | Melatonin 5 mg | 80021179 | 90 | 90 |  | 0.0858 | 7.722 | 4.158 | 0 | 11.88 | 4.158 | 0 | 11.88 |  |  |  |
| Quebec | ASAEC 81mg daily | 2237726 | 90 | 90 |  | 0.1095 | 9.855 | 5.306538462 | 0 | 15.16153846 | 5.306538462 | 0 | 15.16153846 |  |  |  |
| Quebec | Calcium 500mg / Vitamin D 1000U BID | 80017748 | 90 | 180 |  | 0.09389 | 16.9002 | 9.100107692 | 0 | 26.00030769 | 9.100107692 | 0 | 26.00030769 |  |  |  |
| Quebec/Total Q1 |  |  |  |  |  |  |  |  |  |  | 253.0027662 | 607.6994641 | 378.828502 |  |  |  |
| Saskatchewan | Metformin 1000mg BID | 2167786 | 90 | 360 |  | 0.0247 | 8.892 | 1.3338 | 11.4 | 21.6258 | 12.7338 | 0 | 21.6258 |  |  |  |
| Saskatchewan | Atorvastatin 40mg daily | 2295296 | 90 | 90 |  | 0.2342 | 21.078 | 2.1078 | 11.4 | 34.5858 | 13.5078 | 9.5858 | 25 |  | Saskatchewan /Annual costs |  |
| Saskatchewan | Omeprazole 20mg daily | 2245058 | 90 | 90 |  | 0.2287 | 20.583 | 2.0583 | 11.4 | 34.0413 | 13.4583 | 9.0413 | 25 |  | Pharmacy Margin | 512.7111846 |
| Saskatchewan | Irbesartan/HCTZ 300 mg/25 mg daily | 2447894 | 90 | 90 |  | 0.2184 | 19.656 | 1.9656 | 11.4 | 33.0216 | 13.3656 | 8.0216 | 25 |  | Government Share | 106.5948 |
| Saskatchewan | Levothyroxine 50 mcg daily | 2213192 | 90 | 90 |  | 0.0316 | 2.844 | 0.8532 | 11.4 | 15.0972 | 12.2532 | 0 | 15.0972 |  | Patient Share | 4087.652785 |
| Saskatchewan | Atenolol 50mg daily | 2255545 | 90 | 90 |  | 0.1107 | 9.963 | 1.49445 | 11.4 | 22.85745 | 12.89445 | 0 | 22.85745 |  |  |  |
| Saskatchewan | Liraglutide inj 1.8mg daily | 2351064 | 90 | 27 |  | 29.7367 | 802.8909 | 20 | 11.4 | 834.2909 | 31.4 | 0 | 834.2909 |  |  |  |
| Saskatchewan | Melatonin 5 mg | 80021179 | 90 | 90 |  | 0.0858 | 7.722 | 4.158 | 0 | 11.88 | 4.158 | 0 | 11.88 |  |  |  |
| Saskatchewan | ASAEC 81mg daily | 2237726 | 90 | 90 |  | 0.1095 | 9.855 | 5.306538462 | 0 | 15.16153846 | 5.306538462 | 0 | 15.16153846 |  |  |  |
| Saskatchewan | Calcium 500mg / Vitamin D 1000U BID | 80017748 | 90 | 180 |  | 0.09389 | 16.9002 | 9.100107692 | 0 | 26.00030769 | 9.100107692 | 0 | 26.00030769 |  |  |  |
| Saskatchewan/ Total Q1 |  |  |  |  |  |  |  |  |  |  | 128.1777962 | 26.6487 | 1021.913196 |  |  |  |
| Northwest Territories | Metformin 1000mg BID | 2167786 | 90 | 360 |  | 0.0247 | 8.892 | 1.6325712 | 12.72 | 23.2445712 | 14.3525712 | 23.2445712 | 0 |  |  |  |
| Northwest Territories | Atorvastatin 40mg daily | 2295296 | 90 | 90 |  | 0.2342 | 21.078 | 3.8699208 | 12.72 | 37.6679208 | 16.5899208 | 37.6679208 | 0 |  | Northwest Territories /Annual costs |  |
| Northwest Territories | Omeprazole 20mg daily | 2245058 | 90 | 90 |  | 0.2287 | 20.583 | 3.7790388 | 12.72 | 37.0820388 | 16.4990388 | 37.0820388 | 0 |  | Pharmacy Margin | 691.3458774 |
| Northwest Territories | Irbesartan/HCTZ 300 mg/25 mg daily | 2447894 | 90 | 90 |  | 0.2184 | 19.656 | 3.6088416 | 12.72 | 35.9848416 | 16.3288416 | 35.9848416 | 0 |  | Government share | 698.0552928 |
| Northwest Territories | Levothyroxine 50 mcg daily | 2213192 | 90 | 90 |  | 0.031 | 2.79 | 0.512244 | 12.72 | 16.022244 | 13.232244 | 16.022244 | 0 |  | Patient share | 3674.610985 |
| Northwest Territories | Atenolol 50mg daily | 2255545 | 90 | 90 |  | 0.1107 | 9.963 | 1.8292068 | 12.72 | 24.5122068 | 14.5492068 | 24.5122068 | 0 |  |  |  |
| Northwest Territories | Liraglutide inj 1.8mg daily | 2351064 | 90 | 27 |  | 29.7367 | 802.8909 | 50 | 12.72 | 865.6109 | 62.72 | 0 | 865.6109 |  |  |  |
| Northwest Territories | Melatonin 5 mg | 80021179 | 90 | 90 |  | 0.0858 | 7.722 | 4.158 | 0 | 11.88 | 4.158 | 0 | 11.88 |  |  |  |
| Northwest Territories | ASAEC 81mg daily | 2237726 | 90 | 90 |  | 0.1095 | 9.855 | 5.306538462 | 0 | 15.16153846 | 5.306538462 | 0 | 15.16153846 |  |  |  |
| Northwest Territories | Calcium 500mg / Vitamin D 1000U BID | 80017748 | 90 | 180 |  | 0.09389 | 16.9002 | 9.100107692 | 0 | 26.00030769 | 9.100107692 | 0 | 26.00030769 |  |  |  |
| Northwest Territories |  |  |  |  |  |  |  |  |  |  | 172.8364694 | 174.5138232 | 918.6527462 |  |  |  |
| Nunavut | Metformin 1000mg BID | 2167786 | 90 | 360 | 0 | 0.0247 | 8.892 | 2.1127392 | 16.95 | 27.9547392 | 19.0627392 | 27.9547392 | 0 |  |  |  |
| Nunavut | Atorvastatin 40mg daily | 2295296 | 90 | 90 | 0 | 0.2342 | 21.078 | 5.0081328 | 16.95 | 43.0361328 | 21.9581328 | 43.0361328 | 0 |  | Nunavut /Annual costs |  |
| Nunavut | Omeprazole 20mg daily | 2245058 | 90 | 90 | 0 | 0.2287 | 20.583 | 4.8905208 | 16.95 | 42.4235208 | 21.8405208 | 42.4235208 | 0 |  | Pharmacy Margin | 827.7056694 |
| Nunavut | Irbesartan/HCTZ 300 mg/25 mg daily | 2447894 | 90 | 90 | 0 | 0.2184 | 19.656 | 4.6702656 | 16.95 | 41.2762656 | 21.6202656 | 41.2762656 | 0 |  | Government share | 817.4950848 |
| Nunavut | Levothyroxine 50 mcg daily | 2213192 | 90 | 90 | 0 | 0.031 | 2.79 | 0.662904 | 16.95 | 20.402904 | 17.612904 | 20.402904 | 0 |  | Patient share | 3691.530985 |
| Nunavut | Atenolol 50mg daily | 2255545 | 90 | 90 | 0 | 0.1107 | 9.963 | 2.3672088 | 16.95 | 29.2802088 | 19.3172088 | 29.2802088 | 0 |  |  |  |
| Nunavut | Liraglutide inj 1.8mg daily | 2351064 | 90 | 27 | 0 | 29.7367 | 802.8909 | 50 | 16.95 | 869.8409 | 66.95 | 0 | 869.8409 |  |  |  |
| Nunavut | Melatonin 5 mg | 80021179 | 90 | 90 |  | 0.0858 | 7.722 | 4.158 | 0 | 11.88 | 4.158 | 0 | 11.88 |  |  |  |
| Nunavut | ASAEC 81mg daily | 2237726 | 90 | 90 | 0 | 0.1095 | 9.855 | 5.306538462 | 0 | 15.16153846 | 5.306538462 | 0 | 15.16153846 |  |  |  |
| Nunavut | Calcium 500mg / Vitamin D 1000U BID | 80017748 | 90 | 180 | 0 | 0.09389 | 16.9002 | 9.100107692 | 0 | 26.00030769 | 9.100107692 | 0 | 26.00030769 |  |  |  |
| Nunavut |  |  |  |  |  |  |  |  |  |  | 206.9264174 | 204.3737712 | 922.8827462 |  |  |  |
| Yukon | Metformin 1000mg BID | 2167786 | 90 | 360 |  | 0.0247 | 8.892 | 2.1127392 | 12.72 | 23.7247392 | 14.8327392 | 23.7247392 | 0 |  |  |  |
| Yukon | Atorvastatin 40mg daily | 2295296 | 90 | 90 |  | 0.2342 | 21.078 | 5.0081328 | 12.72 | 38.8061328 | 17.7281328 | 38.8061328 | 0 |  | Yukon /Annual costs |  |
| Yukon | Omeprazole 20mg daily | 2245058 | 90 | 90 |  | 0.2287 | 20.583 | 4.8905208 | 12.72 | 38.1935208 | 17.6105208 | 38.1935208 | 0 |  | Pharmacy Margin | 745.6768596 |
| Yukon | Irbesartan/HCTZ 300 mg/25 mg daily | 2357410 | 90 | 90 |  | 0.2184 | 19.656 | 4.6702656 | 12.72 | 37.0462656 | 17.3902656 | 37.0462656 | 0 |  | Government Share | 802.0524288 |
| Yukon | Levothyroxine 50 mcg daily | 2213192 | 90 | 90 |  | 0.03 | 2.7 | 0.64152 | 12.72 | 16.06152 | 13.36152 | 16.06152 | 0 |  | Patient Share | 3613.964831 |
| Yukon | Atenolol 50mg daily | 2255545 | 90 | 90 |  | 0.1107 | 9.963 | 2.3672088 | 12.72 | 25.0502088 | 15.0872088 | 25.0502088 | 0 |  |  |  |
| Yukon | Liraglutide inj 1.8mg daily | 2351064 | 90 | 27 |  | 29.7367 | 802.8909 | 50 | 12.72 | 865.6109 | 62.72 | 0 | 865.6109 |  |  |  |
| Yukon | Melatonin 5 mg | 80021179 | 90 | 90 |  | 0.0858 | 7.722 | 4.158 | 0 | 11.88 | 4.158 | 0 | 11.88 |  |  |  |
| Yukon | ASAEC 81mg daily | 2237726 | 90 | 90 |  | 0.08 | 7.2 | 1.71072 | 12.72 | 21.63072 | 14.43072 | 21.63072 | 0 |  |  |  |
| Yukon | Calcium 500mg / Vitamin D 1000U BID | 80017748 | 90 | 180 |  | 0.09389 | 16.9002 | 9.100107692 | 0 | 26.00030769 | 9.100107692 | 0 | 26.00030769 |  |  |  |
| Yukon |  |  |  |  |  |  |  |  |  |  | 186.4192149 | 200.5131072 | 903.4912077 |  |  |  |

Supplementary Table 7 – Scenario 6

| Province | Drug | DIN | Days Supply | Quantity | MAC/Unit | Drug Cost / Unit | Drug Cost | Markup | Dispensing Fee | Total Cost | Pharmacy Margin $ | Government Share | Patient Share |  |  |  |
| --- | --- | --- | --- | --- | --- | --- | --- | --- | --- | --- | --- | --- | --- | --- | --- | --- |
| Alberta | Metformin 1000mg BID | 2167786 | 90 | 360 |  | 0.0247 | 8.892 | 0.9078732 | 12.15 | 21.9498732 | 13.0578732 | 15.36491124 | 6.58496196 |  |  |  |
| Alberta | Atorvastatin 40mg daily | 2295296 | 90 | 90 |  | 0.2342 | 21.078 | 2.1520638 | 12.15 | 35.3800638 | 14.3020638 | 24.76604466 | 10.61401914 |  | Alberta /Annual costs |  |
| Alberta | Omeprazole 20mg daily | 2245058 | 90 | 90 |  | 0.2287 | 20.583 | 2.1015243 | 12.15 | 34.8345243 | 14.2515243 | 24.38416701 | 10.45035729 |  | Pharmacy Margin | 964.1652367 |
| Alberta | Irbesartan/HCTZ 300 mg/25 mg daily | 2447894 | 90 | 90 |  | 0.2184 | 19.656 | 2.0068776 | 12.15 | 33.8128776 | 14.1568776 | 23.66901432 | 10.14386328 |  | Government Share | 504.1212548 |
| Alberta | Levothyroxine 50 mcg daily | 2213192 | 90 | 90 |  | 0.031 | 2.79 | 0.284859 | 12.15 | 15.224859 | 12.434859 | 10.6574013 | 4.5674577 |  | Patient Share | 4123.400382 |
| Alberta | Atenolol 50mg daily | 2255545 | 90 | 90 |  | 0.1107 | 9.963 | 1.0172223 | 12.15 | 23.1302223 | 13.1672223 | 16.19115561 | 6.93906669 |  |  |  |
| Alberta | Liraglutide inj 1.8mg daily | 2351064 | 90 | 27 |  | 29.7367 | 802.8909 | 120.6343577 | 12.15 | 935.6752577 | 132.7843577 | 0 | 935.6752577 |  |  |  |
| Alberta | Lorazepam .5 mg QHS | 655740 | 90 | 90 |  | 0.0359 | 3.231 | 0.3298851 | 12.15 | 15.7108851 | 12.4798851 | 10.99761957 | 4.71326553 |  |  |  |
| Alberta | ASAEC 81mg daily | 2237726 | 90 | 90 |  | 0.1095 | 9.855 | 5.306538462 | 0 | 15.16153846 | 5.306538462 | 0 | 15.16153846 |  |  |  |
| Alberta | Calcium 500mg / Vitamin D 1000U BID | 80017748 | 90 | 180 |  | 0.09389 | 16.9002 | 9.100107692 | 0 | 26.00030769 | 9.100107692 | 0 | 26.00030769 |  |  |  |
| Alberta/ Total Q1 |  |  |  |  |  |  |  |  |  |  | 241.0413092 | 126.0303137 | 1030.850095 |  |  |  |
| British Columbia | Metformin 1000mg BID | 2167786 | 90 | 360 | 0.0267 |  | 8.9 | 0.712 | 10 | 19.612 | 10.712 | 0 | 19.612 |  |  |  |
| British Columbia | Atorvastatin 40mg daily | 2295296 | 90 | 90 | 0.2529 |  | 21.075 | 1.686 | 10 | 32.761 | 11.686 | 0 | 32.761 |  | British Columbia /Annual costs |  |
| British Columbia | Omeprazole 20mg daily | 2245058 | 90 | 90 | 0.2025 |  | 16.875 | 1.35 | 10 | 28.225 | 11.35 | 0 | 28.225 |  | Pharmacy Margin | 683.4615188 |
| British Columbia | Irbesartan/HCTZ 300 mg/25 mg daily | 2447894 | 90 | 90 | 0.2719 |  | 22.65833 | 1.812666667 | 10 | 34.471 | 11.81266667 | 0 | 34.471 |  | Government Share | 0 |
| British Columbia | Levothyroxine 50 mcg daily | 2213192 | 90 | 90 | 0.0341 |  | 2.841667 | 0.227333333 | 10 | 13.069 | 10.22733333 | 0 | 13.069 |  | Patient Share | 4339.325919 |
| British Columbia | Atenolol 50mg daily | 2255545 | 90 | 90 | 0.1196 |  | 9.966667 | 0.797333333 | 10 | 20.764 | 10.79733333 | 0 | 20.764 |  |  |  |
| British Columbia | Liraglutide inj 1.8mg daily | 2351064 | 90 | 27 |  | 29.7367 | 802.8909 | 64.231272 | 10 | 877.122172 | 74.231272 | 0 | 877.122172 |  |  |  |
| British Columbia | Lorazepam .5 mg QHS | 655740 | 90 | 90 | 0.0388 |  | 3.233333 | 0.258666667 | 10 | 13.492 | 10.25866667 | 0 | 13.492 |  |  |  |
| British Columbia | ASAEC 81mg daily | 2237726 | 90 | 90 | 0.1035 |  | 8.625 | 0.69 | 10 | 19.315 | 10.69 | 0 | 19.315 |  |  |  |
| British Columbia | Calcium 500mg / Vitamin D 1000U BID | 80017748 | 90 | 180 |  | 0.09389 | 16.9002 | 9.100107692 | 0 | 26.00030769 | 9.100107692 | 0 | 26.00030769 |  |  |  |
| British Columbia/ Total Q1 |  |  |  |  |  |  |  |  |  |  | 170.8653797 | 0 | 1084.83148 |  |  |  |
| Manitoba | Metformin 1000mg BID | 2167786 | 90 | 360 |  | 0.0259 | 9.324 | 0 | 13.65 | 22.974 | 13.65 | 22.974 | 0 |  |  |  |
| Manitoba | Atorvastatin 40mg daily | 2295296 | 90 | 90 |  | 0.2459 | 22.131 | 0 | 13.65 | 35.781 | 13.65 | 35.781 | 0 |  | Manitoba /Annual costs |  |
| Manitoba | Omeprazole 20mg daily | 2245058 | 90 | 90 |  | 0.2401 | 21.609 | 0 | 13.65 | 35.259 | 13.65 | 35.259 | 0 |  | Pharmacy Margin | 494.4265846 |
| Manitoba | Irbesartan/HCTZ 300 mg/25 mg daily | 2447894 | 90 | 90 |  | 0.2293 | 20.637 | 0 | 13.65 | 34.287 | 13.65 | 34.287 | 0 |  | Government Share | 744.216 |
| Manitoba | Levothyroxine 50 mcg daily | 2213192 | 90 | 90 |  | 0.031 | 2.79 | 0 | 13.65 | 16.44 | 13.65 | 16.44 | 0 |  | Patient Share | 3430.810985 |
| Manitoba | Atenolol 50mg daily | 2255545 | 90 | 90 |  | 0.1162 | 10.458 | 0 | 13.65 | 24.108 | 13.65 | 24.108 | 0 |  |  |  |
| Manitoba | Liraglutide inj 1.8mg daily | 2351064 | 90 | 27 |  | 29.7367 | 802.8909 | 0 | 13.65 | 816.5409 | 13.65 | 0 | 816.5409 |  |  |  |
| Manitoba | Lorazepam .5 mg QHS | 655740 | 90 | 90 |  | 0.0395 | 3.555 | 0 | 13.65 | 17.205 | 13.65 | 17.205 | 0 |  |  |  |
| Manitoba | ASAEC 81mg daily | 2237726 | 90 | 90 |  | 0.1095 | 9.855 | 5.306538462 | 0 | 15.16153846 | 5.306538462 | 0 | 15.16153846 |  |  |  |
| Manitoba | Calcium 500mg / Vitamin D 1000U BID | 80017748 | 90 | 180 |  | 0.09389 | 16.9002 | 9.100107692 | 0 | 26.00030769 | 9.100107692 | 0 | 26.00030769 |  |  |  |
| Manitoba/ Total Q1 |  |  |  |  |  |  |  |  |  |  | 123.6066462 | 186.054 | 857.7027462 |  |  |  |
| New Brunswick | Metformin 1000mg BID | 2167786 | 90 | 360 | 0.0247 |  | 8.892 | 0.71136 | 11 | 20.60336 | 11.71136 | 14.422352 | 6.181008 |  |  |  |
| New Brunswick | Atorvastatin 40mg daily | 2295296 | 90 | 90 | 0.2342 |  | 21.078 | 1.68624 | 11 | 33.76424 | 12.68624 | 23.634968 | 10.129272 |  | New Brunswick /Annual costs |  |
| New Brunswick | Omeprazole 20mg daily | 2245058 | 90 | 90 | 0.2287 |  | 20.583 | 1.64664 | 11 | 33.22964 | 12.64664 | 23.260748 | 9.968892 |  | Pharmacy Margin | 693.2406326 |
| New Brunswick | Irbesartan/HCTZ 300 mg/25 mg daily | 2447894 | 90 | 90 | 0.2184 |  | 19.656 | 1.57248 | 11 | 32.22848 | 12.57248 | 22.559936 | 9.668544 |  | Government Share | 475.871872 |
| New Brunswick | Levothyroxine 50 mcg daily | 2213192 | 90 | 90 |  | 0.0311 | 2.879 | 0 | 11 | 13.879 | 11 | 9.7153 | 4.1637 |  | Patient Share | 3881.081161 |
| New Brunswick | Atenolol 50mg daily | 2255545 | 90 | 90 | 0.1107 |  | 9.963 | 0.79704 | 11 | 21.76004 | 11.79704 | 15.232028 | 6.528012 |  |  |  |
| New Brunswick | Liraglutide inj 1.8mg daily | 2351064 | 90 | 27 |  | 29.7367 | 802.8909 | 64.231272 | 11 | 878.122172 | 75.231272 | 0 | 878.122172 |  |  |  |
| New Brunswick | Lorazepam .5 mg QHS | 655740 | 90 | 90 | 0.0359 |  | 3.231 | 0.25848 | 11 | 14.48948 | 11.25848 | 10.142636 | 4.346844 |  |  |  |
| New Brunswick | ASAEC 81mg daily | 2237726 | 90 | 90 |  | 0.1095 | 9.855 | 5.306538462 | 0 | 15.16153846 | 5.306538462 | 0 | 15.16153846 |  |  |  |
| New Brunswick | Calcium 500mg / Vitamin D 1000U BID | 80017748 | 90 | 180 |  | 0.09389 | 16.9002 | 9.100107692 | 0 | 26.00030769 | 9.100107692 | 0 | 26.00030769 |  |  |  |
| New Brunswick/ Total Q1 |  |  |  |  |  |  |  |  |  |  | 173.3101582 | 118.967968 | 970.2702902 |  |  |  |
| Newfoundland and Labrador | Metformin 1000mg BID | 2167786 | 90 | 360 |  | 0.0269 | 8.925346 | 0.758654378 | 12 | 21.684 | 12.75865438 | 15.684 | 6 |  |  |  |
| Newfoundland and Labrador | Atorvastatin 40mg daily | 2295296 | 90 | 90 |  | 0.2553 | 21.17696 | 1.800041475 | 12 | 34.977 | 13.80004147 | 28.977 | 6 |  | Newfoundland and Labrador / Annual costs |  |
| Newfoundland and Labrador | Omeprazole 20mg daily | 2245058 | 90 | 90 |  | 0.2493 | 20.67926 | 1.757737327 | 12 | 34.437 | 13.75773733 | 28.437 | 6 |  | Pharmacy Margin | 872.108175 |
| Newfoundland and Labrador | Irbesartan/HCTZ 300 mg/25 mg daily | 2447894 | 90 | 90 |  | 0.2381 | 19.75023 | 1.678769585 | 12 | 33.429 | 13.67876959 | 27.429 | 6 |  | Government Share | 543.804 |
| Newfoundland and Labrador | Levothyroxine 50 mcg daily | 2213192 | 90 | 90 |  | 0.0338 | 2.803687 | 0.238313364 | 12 | 15.042 | 12.23831336 | 9.042 | 6 |  | Patient Share | 3993.251709 |
| Newfoundland and Labrador | Atenolol 50mg daily | 2255545 | 90 | 90 |  | 0.1207 | 10.01198 | 0.851018433 | 12 | 22.863 | 12.85101843 | 16.863 | 6 |  |  |  |
| Newfoundland and Labrador | Liraglutide inj 1.8mg daily | 2351064 | 90 | 27 |  | 29.7367 | 802.8909 | 72.260181 | 40 | 915.151081 | 112.260181 | 0 | 915.151081 |  |  |  |
| Newfoundland and Labrador | Lorazepam .5 mg QHS | 655740 | 90 | 90 |  | 0.0391 | 3.243318 | 0.275682028 | 12 | 15.519 | 12.27568203 | 9.519 | 6 |  |  |  |
| Newfoundland and Labrador | ASAEC 81mg daily | 2237726 | 90 | 90 |  | 0.1095 | 9.855 | 5.306538462 | 0 | 15.16153846 | 5.306538462 | 0 | 15.16153846 |  |  |  |
| Newfoundland and Labrador | Calcium 500mg / Vitamin D 1000U BID | 80017748 | 90 | 180 |  | 0.09389 | 16.9002 | 9.100107692 | 0 | 26.00030769 | 9.100107692 | 0 | 26.00030769 |  |  |  |
| Newfoundland and Labrador/ Total Q1 |  |  |  |  |  |  |  |  |  |  | 218.0270437 | 135.951 | 998.3129272 |  |  |  |
| Nova Scotia | Metformin 1000mg BID | 2167786 | 90 | 360 | 0.0247 |  | 8.892 | 0.71136 | 11.95 | 21.55336 | 12.66136 | 15.087352 | 6.466008 |  |  |  |
| Nova Scotia | Atorvastatin 40mg daily | 2295296 | 90 | 90 | 0.2342 |  | 21.078 | 1.68624 | 11.95 | 34.71424 | 13.63624 | 24.299968 | 10.414272 |  | Nova Scotia /Annual costs |  |
| Nova Scotia | Omeprazole 20mg daily | 2245058 | 90 | 90 | 0.2287 |  | 20.583 | 1.64664 | 11.95 | 34.17964 | 13.59664 | 23.925748 | 10.253892 |  | Pharmacy Margin | 805.1242026 |
| Nova Scotia | Irbesartan/HCTZ 300 mg/25 mg daily | 2447894 | 90 | 90 | 0.2184 |  | 19.656 | 1.57248 | 11.95 | 33.17848 | 13.52248 | 23.224936 | 9.953544 |  | Government Share | 495.230008 |
| Nova Scotia | Levothyroxine 50 mcg daily | 2213192 | 90 | 90 |  |  | 2.844 | 0.29862 | 11.95 | 15.09262 | 12.24862 | 10.564834 | 4.527786 |  | Patient Share | 3973.466595 |
| Nova Scotia | Atenolol 50mg daily | 2255545 | 90 | 90 | 0.1107 |  | 9.963 | 0.79704 | 11.95 | 22.71004 | 12.74704 | 15.897028 | 6.813012 |  |  |  |
| Nova Scotia | Liraglutide inj 1.8mg daily | 2351064 | 90 | 27 |  | 29.7367 | 802.8909 | 84.3035445 | 11.95 | 899.1444445 | 96.2535445 | 0 | 899.1444445 |  |  |  |
| Nova Scotia | Lorazepam .5 mg QHS | 655740 | 90 | 90 | 0.0359 |  | 3.231 | 0.25848 | 11.95 | 15.43948 | 12.20848 | 10.807636 | 4.631844 |  |  |  |
| Nova Scotia | ASAEC 81mg daily | 2237726 | 90 | 90 |  | 0.1095 | 9.855 | 5.306538462 | 0 | 15.16153846 | 5.306538462 | 0 | 15.16153846 |  |  |  |
| Nova Scotia | Calcium 500mg / Vitamin D 1000U BID | 80017748 | 90 | 180 |  | 0.09389 | 16.9002 | 9.100107692 | 0 | 26.00030769 | 9.100107692 | 0 | 26.00030769 |  |  |  |
| Nova Scotia/ Total Q1 |  |  |  |  |  |  |  |  |  |  | 201.2810507 | 123.807502 | 993.3666487 |  |  |  |
| Ontario | Metformin 1000mg BID | 2167786 | 90 | 360 |  | 0.0247 | 8.892 | 0.71136 | 8.83 | 18.43336 | 9.54136 | 12.32336 | 6.11 |  |  |  |
| Ontario | Atorvastatin 40mg daily | 2295296 | 90 | 90 |  | 0.2342 | 21.078 | 1.68624 | 8.83 | 31.59424 | 10.51624 | 25.48424 | 6.11 |  | Ontario /Annual costs |  |
| Ontario | Omeprazole 20mg daily | 2245058 | 90 | 90 |  | 0.2287 | 20.583 | 1.64664 | 8.83 | 31.05964 | 10.47664 | 24.94964 | 6.11 |  | Pharmacy Margin | 624.7107126 |
| Ontario | Irbesartan/HCTZ 300 mg/25 mg daily | 2447894 | 90 | 90 |  | 0.2184 | 19.656 | 1.57248 | 8.83 | 30.05848 | 10.40248 | 23.94848 | 6.11 |  | Government Share | 448.74704 |
| Ontario | Levothyroxine 50 mcg daily | 2213192 | 90 | 90 |  | 0.0316 | 2.844 | 0.22752 | 8.83 | 11.90152 | 9.05752 | 5.79152 | 6.11 |  | Patient Share | 3839.536073 |
| Ontario | Atenolol 50mg daily | 2255545 | 90 | 90 |  | 0.1107 | 9.963 | 0.79704 | 8.83 | 19.59004 | 9.62704 | 13.48004 | 6.11 |  |  |  |
| Ontario | Liraglutide inj 1.8mg daily | 2351064 | 90 | 27 |  | 29.7367 | 802.8909 | 64.231272 | 8.83 | 875.952172 | 73.061272 | 0 | 875.952172 |  |  |  |
| Ontario | Lorazepam .5 mg QHS | 655740 | 90 | 90 |  | 0.0359 | 3.231 | 0.25848 | 8.83 | 12.31948 | 9.08848 | 6.20948 | 6.11 |  |  |  |
| Ontario | ASAEC 81mg daily | 2237726 | 90 | 90 |  | 0.1095 | 9.855 | 5.306538462 | 0 | 15.16153846 | 5.306538462 | 0 | 15.16153846 |  |  |  |
| Ontario | Calcium 500mg / Vitamin D 1000U BID | 80017748 | 90 | 180 |  | 0.09389 | 16.9002 | 9.100107692 | 0 | 26.00030769 | 9.100107692 | 0 | 26.00030769 |  |  |  |
| Ontario/ Total Q1 |  |  |  |  |  |  |  |  |  |  | 156.1776782 | 112.18676 | 959.8840182 |  |  |  |
| Prince Edward Island | Metformin 1000mg BID | 2167786 | 90 | 360 | 0.0247 |  | 8.892 | 0.53352 | 12.36 | 21.78552 | 12.89352 | 5.84552 | 15.94 |  |  |  |
| Prince Edward Island | Atorvastatin 40mg daily | 2295296 | 90 | 90 | 0.2342 |  | 21.078 | 1.26468 | 12.36 | 34.70268 | 13.62468 | 18.76268 | 15.94 |  | Prince Edward Island / Annual costs |  |
| Prince Edward Island | Omeprazole 20mg daily | 2245058 | 90 | 90 | 0.2287 |  | 20.583 | 1.23498 | 12.36 | 34.17798 | 13.59498 | 18.23798 | 15.94 |  | Pharmacy Margin | 666.5267206 |
| Prince Edward Island | Irbesartan/HCTZ 300 mg/25 mg daily | 2447894 | 90 | 90 | 0.2184 |  | 19.656 | 1.17936 | 12.36 | 33.19536 | 13.53936 | 17.25536 | 15.94 |  | Government Share | 307.13432 |
| Prince Edward Island | Levothyroxine 50 mcg daily | 2213192 | 90 | 90 |  | 0.031 | 2.79 | 0.1674 | 12.36 | 15.3174 | 12.5274 | 4.8374 | 10.48 |  | Patient Share | 4022.748801 |
| Prince Edward Island | Atenolol 50mg daily | 2255545 | 90 | 90 | 0.1107 |  | 9.963 | 0.59778 | 12.36 | 22.92078 | 12.95778 | 6.98078 | 15.94 |  |  |  |
| Prince Edward Island | Liraglutide inj 1.8mg daily | 2351064 | 90 | 27 |  | 29.7367 | 802.8909 | 48.173454 | 12.36 | 863.424354 | 60.533454 | 0 | 863.424354 |  |  |  |
| Prince Edward Island | Lorazepam .5 mg QHS | 655740 | 90 | 90 | 0.0359 |  | 3.231 | 0.19386 | 12.36 | 15.78486 | 12.55386 | 4.86386 | 10.921 |  |  |  |
| Prince Edward Island | ASAEC 81mg daily | 2237726 | 90 | 90 |  | 0.1095 | 9.855 | 5.306538462 | 0 | 15.16153846 | 5.306538462 | 0 | 15.16153846 |  |  |  |
| Prince Edward Island | Calcium 500mg / Vitamin D 1000U BID | 80017748 | 90 | 180 |  | 0.09389 | 16.9002 | 9.100107692 | 0 | 26.00030769 | 9.100107692 | 0 | 26.00030769 |  |  |  |
| Prince Edward Island/ Total Q1 |  |  |  |  |  |  |  |  |  |  | 166.6316802 | 76.78358 | 1005.6872 |  |  |  |
| Quebec | Metformin 1000mg BID | 2167786 | 90 | 360 |  | 0.0247 | 8.892 | 0.57798 | 27 | 36.46998 | 27.57798 | 23.74195698 | 12.72802302 |  |  |  |
| Quebec | Atorvastatin 40mg daily | 2295296 | 90 | 90 |  | 0.2342 | 21.078 | 1.37007 | 27 | 49.44807 | 28.37007 | 32.19069357 | 17.25737643 |  | Quebec /Annual costs |  |
| Quebec | Omeprazole 20mg daily | 2245058 | 90 | 90 |  | 0.2287 | 20.583 | 1.337895 | 27 | 48.920895 | 28.337895 | 31.84750265 | 17.07339236 |  | Pharmacy Margin | 1104.219125 |
| Quebec | Irbesartan/HCTZ 300 mg/25 mg daily | 2447894 | 90 | 90 |  | 0.2184 | 19.656 | 1.27764 | 27 | 47.93364 | 28.27764 | 31.20479964 | 16.72884036 |  | Government Share | 2510.06626 |
| Quebec | Levothyroxine 50 mcg daily | 2213192 | 90 | 90 |  | 0.0274 | 2.466 | 0.16029 | 27 | 29.62629 | 27.16029 | 19.28671479 | 10.33957521 |  | Patient Share | 1510.289665 |
| Quebec | Atenolol 50mg daily | 2255545 | 90 | 90 |  | 0.1107 | 9.963 | 0.647595 | 27 | 37.610595 | 27.647595 | 24.48449735 | 13.12609766 |  |  |  |
| Quebec | Liraglutide inj 1.8mg daily | 2351064 | 90 | 27 |  | 22.83 | 616.41 | 40.06665 | 27 | 683.47665 | 67.06665 | 444.9432992 | 238.5333509 |  |  |  |
| Quebec | Lorazepam .5 mg QHS | 655740 | 90 | 90 |  | 0.0359 | 3.231 | 0.210015 | 27 | 30.441015 | 27.210015 | 19.81710077 | 10.62391424 |  |  |  |
| Quebec | ASAEC 81mg daily | 2237726 | 90 | 90 |  | 0.1095 | 9.855 | 5.306538462 | 0 | 15.16153846 | 5.306538462 | 0 | 15.16153846 |  |  |  |
| Quebec | Calcium 500mg / Vitamin D 1000U BID | 80017748 | 90 | 180 |  | 0.09389 | 16.9002 | 9.100107692 | 0 | 26.00030769 | 9.100107692 | 0 | 26.00030769 |  |  |  |
| Quebec/Total Q1 |  |  |  |  |  |  |  |  |  |  | 276.0547812 | 627.5165649 | 377.5724163 |  |  |  |
| Saskatchewan | Metformin 1000mg BID | 2167786 | 90 | 360 |  | 0.0247 | 8.892 | 1.3338 | 11.4 | 21.6258 | 12.7338 | 0 | 21.6258 |  |  |  |
| Saskatchewan | Atorvastatin 40mg daily | 2295296 | 90 | 90 |  | 0.2342 | 21.078 | 2.1078 | 11.4 | 34.5858 | 13.5078 | 9.5858 | 25 |  | Saskatchewan /Annual costs |  |
| Saskatchewan | Omeprazole 20mg daily | 2245058 | 90 | 90 |  | 0.2287 | 20.583 | 2.0583 | 11.4 | 34.0413 | 13.4583 | 9.0413 | 25 |  | Pharmacy Margin | 545.5563846 |
| Saskatchewan | Irbesartan/HCTZ 300 mg/25 mg daily | 2447894 | 90 | 90 |  | 0.2184 | 19.656 | 1.9656 | 11.4 | 33.0216 | 13.3656 | 8.0216 | 25 |  | Government Share | 106.5948 |
| Saskatchewan | Levothyroxine 50 mcg daily | 2213192 | 90 | 90 |  | 0.0316 | 2.844 | 0.8532 | 11.4 | 15.0972 | 12.2532 | 0 | 15.0972 |  | Patient Share | 4102.533985 |
| Saskatchewan | Atenolol 50mg daily | 2255545 | 90 | 90 |  | 0.1107 | 9.963 | 1.49445 | 11.4 | 22.85745 | 12.89445 | 0 | 22.85745 |  |  |  |
| Saskatchewan | Liraglutide inj 1.8mg daily | 2351064 | 90 | 27 |  | 29.7367 | 802.8909 | 20 | 11.4 | 834.2909 | 31.4 | 0 | 834.2909 |  |  |  |
| Saskatchewan | Lorazepam .5 mg QHS | 655740 | 90 | 90 |  | 0.0359 | 3.231 | 0.9693 | 11.4 | 15.6003 | 12.3693 | 0 | 15.6003 |  |  |  |
| Saskatchewan | ASAEC 81mg daily | 2237726 | 90 | 90 |  | 0.1095 | 9.855 | 5.306538462 | 0 | 15.16153846 | 5.306538462 | 0 | 15.16153846 |  |  |  |
| Saskatchewan | Calcium 500mg / Vitamin D 1000U BID | 80017748 | 90 | 180 |  | 0.09389 | 16.9002 | 9.100107692 | 0 | 26.00030769 | 9.100107692 | 0 | 26.00030769 |  |  |  |
| Saskatchewan/ Total Q1 |  |  |  |  |  |  |  |  |  |  | 136.3890962 | 26.6487 | 1025.633496 |  |  |  |
| Northwest Territories | Metformin 1000mg BID | 2167786 | 90 | 360 |  | 0.0247 | 8.892 | 1.6325712 | 12.72 | 23.2445712 | 14.3525712 | 23.2445712 | 0 |  |  |  |
| Northwest Territories | Atorvastatin 40mg daily | 2295296 | 90 | 90 |  | 0.2342 | 21.078 | 3.8699208 | 12.72 | 37.6679208 | 16.5899208 | 37.6679208 | 0 |  | Northwest Territories /Annual costs |  |
| Northwest Territories | Omeprazole 20mg daily | 2245058 | 90 | 90 |  | 0.2287 | 20.583 | 3.7790388 | 12.72 | 37.0820388 | 16.4990388 | 37.0820388 | 0 |  | Pharmacy Margin | 727.9667238 |
| Northwest Territories | Irbesartan/HCTZ 300 mg/25 mg daily | 2447894 | 90 | 90 |  | 0.2184 | 19.656 | 3.6088416 | 12.72 | 35.9848416 | 16.3288416 | 35.9848416 | 0 |  | Government share | 764.2321392 |
| Northwest Territories | Levothyroxine 50 mcg daily | 2213192 | 90 | 90 |  | 0.031 | 2.79 | 0.512244 | 12.72 | 16.022244 | 13.232244 | 16.022244 | 0 |  | Patient share | 3627.090985 |
| Northwest Territories | Atenolol 50mg daily | 2255545 | 90 | 90 |  | 0.1107 | 9.963 | 1.8292068 | 12.72 | 24.5122068 | 14.5492068 | 24.5122068 | 0 |  |  |  |
| Northwest Territories | Liraglutide inj 1.8mg daily | 2351064 | 90 | 27 |  | 29.7367 | 802.8909 | 50 | 12.72 | 865.6109 | 62.72 | 0 | 865.6109 |  |  |  |
| Northwest Territories | Lorazepam .5 mg QHS | 655740 | 90 | 90 |  | 0.0359 | 3.231 | 0.5932116 | 12.72 | 16.5442116 | 13.3132116 | 16.5442116 | 0 |  |  |  |
| Northwest Territories | ASAEC 81mg daily | 2237726 | 90 | 90 |  | 0.1095 | 9.855 | 5.306538462 | 0 | 15.16153846 | 5.306538462 | 0 | 15.16153846 |  |  |  |
| Northwest Territories | Calcium 500mg / Vitamin D 1000U BID | 80017748 | 90 | 180 |  | 0.09389 | 16.9002 | 9.100107692 | 0 | 26.00030769 | 9.100107692 | 0 | 26.00030769 |  |  |  |
| Northwest Territories |  |  |  |  |  |  |  |  |  |  | 181.991681 | 191.0580348 | 906.7727462 |  |  |  |
| Nunavut | Metformin 1000mg BID | 2167786 | 90 | 360 |  | 0.0247 | 8.892 | 2.1127392 | 16.95 | 27.9547392 | 19.0627392 | 27.9547392 | 0 |  |  |  |
| Nunavut | Atorvastatin 40mg daily | 2295296 | 90 | 90 |  | 0.2342 | 21.078 | 5.0081328 | 16.95 | 43.0361328 | 21.9581328 | 43.0361328 | 0 |  | Nunavut /Annual costs |  |
| Nunavut | Omeprazole 20mg daily | 2245058 | 90 | 90 |  | 0.2287 | 20.583 | 4.8905208 | 16.95 | 42.4235208 | 21.8405208 | 42.4235208 | 0 |  | Pharmacy Margin | 881.9444118 |
| Nunavut | Irbesartan/HCTZ 300 mg/25 mg daily | 2447894 | 90 | 90 |  | 0.2184 | 19.656 | 4.6702656 | 16.95 | 41.2762656 | 21.6202656 | 41.2762656 | 0 |  | Government share | 901.2898272 |
| Nunavut | Levothyroxine 50 mcg daily | 2213192 | 90 | 90 |  | 0.031 | 2.79 | 0.662904 | 16.95 | 20.402904 | 17.612904 | 20.402904 | 0 |  | Patient share | 3644.010985 |
| Nunavut | Atenolol 50mg daily | 2255545 | 90 | 90 |  | 0.1107 | 9.963 | 2.3672088 | 16.95 | 29.2802088 | 19.3172088 | 29.2802088 | 0 |  |  |  |
| Nunavut | Liraglutide inj 1.8mg daily | 2351064 | 90 | 27 |  | 29.7367 | 802.8909 | 50 | 16.95 | 869.8409 | 66.95 | 0 | 869.8409 |  |  |  |
| Nunavut | Lorazepam .5 mg QHS | 655740 | 90 | 90 |  | 0.0359 | 3.231 | 0.7676856 | 16.95 | 20.9486856 | 17.7176856 | 20.9486856 | 0 |  |  |  |
| Nunavut | ASAEC 81mg daily | 2237726 | 90 | 90 |  | 0.1095 | 9.855 | 5.306538462 | 0 | 15.16153846 | 5.306538462 | 0 | 15.16153846 |  |  |  |
| Nunavut | Calcium 500mg / Vitamin D 1000U BID | 80017748 | 90 | 180 |  | 0.09389 | 16.9002 | 9.100107692 | 0 | 26.00030769 | 9.100107692 | 0 | 26.00030769 |  |  |  |
| Nunavut |  |  |  |  |  |  |  |  |  |  | 220.486103 | 225.3224568 | 911.0027462 |  |  |  |
| Yukon | Metformin 1000mg BID | 2167786 | 90 | 360 |  | 0.0247 | 8.892 | 2.1127392 | 12.72 | 23.7247392 | 14.8327392 | 23.7247392 | 0 |  |  |  |
| Yukon | Atorvastatin 40mg daily | 2295296 | 90 | 90 |  | 0.2342 | 21.078 | 5.0081328 | 12.72 | 38.8061328 | 17.7281328 | 38.8061328 | 0 |  | Yukon /Annual costs |  |
| Yukon | Omeprazole 20mg daily | 2245058 | 90 | 90 |  | 0.2287 | 20.583 | 4.8905208 | 12.72 | 38.1935208 | 17.6105208 | 38.1935208 | 0 |  | Pharmacy Margin | 783.3462996 |
| Yukon | Irbesartan/HCTZ 300 mg/25 mg daily | 2357410 | 90 | 90 |  | 0.2184 | 19.656 | 4.6702656 | 12.72 | 37.0462656 | 17.3902656 | 37.0462656 | 0 |  | Government Share | 870.7538688 |
| Yukon | Levothyroxine 50 mcg daily | 2213192 | 90 | 90 |  | 0.03 | 2.7 | 0.64152 | 12.72 | 16.06152 | 13.36152 | 16.06152 | 0 |  | Patient Share | 3566.444831 |
| Yukon | Atenolol 50mg daily | 2255545 | 90 | 90 |  | 0.1107 | 9.963 | 2.3672088 | 12.72 | 25.0502088 | 15.0872088 | 25.0502088 | 0 |  |  |  |
| Yukon | Liraglutide inj 1.8mg daily | 2351064 | 90 | 27 |  | 29.7367 | 802.8909 | 50 | 12.72 | 865.6109 | 62.72 | 0 | 865.6109 |  |  |  |
| Yukon | Lorazepam .5 mg QHS | 655740 | 90 | 90 |  | 0.04 | 3.6 | 0.85536 | 12.72 | 17.17536 | 13.57536 | 17.17536 | 0 |  |  |  |
| Yukon | ASAEC 81mg daily | 2237726 | 90 | 90 |  | 0.08 | 7.2 | 1.71072 | 12.72 | 21.63072 | 14.43072 | 21.63072 | 0 |  |  |  |
| Yukon | Calcium 500mg / Vitamin D 1000U BID | 80017748 | 90 | 180 |  | 0.09389 | 16.9002 | 9.100107692 | 0 | 26.00030769 | 9.100107692 | 0 | 26.00030769 |  |  |  |
| Yukon |  |  |  |  |  |  |  |  |  |  | 195.8365749 | 217.6884672 | 891.6112077 |  |  |  |

Supplementary Table 8 – Scenario 7

| Province | Drug | DIN | Days Supply | Quantity | MAC/Unit | Drug Cost / Unit | Drug Cost | Markup | Dispensing Fee | Total Cost | Pharmacy Margin $ | Government Share | Patient Share |  |  |  |
| --- | --- | --- | --- | --- | --- | --- | --- | --- | --- | --- | --- | --- | --- | --- | --- | --- |
| Alberta | Metformin 1000mg BID | 2167786 | 90 | 360 |  | 0.0247 | 8.892 | 0.9078732 | 12.15 | 21.9498732 | 13.0578732 | 15.36491124 | 6.58496196 |  |  |  |
| Alberta | Atorvastatin 40mg daily | 2295296 | 90 | 90 |  | 0.2342 | 21.078 | 2.1520638 | 12.15 | 35.3800638 | 14.3020638 | 24.76604466 | 10.61401914 |  | Alberta /Annual costs |  |
| Alberta | Omeprazole 20mg daily | 2245058 | 90 | 90 |  | 0.2287 | 20.583 | 2.1015243 | 12.15 | 34.8345243 | 14.2515243 | 24.38416701 | 10.45035729 |  | Pharmacy Margin | 490.6517714 |
| Alberta | Irbesartan/HCTZ 300 mg/25 mg daily | 2447894 | 90 | 90 |  | 0.2184 | 19.656 | 2.0068776 | 12.15 | 33.8128776 | 14.1568776 | 23.66901432 | 10.14386328 |  | Government share | 606.3265507 |
| Alberta | Levothyroxine 50 mcg daily | 2213192 | 90 | 90 |  | 0.031 | 2.79 | 0.284859 | 12.15 | 15.224859 | 12.434859 | 10.6574013 | 4.5674577 |  | Patient share | 424.5016206 |
| Alberta | Atenolol 50mg daily | 2255545 | 90 | 90 |  | 0.1107 | 9.963 | 1.0172223 | 12.15 | 23.1302223 | 13.1672223 | 16.19115561 | 6.93906669 |  |  |  |
| Alberta | Levemir cartridge 100 unit / ml inj | 2271842 | 90 | 3 |  | 7.1013 | 21.3039 | 2.17512819 | 12.15 | 35.62902819 | 14.32512819 | 24.94031973 | 10.68870846 |  |  |  |
| Alberta | Lorazepam 1mg QHS | 655759 | 90 | 90 |  | 0.0447 | 4.023 | 0.4107483 | 12.15 | 16.5837483 | 12.5607483 | 11.60862381 | 4.97512449 |  |  |  |
| Alberta | ASAEC 81mg daily | 2237726 | 90 | 90 |  | 0.1095 | 9.855 | 5.306538462 | 0 | 15.16153846 | 5.306538462 | 0 | 15.16153846 |  |  |  |
| Alberta | Calcium 500mg / Vitamin D 1000U BID | 80017748 | 90 | 180 |  | 0.09389 | 16.9002 | 9.100107692 | 0 | 26.00030769 | 9.100107692 | 0 | 26.00030769 |  |  |  |
| Alberta/ Total Q1 |  |  |  |  |  |  |  |  |  |  | 122.6629428 | 151.5816377 | 106.1254052 |  |  |  |
| British Columbia | Metformin 1000mg BID | 2167786 | 90 | 360 | 0.0267 |  | 8.9 | 0.712 | 10 | 19.612 | 10.712 | 0 | 19.612 |  |  |  |
| British Columbia | Atorvastatin 40mg daily | 2295296 | 90 | 90 | 0.2529 |  | 21.075 | 1.686 | 10 | 32.761 | 11.686 | 0 | 32.761 |  | British Columbia /Annual costs |  |
| British Columbia | Omeprazole 20mg daily | 2245058 | 90 | 90 | 0.2025 |  | 16.875 | 1.35 | 10 | 28.225 | 11.35 | 0 | 28.225 |  | Pharmacy Margin | 433.6070121 |
| British Columbia | Irbesartan/HCTZ 300 mg/25 mg daily | 2447894 | 90 | 90 | 0.2719 |  | 22.65833 | 1.812666667 | 10 | 34.471 | 11.81266667 | 0 | 34.471 |  | Government share | 0 |
| British Columbia | Levothyroxine 50 mcg daily | 2213192 | 90 | 90 | 0.0341 |  | 2.841667 | 0.227333333 | 10 | 13.069 | 10.22733333 | 0 | 13.069 |  | Patient share | 966.2900788 |
| British Columbia | Atenolol 50mg daily | 2255545 | 90 | 90 | 0.1196 |  | 9.966667 | 0.797333333 | 10 | 20.764 | 10.79733333 | 0 | 20.764 |  |  |  |
| British Columbia | Levemir cartridge 100 unit / ml inj | 2271842 | 90 | 3 |  | 7.1013 | 21.3039 | 1.704312 | 10 | 33.008212 | 11.704312 | 0 | 33.008212 |  |  |  |
| British Columbia | Lorazepam 1mg QHS | 655759 | 90 | 90 | 0.0483 |  | 4.025 | 0.322 | 10 | 14.347 | 10.322 | 0 | 14.347 |  |  |  |
| British Columbia | ASAEC 81mg daily | 2237726 | 90 | 90 | 0.1035 |  | 8.625 | 0.69 | 10 | 19.315 | 10.69 | 0 | 19.315 |  |  |  |
| British Columbia | Calcium 500mg / Vitamin D 1000U BID | 80017748 | 90 | 180 | 0 | 0.09389 | 16.9002 | 9.100107692 | 0 | 26.00030769 | 9.100107692 | 0 | 26.00030769 |  |  |  |
| British Columbia/ Total Q1 |  |  |  |  |  |  |  |  |  |  | 108.401753 | 0 | 241.5725197 |  |  |  |
| Manitoba | Metformin 1000mg BID | 2167786 | 90 | 360 |  | 0.0259 | 9.324 | 0 | 13.65 | 22.974 | 13.65 | 22.974 | 0 |  |  |  |
| Manitoba | Atorvastatin 40mg daily | 2295296 | 90 | 90 |  | 0.2459 | 22.131 | 0 | 13.65 | 35.781 | 13.65 | 35.781 | 0 |  | Manitoba /Annual costs |  |
| Manitoba | Omeprazole 20mg daily | 2245058 | 90 | 90 |  | 0.2401 | 21.609 | 0 | 13.65 | 35.259 | 13.65 | 35.259 | 0 |  | Pharmacy Margin | 494.4265846 |
| Manitoba | Irbesartan/HCTZ 300 mg/25 mg daily | 2447894 | 90 | 90 |  | 0.2293 | 20.637 | 0 | 13.65 | 34.287 | 13.65 | 34.287 | 0 |  | Government share | 747.708 |
| Manitoba | Levothyroxine 50 mcg daily | 2213192 | 90 | 90 |  | 0.031 | 2.79 | 0 | 13.65 | 16.44 | 13.65 | 16.44 | 0 |  | Patient share | 304.4629846 |
| Manitoba | Atenolol 50mg daily | 2255545 | 90 | 90 |  | 0.1162 | 10.458 | 0 | 13.65 | 24.108 | 13.65 | 24.108 | 0 |  |  |  |
| Manitoba | Levemir cartridge 100 unit / ml inj | 2271842 | 90 | 3 |  | 7.1013 | 21.3039 | 0 | 13.65 | 34.9539 | 13.65 | 0 | 34.9539 |  |  |  |
| Manitoba | Lorazepam 1mg QHS | 655759 | 90 | 90 |  | 0.0492 | 4.428 | 0 | 13.65 | 18.078 | 13.65 | 18.078 | 0 |  |  |  |
| Manitoba | ASAEC 81mg daily | 2237726 | 90 | 90 |  | 0.1095 | 9.855 | 5.306538462 | 0 | 15.16153846 | 5.306538462 | 0 | 15.16153846 |  |  |  |
| Manitoba | Calcium 500mg / Vitamin D 1000U BID | 80017748 | 90 | 180 |  | 0.09389 | 16.9002 | 9.100107692 | 0 | 26.00030769 | 9.100107692 | 0 | 26.00030769 |  |  |  |
| Manitoba/ Total Q1 |  |  |  |  |  |  |  |  |  |  | 123.6066462 | 186.927 | 76.11574615 |  |  |  |
| New Brunswick | Metformin 1000mg BID | 2167786 | 90 | 360 | 0.0247 |  | 8.892 | 0.71136 | 11 | 20.60336 | 11.71136 | 14.422352 | 6.181008 |  |  |  |
| New Brunswick | Atorvastatin 40mg daily | 2295296 | 90 | 90 | 0.2342 |  | 21.078 | 1.68624 | 11 | 33.76424 | 12.68624 | 23.634968 | 10.129272 |  | New Brunswick /Annual costs |  |
| New Brunswick | Omeprazole 20mg daily | 2245058 | 90 | 90 | 0.2287 |  | 20.583 | 1.64664 | 11 | 33.22964 | 12.64664 | 23.260748 | 9.968892 |  | Pharmacy Margin | 436.5689846 |
| New Brunswick | Irbesartan/HCTZ 300 mg/25 mg daily | 2447894 | 90 | 90 | 0.2184 |  | 19.656 | 1.57248 | 11 | 32.22848 | 12.57248 | 22.559936 | 9.668544 |  | Government share | 568.9418 |
| New Brunswick | Levothyroxine 50 mcg daily | 2213192 | 90 | 90 |  | 0.0311 | 2.879 | 0 | 11 | 13.879 | 11 | 9.7153 | 4.1637 |  | Patient share | 408.4795846 |
| New Brunswick | Atenolol 50mg daily | 2255545 | 90 | 90 | 0.1107 |  | 9.963 | 0.79704 | 11 | 21.76004 | 11.79704 | 15.232028 | 6.528012 |  |  |  |
| New Brunswick | Levemir cartridge 100 unit / ml inj | 2271842 | 90 | 3 |  | 7.1013 | 21.3839 | 0 | 11 | 32.3839 | 11 | 22.66873 | 9.71517 |  |  |  |
| New Brunswick | Lorazepam 1mg QHS | 655759 | 90 | 90 | 0.0447 |  | 4.023 | 0.32184 | 11 | 15.34484 | 11.32184 | 10.741388 | 4.603452 |  |  |  |
| New Brunswick | ASAEC 81mg daily | 2237726 | 90 | 90 |  | 0.1095 | 9.855 | 5.306538462 | 0 | 15.16153846 | 5.306538462 | 0 | 15.16153846 |  |  |  |
| New Brunswick | Calcium 500mg / Vitamin D 1000U BID | 80017748 | 90 | 180 |  | 0.09389 | 16.9002 | 9.100107692 | 0 | 26.00030769 | 9.100107692 | 0 | 26.00030769 |  |  |  |
| New Brunswick/ Total Q1 |  |  |  |  |  |  |  |  |  |  | 109.1422462 | 142.23545 | 102.1198962 |  |  |  |
| Newfoundland and Labrador | Metformin 1000mg BID | 2167786 | 90 | 360 |  | 0.0269 | 8.925346 | 0.758654378 | 12 | 21.684 | 12.75865438 | 15.684 | 6 |  |  |  |
| Newfoundland and Labrador | Atorvastatin 40mg daily | 2295296 | 90 | 90 |  | 0.2553 | 21.17696 | 1.800041475 | 12 | 34.977 | 13.80004147 | 28.977 | 6 |  | Newfoundland and Labrador / Annual costs |  |
| Newfoundland and Labrador | Omeprazole 20mg daily | 2245058 | 90 | 90 |  | 0.2493 | 20.67926 | 1.757737327 | 12 | 34.437 | 13.75773733 | 28.437 | 6 |  | Pharmacy Margin | 479.0076015 |
| Newfoundland and Labrador | Irbesartan/HCTZ 300 mg/25 mg daily | 2447894 | 90 | 90 |  | 0.2381 | 19.75023 | 1.678769585 | 12 | 33.429 | 13.67876959 | 27.429 | 6 |  | Government share | 547.26 |
| Newfoundland and Labrador | Levothyroxine 50 mcg daily | 2213192 | 90 | 90 |  | 0.0338 | 2.803687 | 0.238313364 | 12 | 15.042 | 12.23831336 | 9.042 | 6 |  | Patient share | 473.5323886 |
| Newfoundland and Labrador | Atenolol 50mg daily | 2255545 | 90 | 90 |  | 0.1207 | 10.01198 | 0.851018433 | 12 | 22.863 | 12.85101843 | 16.863 | 6 |  |  |  |
| Newfoundland and Labrador | Levemir cartridge 100 unit / ml inj | 2271842 | 90 | 3 |  | 7.1013 | 21.3039 | 1.917351 | 12 | 35.221251 | 13.917351 | 0 | 35.221251 |  |  |  |
| Newfoundland and Labrador | Lorazepam 1mg QHS | 655759 | 90 | 90 |  | 0.0487 | 4.039631 | 0.343368664 | 12 | 16.383 | 12.34336866 | 10.383 | 6 |  |  |  |
| Newfoundland and Labrador | ASAEC 81mg daily | 2237726 | 90 | 90 |  | 0.1095 | 9.855 | 5.306538462 | 0 | 15.16153846 | 5.306538462 | 0 | 15.16153846 |  |  |  |
| Newfoundland and Labrador | Calcium 500mg / Vitamin D 1000U BID | 80017748 | 90 | 180 |  | 0.09389 | 16.9002 | 9.100107692 | 0 | 26.00030769 | 9.100107692 | 0 | 26.00030769 |  |  |  |
| Newfoundland and Labrador/ Total Q1 |  |  |  |  |  |  |  |  |  |  | 119.7519004 | 136.815 | 118.3830972 |  |  |  |
| Nova Scotia | Metformin 1000mg BID | 2167786 | 90 | 360 | 0.0247 |  | 8.892 | 0.71136 | 11.95 | 21.55336 | 12.66136 | 15.087352 | 6.466008 |  |  |  |
| Nova Scotia | Atorvastatin 40mg daily | 2295296 | 90 | 90 | 0.2342 |  | 21.078 | 1.68624 | 11.95 | 34.71424 | 13.63624 | 24.299968 | 10.414272 |  | Nova Scotia /Annual costs |  |
| Nova Scotia | Omeprazole 20mg daily | 2245058 | 90 | 90 | 0.2287 |  | 20.583 | 1.64664 | 11.95 | 34.17964 | 13.59664 | 23.925748 | 10.253892 |  | Pharmacy Margin | 477.1111026 |
| Nova Scotia | Irbesartan/HCTZ 300 mg/25 mg daily | 2447894 | 90 | 90 | 0.2184 |  | 19.656 | 1.57248 | 11.95 | 33.17848 | 13.52248 | 23.224936 | 9.953544 |  | Government share | 596.9992826 |
| Nova Scotia | Levothyroxine 50 mcg daily | 2213192 | 90 | 90 |  | 0.0316 | 2.844 | 0.29862 | 11.95 | 15.09262 | 12.24862 | 10.564834 | 4.527786 |  | Patient share | 420.50422 |
| Nova Scotia | Atenolol 50mg daily | 2255545 | 90 | 90 | 0.1107 |  | 9.963 | 0.79704 | 11.95 | 22.71004 | 12.74704 | 15.897028 | 6.813012 |  |  |  |
| Nova Scotia | Levemir cartridge 100 unit / ml inj | 2271842 | 90 | 3 |  | 7.1013 | 21.3039 | 2.2369095 | 11.95 | 35.4908095 | 14.1869095 | 24.84356665 | 10.64724285 |  |  |  |
| Nova Scotia | Lorazepam 1mg QHS | 655759 | 90 | 90 | 0.0447 |  | 4.023 | 0.32184 | 11.95 | 16.29484 | 12.27184 | 11.406388 | 4.888452 |  |  |  |
| Nova Scotia | ASAEC 81mg daily | 2237726 | 90 | 90 |  | 0.1095 | 9.855 | 5.306538462 | 0 | 15.16153846 | 5.306538462 | 0 | 15.16153846 |  |  |  |
| Nova Scotia | Calcium 500mg / Vitamin D 1000U BID | 80017748 | 90 | 180 |  | 0.09389 | 16.9002 | 9.100107692 | 0 | 26.00030769 | 9.100107692 | 0 | 26.00030769 |  |  |  |
| Nova Scotia/ Total Q1 |  |  |  |  |  |  |  |  |  |  | 119.2777757 | 149.2498207 | 105.126055 |  |  |  |
| Ontario | Metformin 1000mg BID | 2167786 | 90 | 360 |  | 0.0247 | 8.892 | 0.71136 | 8.83 | 18.43336 | 9.54136 | 12.32336 | 6.11 |  |  |  |
| Ontario | Atorvastatin 40mg daily | 2295296 | 90 | 90 |  | 0.2342 | 21.078 | 1.68624 | 8.83 | 31.59424 | 10.51624 | 25.48424 | 6.11 |  | Ontario /Annual costs |  |
| Ontario | Omeprazole 20mg daily | 2245058 | 90 | 90 |  | 0.2287 | 20.583 | 1.64664 | 8.83 | 31.05964 | 10.47664 | 24.94964 | 6.11 |  | Pharmacy Margin | 388.9459446 |
| Ontario | Irbesartan/HCTZ 300 mg/25 mg daily | 2447894 | 90 | 90 |  | 0.2184 | 19.656 | 1.57248 | 8.83 | 30.05848 | 10.40248 | 23.94848 | 6.11 |  | Government share | 745.29136 |
| Ontario | Levothyroxine 50 mcg daily | 2213192 | 90 | 90 |  | 0.0316 | 2.844 | 0.22752 | 8.83 | 11.90152 | 9.05752 | 5.79152 | 6.11 |  | Patient share | 360.1673846 |
| Ontario | Atenolol 50mg daily | 2255545 | 90 | 90 |  | 0.1107 | 9.963 | 0.79704 | 8.83 | 19.59004 | 9.62704 | 13.48004 | 6.11 |  |  |  |
| Ontario | Levemir cartridge 100 unit / ml inj | 2271842 | 90 | 3 |  | 21.778 | 65.334 | 5.22672 | 8.83 | 79.39072 | 14.05672 | 73.28072 | 6.11 |  |  |  |
| Ontario | Lorazepam 1mg QHS | 655759 | 90 | 90 |  | 0.0447 | 4.023 | 0.32184 | 8.83 | 13.17484 | 9.15184 | 7.06484 | 6.11 |  |  |  |
| Ontario | ASAEC 81mg daily | 2237726 | 90 | 90 |  | 0.1095 | 9.855 | 5.306538462 | 0 | 15.16153846 | 5.306538462 | 0 | 15.16153846 |  |  |  |
| Ontario | Calcium 500mg / Vitamin D 1000U BID | 80017748 | 90 | 180 |  | 0.09389 | 16.9002 | 9.100107692 | 0 | 26.00030769 | 9.100107692 | 0 | 26.00030769 |  |  |  |
| Ontario/ Total Q1 |  |  |  |  |  |  |  |  |  |  | 97.23648615 | 186.32284 | 90.04184615 |  |  |  |
| Prince Edward Island | Metformin 1000mg BID | 2167786 | 90 | 360 | 0.0247 |  | 8.892 | 0.53352 | 12.36 | 21.78552 | 12.89352 | 5.84552 | 15.94 |  |  |  |
| Prince Edward Island | Atorvastatin 40mg daily | 2295296 | 90 | 90 | 0.2342 |  | 21.078 | 1.26468 | 12.36 | 34.70268 | 13.62468 | 18.76268 | 15.94 |  | Prince Edward Island / Annual costs |  |
| Prince Edward Island | Omeprazole 20mg daily | 2245058 | 90 | 90 | 0.2287 |  | 20.583 | 1.23498 | 12.36 | 34.17798 | 13.59498 | 18.23798 | 15.94 |  | Pharmacy Margin | 479.1359206 |
| Prince Edward Island | Irbesartan/HCTZ 300 mg/25 mg daily | 2447894 | 90 | 90 | 0.2184 |  | 19.656 | 1.17936 | 12.36 | 33.19536 | 13.53936 | 17.25536 | 15.94 |  | Government share | 307.3244 |
| Prince Edward Island | Levothyroxine 50 mcg daily | 2213192 | 90 | 90 |  | 0.031 | 2.79 | 0.1674 | 12.36 | 15.3174 | 12.5274 | 4.8374 | 10.48 |  | Patient share | 711.9879206 |
| Prince Edward Island | Atenolol 50mg daily | 2255545 | 90 | 90 | 0.1107 |  | 9.963 | 0.59778 | 12.36 | 22.92078 | 12.95778 | 6.98078 | 15.94 |  |  |  |
| Prince Edward Island | Levemir cartridge 100 unit / ml inj | 2271842 | 90 | 3 |  | 7.1013 | 21.3039 | 1.278234 | 12.36 | 34.942134 | 13.638234 | 0 | 34.942134 |  |  |  |
| Prince Edward Island | Lorazepam 1mg QHS | 655759 | 90 | 90 | 0.0447 |  | 4.023 | 0.24138 | 12.36 | 16.62438 | 12.60138 | 4.91138 | 11.713 |  |  |  |
| Prince Edward Island | ASAEC 81mg daily | 2237726 | 90 | 90 |  | 0.1095 | 9.855 | 5.306538462 | 0 | 15.16153846 | 5.306538462 | 0 | 15.16153846 |  |  |  |
| Prince Edward Island | Calcium 500mg / Vitamin D 1000U BID | 80017748 | 90 | 180 |  | 0.09389 | 16.9002 | 9.100107692 | 0 | 26.00030769 | 9.100107692 | 0 | 26.00030769 |  |  |  |
| Prince Edward Island/ Total Q1 |  |  |  |  |  |  |  |  |  |  | 119.7839802 | 76.8311 | 177.9969802 |  |  |  |
| Quebec | Metformin 1000mg BID | 2167786 | 90 | 360 | 0 | 0.0247 | 8.892 | 0.57798 | 27 | 36.46998 | 27.57798 | 23.74195698 | 12.72802302 |  |  |  |
| Quebec | Atorvastatin 40mg daily | 2295296 | 90 | 90 | 0 | 0.2342 | 21.078 | 1.37007 | 27 | 49.44807 | 28.37007 | 32.19069357 | 17.25737643 |  | Quebec /Annual costs |  |
| Quebec | Omeprazole 20mg daily | 2245058 | 90 | 90 | 0 | 0.2287 | 20.583 | 1.337895 | 27 | 48.920895 | 28.337895 | 31.84750265 | 17.07339236 |  | Pharmacy Margin | 959.5540846 |
| Quebec | Irbesartan/HCTZ 300 mg/25 mg daily | 2447894 | 90 | 90 | 0 | 0.2184 | 19.656 | 1.27764 | 27 | 47.93364 | 28.27764 | 31.20479964 | 16.72884036 |  | Government share | 967.0133025 |
| Quebec | Levothyroxine 50 mcg daily | 2213192 | 90 | 90 | 0 | 0.0274 | 2.466 | 0.16029 | 27 | 29.62629 | 27.16029 | 19.28671479 | 10.33957521 |  | Patient share | 683.0615821 |
| Quebec | Atenolol 50mg daily | 2255545 | 90 | 90 | 0 | 0.1107 | 9.963 | 0.647595 | 27 | 37.610595 | 27.647595 | 24.48449735 | 13.12609766 |  |  |  |
| Quebec | Levemir cartridge 100 unit / ml inj | 2271842 | 90 | 3 | 0 | 19.738 | 59.214 | 3.84891 | 27 | 90.06291 | 30.84891 | 58.63095441 | 31.43195559 |  |  |  |
| Quebec | Lorazepam 1mg QHS | 655759 | 90 | 90 | 0 | 0.0447 | 4.023 | 0.261495 | 27 | 31.284495 | 27.261495 | 20.36620625 | 10.91828876 |  |  |  |
| Quebec | ASAEC 81mg daily | 2237726 | 90 | 90 | 0 | 0.1095 | 9.855 | 5.306538462 | 0 | 15.16153846 | 5.306538462 | 0 | 15.16153846 |  |  |  |
| Quebec | Calcium 500mg / Vitamin D 1000U BID | 80017748 | 90 | 180 | 0 | 0.09389 | 16.9002 | 9.100107692 | 0 | 26.00030769 | 9.100107692 | 0 | 26.00030769 |  |  |  |
| Quebec/Total Q1 |  |  |  |  |  |  |  |  |  |  | 239.8885212 | 241.7533256 | 170.7653955 |  |  |  |
| Saskatchewan | Metformin 1000mg BID | 2167786 | 90 | 360 |  | 0.0247 | 8.892 | 1.3338 | 11.4 | 21.6258 | 12.7338 | 0 | 21.6258 |  |  |  |
| Saskatchewan | Atorvastatin 40mg daily | 2295296 | 90 | 90 |  | 0.2342 | 21.078 | 2.1078 | 11.4 | 34.5858 | 13.5078 | 9.5858 | 25 |  | Saskatchewan /Annual costs |  |
| Saskatchewan | Omeprazole 20mg daily | 2245058 | 90 | 90 |  | 0.2287 | 20.583 | 2.0583 | 11.4 | 34.0413 | 13.4583 | 9.0413 | 25 |  | Pharmacy Margin | 492.0715846 |
| Saskatchewan | Irbesartan/HCTZ 300 mg/25 mg daily | 2447894 | 90 | 90 |  | 0.2184 | 19.656 | 1.9656 | 11.4 | 33.0216 | 13.3656 | 8.0216 | 25 |  | Government share | 333.4076 |
| Saskatchewan | Levothyroxine 50 mcg daily | 2213192 | 90 | 90 |  | 0.0316 | 2.844 | 0.8532 | 11.4 | 15.0972 | 12.2532 | 0 | 15.0972 |  | Patient share | 869.4887846 |
| Saskatchewan | Atenolol 50mg daily | 2255545 | 90 | 90 |  | 0.1107 | 9.963 | 1.49445 | 11.4 | 22.85745 | 12.89445 | 0 | 22.85745 |  |  |  |
| Saskatchewan | Levemir cartridge 100 unit / ml inj | 2271842 | 90 | 3 |  | 21.304 | 63.912 | 6.3912 | 11.4 | 81.7032 | 17.7912 | 56.7032 | 25 |  |  |  |
| Saskatchewan | Lorazepam 1mg QHS | 655759 | 90 | 90 |  | 0.0447 | 4.023 | 1.2069 | 11.4 | 16.6299 | 12.6069 | 0 | 16.6299 |  |  |  |
| Saskatchewan | ASAEC 81mg daily | 2237726 | 90 | 90 |  | 0.1095 | 9.855 | 5.306538462 | 0 | 15.16153846 | 5.306538462 | 0 | 15.16153846 |  |  |  |
| Saskatchewan | Calcium 500mg / Vitamin D 1000U BID | 80017748 | 90 | 180 |  | 0.09389 | 16.9002 | 9.100107692 | 0 | 26.00030769 | 9.100107692 | 0 | 26.00030769 |  |  |  |
| Saskatchewan/ Total Q1 |  |  |  |  |  |  |  |  |  |  | 123.0178962 | 83.3519 | 217.3721962 |  |  |  |
| Northwest Territories | Metformin 1000mg BID | 2167786 | 90 | 360 |  | 0.0247 | 8.892 | 1.6325712 | 12.72 | 23.2445712 | 14.3525712 | 23.2445712 | 0 |  |  |  |
| Northwest Territories | Atorvastatin 40mg daily | 2295296 | 90 | 90 |  | 0.2342 | 21.078 | 3.8699208 | 12.72 | 37.6679208 | 16.5899208 | 37.6679208 | 0 |  | Northwest Territories /Annual costs |  |
| Northwest Territories | Omeprazole 20mg daily | 2245058 | 90 | 90 |  | 0.2287 | 20.583 | 3.7790388 | 12.72 | 37.0820388 | 16.4990388 | 37.0820388 | 0 |  | Pharmacy Margin | 544.1939528 |
| Northwest Territories | Irbesartan/HCTZ 300 mg/25 mg daily | 2447894 | 90 | 90 |  | 0.2184 | 19.656 | 3.6088416 | 12.72 | 35.9848416 | 16.3288416 | 35.9848416 | 0 |  | Government share | 919.7229682 |
| Northwest Territories | Levothyroxine 50 mcg daily | 2213192 | 90 | 90 |  | 0.031 | 2.79 | 0.512244 | 12.72 | 16.022244 | 13.232244 | 16.022244 | 0 |  | Patient share | 164.6473846 |
| Northwest Territories | Atenolol 50mg daily | 2255545 | 90 | 90 |  | 0.1107 | 9.963 | 1.8292068 | 12.72 | 24.5122068 | 14.5492068 | 24.5122068 | 0 |  |  |  |
| Northwest Territories | Levemir cartridge 100 unit / ml inj | 2271842 | 90 | 3 |  | 7.1013 | 21.3039 | 3.91139604 | 12.72 | 37.93529604 | 16.63139604 | 37.93529604 | 0 |  |  |  |
| Northwest Territories | Lorazepam 1mg QHS | 655759 | 90 | 90 |  | 0.0447 | 4.023 | 0.7386228 | 12.72 | 17.4816228 | 13.4586228 | 17.4816228 | 0 |  |  |  |
| Northwest Territories | ASAEC 81mg daily | 2237726 | 90 | 90 |  | 0.1095 | 9.855 | 5.306538462 | 0 | 15.16153846 | 5.306538462 | 0 | 15.16153846 |  |  |  |
| Northwest Territories | Calcium 500mg / Vitamin D 1000U BID | 80017748 | 90 | 180 |  | 0.09389 | 16.9002 | 9.100107692 | 0 | 26.00030769 | 9.100107692 | 0 | 26.00030769 |  |  |  |
| Northwest Territories |  |  |  |  |  |  |  |  |  |  | 136.0484882 | 229.930742 | 41.16184615 |  |  |  |
| Nunavut | Metformin 1000mg BID | 2167786 | 90 | 360 | 0 | 0.0247 | 8.892 | 2.1127392 | 16.95 | 27.9547392 | 19.0627392 | 27.9547392 | 0 |  |  |  |
| Nunavut | Atorvastatin 40mg daily | 2295296 | 90 | 90 | 0 | 0.2342 | 21.078 | 5.0081328 | 16.95 | 43.0361328 | 21.9581328 | 43.0361328 | 0 |  | Nunavut /Annual costs |  |
| Nunavut | Omeprazole 20mg daily | 2245058 | 90 | 90 | 0 | 0.2287 | 20.583 | 4.8905208 | 16.95 | 42.4235208 | 21.8405208 | 42.4235208 | 0 |  | Pharmacy Margin | 702.9443552 |
| Nunavut | Irbesartan/HCTZ 300 mg/25 mg daily | 2447894 | 90 | 90 | 0 | 0.2184 | 19.656 | 4.6702656 | 16.95 | 41.2762656 | 21.6202656 | 41.2762656 | 0 |  | Government share | 1078.473371 |
| Nunavut | Levothyroxine 50 mcg daily | 2213192 | 90 | 90 | 0 | 0.031 | 2.79 | 0.662904 | 16.95 | 20.402904 | 17.612904 | 20.402904 | 0 |  | Patient share | 164.6473846 |
| Nunavut | Atenolol 50mg daily | 2255545 | 90 | 90 | 0 | 0.1107 | 9.963 | 2.3672088 | 16.95 | 29.2802088 | 19.3172088 | 29.2802088 | 0 |  |  |  |
| Nunavut | Levemir cartridge 100 unit / ml inj | 2271842 | 90 | 3 |  | 7.1013 | 21.3039 | 5.06180664 | 16.95 | 43.31570664 | 22.01180664 | 43.31570664 | 0 |  |  |  |
| Nunavut | Lorazepam 1mg QHS | 655759 | 90 | 90 | 0 | 0.0447 | 4.023 | 0.9558648 | 16.95 | 21.9288648 | 17.9058648 | 21.9288648 | 0 |  |  |  |
| Nunavut | ASAEC 81mg daily | 2237726 | 90 | 90 | 0 | 0.1095 | 9.855 | 5.306538462 | 0 | 15.16153846 | 5.306538462 | 0 | 15.16153846 |  |  |  |
| Nunavut | Calcium 500mg / Vitamin D 1000U BID | 80017748 | 90 | 180 | 0 | 0.09389 | 16.9002 | 9.100107692 | 0 | 26.00030769 | 9.100107692 | 0 | 26.00030769 |  |  |  |
| Nunavut |  |  |  |  |  |  |  |  |  |  | 175.7360888 | 269.6183426 | 41.16184615 |  |  |  |
| Yukon | Metformin 1000mg BID | 2167786 | 90 | 360 |  | 0.0247 | 8.892 | 2.1127392 | 12.72 | 23.7247392 | 14.8327392 | 23.7247392 | 0 |  |  |  |
| Yukon | Atorvastatin 40mg daily | 2295296 | 90 | 90 |  | 0.2342 | 21.078 | 5.0081328 | 12.72 | 38.8061328 | 17.7281328 | 38.8061328 | 0 |  | Yukon /Annual costs |  |
| Yukon | Omeprazole 20mg daily | 2245058 | 90 | 90 |  | 0.2287 | 20.583 | 4.8905208 | 12.72 | 38.1935208 | 17.6105208 | 38.1935208 | 0 |  | Pharmacy Margin | 602.6774356 |
| Yukon | Irbesartan/HCTZ 300 mg/25 mg daily | 2357410 | 90 | 90 |  | 0.2184 | 19.656 | 4.6702656 | 12.72 | 37.0462656 | 17.3902656 | 37.0462656 | 0 |  | Government share | 1022.325005 |
| Yukon | Levothyroxine 50 mcg daily | 2213192 | 90 | 90 |  | 0.03 | 2.7 | 0.64152 | 12.72 | 16.06152 | 13.36152 | 16.06152 | 0 |  | Patient share | 104.0012308 |
| Yukon | Atenolol 50mg daily | 2255545 | 90 | 90 |  | 0.1107 | 9.963 | 2.3672088 | 12.72 | 25.0502088 | 15.0872088 | 25.0502088 | 0 |  |  |  |
| Yukon | Levemir cartridge 100 unit / ml inj | 2271842 | 90 | 3 |  | 6.78 | 20.34 | 4.832784 | 12.72 | 37.892784 | 17.552784 | 37.892784 | 0 |  |  |  |
| Yukon | Lorazepam 1mg QHS | 655759 | 90 | 90 |  | 0.04 | 3.6 | 0.85536 | 12.72 | 17.17536 | 13.57536 | 17.17536 | 0 |  |  |  |
| Yukon | ASAEC 81mg daily | 2237726 | 90 | 90 |  | 0.08 | 7.2 | 1.71072 | 12.72 | 21.63072 | 14.43072 | 21.63072 | 0 |  |  |  |
| Yukon | Calcium 500mg / Vitamin D 1000U BID | 80017748 | 90 | 180 |  | 0.09389 | 16.9002 | 9.100107692 | 0 | 26.00030769 | 9.100107692 | 0 | 26.00030769 |  |  |  |
| Yukon |  |  |  |  |  |  |  |  |  |  | 150.6693589 | 255.5812512 | 26.00030769 |  |  |  |

Supplementary Table 9 – Scenario 8

| Province | Drug | DIN | Days Supply | Quantity | MAC/Unit | Drug Cost / Unit | Drug Cost | Markup | Dispensing Fee | Total Cost | Pharmacy Margin $ | Government Share | Patient Share |  |  |  |
| --- | --- | --- | --- | --- | --- | --- | --- | --- | --- | --- | --- | --- | --- | --- | --- | --- |
| Alberta | Metformin 1000mg BID | 2167786 | 90 | 360 |  | 0.0247 | 8.892 | 0.9078732 | 12.15 | 21.9498732 | 13.0578732 | 15.36491124 | 6.58496196 |  |  |  |
| Alberta | Atorvastatin 40mg daily | 2295296 | 90 | 90 |  | 0.2342 | 21.078 | 2.1520638 | 12.15 | 35.3800638 | 14.3020638 | 24.76604466 | 10.61401914 |  | Alberta /Annual costs |  |
| Alberta | Omeprazole 20mg daily | 2245058 | 90 | 90 |  | 0.2287 | 20.583 | 2.1015243 | 12.15 | 34.8345243 | 14.2515243 | 24.38416701 | 10.45035729 |  | Pharmacy Margin | 964.8452227 |
| Alberta | Irbesartan 300 mg daily | 2406128 | 90 | 90 |  | 0.2281 | 20.529 | 2.0960109 | 12.15 | 34.7750109 | 14.2460109 | 24.34250763 | 10.43250327 |  | Government share | 509.259245 |
| Alberta | Levothyroxine 50 mcg daily | 2213192 | 90 | 90 |  | 0.031 | 2.79 | 0.284859 | 12.15 | 15.224859 | 12.434859 | 10.6574013 | 4.5674577 |  | Patient share | 4125.602378 |
| Alberta | Atenolol 50mg daily | 2255545 | 90 | 90 |  | 0.1107 | 9.963 | 1.0172223 | 12.15 | 23.1302223 | 13.1672223 | 16.19115561 | 6.93906669 |  |  |  |
| Alberta | Liraglutide inj 1.8mg daily | 2351064 | 90 | 27 |  | 29.7367 | 802.8909 | 120.6343577 | 12.15 | 935.6752577 | 132.7843577 | 0 | 935.6752577 |  |  |  |
| Alberta | Lorazepam 1mg QHS | 655759 | 90 | 90 |  | 0.0447 | 4.023 | 0.4107483 | 12.15 | 16.5837483 | 12.5607483 | 11.60862381 | 4.97512449 |  |  |  |
| Alberta | ASAEC 81mg daily | 2237726 | 90 | 90 |  | 0.1095 | 9.855 | 5.306538462 | 0 | 15.16153846 | 5.306538462 | 0 | 15.16153846 |  |  |  |
| Alberta | Calcium 500mg / Vitamin D 1000U BID | 80017748 | 90 | 180 |  | 0.09389 | 16.9002 | 9.100107692 | 0 | 26.00030769 | 9.100107692 | 0 | 26.00030769 |  |  |  |
| Alberta/ Total Q1 |  |  |  |  |  |  |  |  |  |  | 241.2113057 | 127.3148113 | 1031.400594 |  |  |  |
| British Columbia | Metformin 1000mg BID | 2167786 | 90 | 360 | 0.0267 |  | 8.9 | 0.712 | 10 | 19.612 | 10.712 | 0 | 19.612 |  |  |  |
| British Columbia | Atorvastatin 40mg daily | 2295296 | 90 | 90 | 0.2529 |  | 21.075 | 1.686 | 10 | 32.761 | 11.686 | 0 | 32.761 |  | British Columbia /Annual costs |  |
| British Columbia | Omeprazole 20mg daily | 2245058 | 90 | 90 | 0.2025 |  | 16.875 | 1.35 | 10 | 28.225 | 11.35 | 0 | 28.225 |  | Pharmacy Margin | 683.7148521 |
| British Columbia | Irbesartan 300 mg daily | 2406128 | 90 | 90 | 0.2719 |  | 22.65833 | 1.812666667 | 10 | 34.471 | 11.81266667 | 0 | 34.471 |  | Government share | 0 |
| British Columbia | Levothyroxine 50 mcg daily | 2213192 | 90 | 90 | 0.0341 |  | 2.841667 | 0.227333333 | 10 | 13.069 | 10.22733333 | 0 | 13.069 |  | Patient share | 4342.745919 |
| British Columbia | Atenolol 50mg daily | 2255545 | 90 | 90 | 0.1196 |  | 9.966667 | 0.797333333 | 10 | 20.764 | 10.79733333 | 0 | 20.764 |  |  |  |
| British Columbia | Liraglutide inj 1.8mg daily | 2351064 | 90 | 27 |  | 29.7367 | 802.8909 | 64.231272 | 10 | 877.122172 | 74.231272 | 0 | 877.122172 |  |  |  |
| British Columbia | Lorazepam 1mg QHS | 655759 | 90 | 90 | 0.0483 |  | 4.025 | 0.322 | 10 | 14.347 | 10.322 | 0 | 14.347 |  |  |  |
| British Columbia | ASAEC 81mg daily | 2237726 | 90 | 90 | 0.1035 |  | 8.625 | 0.69 | 10 | 19.315 | 10.69 | 0 | 19.315 |  |  |  |
| British Columbia | Calcium 500mg / Vitamin D 1000U BID | 80017748 | 90 | 180 |  | 0.09389 | 16.9002 | 9.100107692 | 0 | 26.00030769 | 9.100107692 | 0 | 26.00030769 |  |  |  |
| British Columbia/ Total Q1 |  |  |  |  |  |  |  |  |  |  | 170.928713 | 0 | 1085.68648 |  |  |  |
| Manitoba | Metformin 1000mg BID | 2167786 | 90 | 360 |  | 0.0259 | 9.324 | 0 | 13.65 | 22.974 | 13.65 | 22.974 | 0 |  |  |  |
| Manitoba | Atorvastatin 40mg daily | 2295296 | 90 | 90 |  | 0.2459 | 22.131 | 0 | 13.65 | 35.781 | 13.65 | 35.781 | 0 |  | Manitoba /Annual costs |  |
| Manitoba | Omeprazole 20mg daily | 2245058 | 90 | 90 |  | 0.2401 | 21.609 | 0 | 13.65 | 35.259 | 13.65 | 35.259 | 0 |  | Pharmacy Margin | 494.4265846 |
| Manitoba | Irbesartan 300 mg daily | 2406128 | 90 | 90 |  | 0.2395 | 21.555 | 0 | 13.65 | 35.205 | 13.65 | 35.205 | 0 |  | Government share | 751.38 |
| Manitoba | Levothyroxine 50 mcg daily | 2213192 | 90 | 90 |  | 0.031 | 2.79 | 0 | 13.65 | 16.44 | 13.65 | 16.44 | 0 |  | Patient share | 3430.810985 |
| Manitoba | Atenolol 50mg daily | 2255545 | 90 | 90 |  | 0.1162 | 10.458 | 0 | 13.65 | 24.108 | 13.65 | 24.108 | 0 |  |  |  |
| Manitoba | Liraglutide inj 1.8mg daily | 2351064 | 90 | 27 |  | 29.7367 | 802.8909 | 0 | 13.65 | 816.5409 | 13.65 | 0 | 816.5409 |  |  |  |
| Manitoba | Lorazepam 1mg QHS | 655759 | 90 | 90 |  | 0.0492 | 4.428 | 0 | 13.65 | 18.078 | 13.65 | 18.078 | 0 |  |  |  |
| Manitoba | ASAEC 81mg daily | 2237726 | 90 | 90 |  | 0.1095 | 9.855 | 5.306538462 | 0 | 15.16153846 | 5.306538462 | 0 | 15.16153846 |  |  |  |
| Manitoba | Calcium 500mg / Vitamin D 1000U BID | 80017748 | 90 | 180 |  | 0.09389 | 16.9002 | 9.100107692 | 0 | 26.00030769 | 9.100107692 | 0 | 26.00030769 |  |  |  |
| Manitoba/ Total Q1 |  |  |  |  |  |  |  |  |  |  | 123.6066462 | 187.845 | 857.7027462 |  |  |  |
| New Brunswick | Metformin 1000mg BID | 2167786 | 90 | 360 | 0.0247 |  | 8.892 | 0.71136 | 11 | 20.60336 | 11.71136 | 14.422352 | 6.181008 |  |  |  |
| New Brunswick | Atorvastatin 40mg daily | 2295296 | 90 | 90 | 0.2342 |  | 21.078 | 1.68624 | 11 | 33.76424 | 12.68624 | 23.634968 | 10.129272 |  | New Brunswick /Annual costs |  |
| New Brunswick | Omeprazole 20mg daily | 2245058 | 90 | 90 | 0.2287 |  | 20.583 | 1.64664 | 11 | 33.22964 | 12.64664 | 23.260748 | 9.968892 |  | Pharmacy Margin | 693.7734326 |
| New Brunswick | Irbesartan 300 mg daily | 2406128 | 90 | 90 | 0.2281 |  | 20.529 | 1.64232 | 11 | 33.17132 | 12.64232 | 23.219924 | 9.951396 |  | Government share | 480.906832 |
| New Brunswick | Levothyroxine 50 mcg daily | 2213192 | 90 | 90 |  | 0.0311 | 2.879 | 0 | 11 | 13.879 | 11 | 9.7153 | 4.1637 |  | Patient share | 3883.239001 |
| New Brunswick | Atenolol 50mg daily | 2255545 | 90 | 90 | 0.1107 |  | 9.963 | 0.79704 | 11 | 21.76004 | 11.79704 | 15.232028 | 6.528012 |  |  |  |
| New Brunswick | Liraglutide inj 1.8mg daily | 2351064 | 90 | 27 |  | 29.7367 | 802.8909 | 64.231272 | 11 | 878.122172 | 75.231272 | 0 | 878.122172 |  |  |  |
| New Brunswick | Lorazepam 1mg QHS | 655759 | 90 | 90 | 0.0447 | 0 | 4.023 | 0.32184 | 11 | 15.34484 | 11.32184 | 10.741388 | 4.603452 |  |  |  |
| New Brunswick | ASAEC 81mg daily | 2237726 | 90 | 90 |  | 0.1095 | 9.855 | 5.306538462 | 0 | 15.16153846 | 5.306538462 | 0 | 15.16153846 |  |  |  |
| New Brunswick | Calcium 500mg / Vitamin D 1000U BID | 80017748 | 90 | 180 |  | 0.09389 | 16.9002 | 9.100107692 | 0 | 26.00030769 | 9.100107692 | 0 | 26.00030769 |  |  |  |
| New Brunswick/ Total Q1 |  |  |  |  |  |  |  |  |  |  | 173.4433582 | 120.226708 | 970.8097502 |  |  |  |
| Newfoundland and Labrador | Metformin 1000mg BID | 2167786 | 90 | 360 |  | 0.0269 | 8.925346 | 0.758654378 | 12 | 21.684 | 12.75865438 | 15.684 | 6 |  |  |  |
| Newfoundland and Labrador | Atorvastatin 40mg daily | 2295296 | 90 | 90 |  | 0.2553 | 21.17696 | 1.800041475 | 12 | 34.977 | 13.80004147 | 28.977 | 6 |  | Newfoundland and Labrador / Annual costs |  |
| Newfoundland and Labrador | Omeprazole 20mg daily | 2245058 | 90 | 90 |  | 0.2493 | 20.67926 | 1.757737327 | 12 | 34.437 | 13.75773733 | 28.437 | 6 |  | Pharmacy Margin | 872.6750506 |
| Newfoundland and Labrador | Irbesartan 300 mg daily | 2406128 | 90 | 90 |  | 0.2486 | 20.6212 | 1.752801843 | 12 | 34.374 | 13.75280184 | 28.374 | 6 |  | Government share | 551.04 |
| Newfoundland and Labrador | Levothyroxine 50 mcg daily | 2213192 | 90 | 90 |  | 0.0338 | 2.803687 | 0.238313364 | 12 | 15.042 | 12.23831336 | 9.042 | 6 |  | Patient share | 3993.251709 |
| Newfoundland and Labrador | Atenolol 50mg daily | 2255545 | 90 | 90 |  | 0.1207 | 10.01198 | 0.851018433 | 12 | 22.863 | 12.85101843 | 16.863 | 6 |  |  |  |
| Newfoundland and Labrador | Liraglutide inj 1.8mg daily | 2351064 | 90 | 27 |  | 29.7367 | 802.8909 | 72.260181 | 40 | 915.151081 | 112.260181 | 0 | 915.151081 |  |  |  |
| Newfoundland and Labrador | Lorazepam 1mg QHS | 655759 | 90 | 90 |  | 0.0487 | 4.039631 | 0.343368664 | 12 | 16.383 | 12.34336866 | 10.383 | 6 |  |  |  |
| Newfoundland and Labrador | ASAEC 81mg daily | 2237726 | 90 | 90 |  | 0.1095 | 9.855 | 5.306538462 | 0 | 15.16153846 | 5.306538462 | 0 | 15.16153846 |  |  |  |
| Newfoundland and Labrador | Calcium 500mg / Vitamin D 1000U BID | 80017748 | 90 | 180 |  | 0.09389 | 16.9002 | 9.100107692 | 0 | 26.00030769 | 9.100107692 | 0 | 26.00030769 |  |  |  |
| Newfoundland and Labrador/ Total Q1 |  |  |  |  |  |  |  |  |  |  | 218.1687626 | 137.76 | 998.3129272 |  |  |  |
| Nova Scotia | Metformin 1000mg BID | 2167786 | 90 | 360 | 0.0247 |  | 8.892 | 0.71136 | 11.95 | 21.55336 | 12.66136 | 15.087352 | 6.466008 |  |  |  |
| Nova Scotia | Atorvastatin 40mg daily | 2295296 | 90 | 90 | 0.2342 |  | 21.078 | 1.68624 | 11.95 | 34.71424 | 13.63624 | 24.299968 | 10.414272 |  | Nova Scotia /Annual costs |  |
| Nova Scotia | Omeprazole 20mg daily | 2245058 | 90 | 90 | 0.2287 |  | 20.583 | 1.64664 | 11.95 | 34.17964 | 13.59664 | 23.925748 | 10.253892 |  | Pharmacy Margin | 805.6570026 |
| Nova Scotia | Irbesartan 300 mg daily | 2406128 | 90 | 90 | 0.2281 |  | 20.529 | 1.64232 | 11.95 | 34.12132 | 13.59232 | 23.884924 | 10.236396 |  | Government share | 500.264968 |
| Nova Scotia | Levothyroxine 50 mcg daily | 2213192 | 90 | 90 |  | 0.0316 | 2.844 | 0.29862 | 11.95 | 15.09262 | 12.24862 | 10.564834 | 4.527786 |  | Patient share | 3975.624435 |
| Nova Scotia | Atenolol 50mg daily | 2255545 | 90 | 90 | 0.1107 |  | 9.963 | 0.79704 | 11.95 | 22.71004 | 12.74704 | 15.897028 | 6.813012 |  |  |  |
| Nova Scotia | Liraglutide inj 1.8mg daily | 2351064 | 90 | 27 |  | 29.7367 | 802.8909 | 84.3035445 | 11.95 | 899.1444445 | 96.2535445 | 0 | 899.1444445 |  |  |  |
| Nova Scotia | Lorazepam 1mg QHS | 655759 | 90 | 90 | 0.0447 |  | 4.023 | 0.32184 | 11.95 | 16.29484 | 12.27184 | 11.406388 | 4.888452 |  |  |  |
| Nova Scotia | ASAEC 81mg daily | 2237726 | 90 | 90 |  | 0.1095 | 9.855 | 5.306538462 | 0 | 15.16153846 | 5.306538462 | 0 | 15.16153846 |  |  |  |
| Nova Scotia | Calcium 500mg / Vitamin D 1000U BID | 80017748 | 90 | 180 |  | 0.09389 | 16.9002 | 9.100107692 | 0 | 26.00030769 | 9.100107692 | 0 | 26.00030769 |  |  |  |
| Nova Scotia/ Total Q1 |  |  |  |  |  |  |  |  |  |  | 201.4142507 | 125.066242 | 993.9061087 |  |  |  |
| Ontario | Metformin 1000mg BID | 2167786 | 90 | 360 |  | 0.0247 | 8.892 | 0.71136 | 8.83 | 18.43336 | 9.54136 | 12.32336 | 6.11 |  |  |  |
| Ontario | Atorvastatin 40mg daily | 2295296 | 90 | 90 |  | 0.2342 | 21.078 | 1.68624 | 8.83 | 31.59424 | 10.51624 | 25.48424 | 6.11 |  | Ontario /Annual costs |  |
| Ontario | Omeprazole 20mg daily | 2245058 | 90 | 90 |  | 0.2287 | 20.583 | 1.64664 | 8.83 | 31.05964 | 10.47664 | 24.94964 | 6.11 |  | Pharmacy Margin | 625.2435126 |
| Ontario | Irbesartan 300 mg daily | 2406128 | 90 | 90 |  | 0.2281 | 20.529 | 1.64232 | 8.83 | 31.00132 | 10.47232 | 24.89132 | 6.11 |  | Government share | 455.93984 |
| Ontario | Levothyroxine 50 mcg daily | 2213192 | 90 | 90 |  | 0.0316 | 2.844 | 0.22752 | 8.83 | 11.90152 | 9.05752 | 5.79152 | 6.11 |  | Patient share | 3839.536073 |
| Ontario | Atenolol 50mg daily | 2255545 | 90 | 90 |  | 0.1107 | 9.963 | 0.79704 | 8.83 | 19.59004 | 9.62704 | 13.48004 | 6.11 |  |  |  |
| Ontario | Liraglutide inj 1.8mg daily | 2351064 | 90 | 27 |  | 29.7367 | 802.8909 | 64.231272 | 8.83 | 875.952172 | 73.061272 | 0 | 875.952172 |  |  |  |
| Ontario | Lorazepam 1mg QHS | 655759 | 90 | 90 |  | 0.0447 | 4.023 | 0.32184 | 8.83 | 13.17484 | 9.15184 | 7.06484 | 6.11 |  |  |  |
| Ontario | ASAEC 81mg daily | 2237726 | 90 | 90 |  | 0.1095 | 9.855 | 5.306538462 | 0 | 15.16153846 | 5.306538462 | 0 | 15.16153846 |  |  |  |
| Ontario | Calcium 500mg / Vitamin D 1000U BID | 80017748 | 90 | 180 |  | 0.09389 | 16.9002 | 9.100107692 | 0 | 26.00030769 | 9.100107692 | 0 | 26.00030769 |  |  |  |
| Ontario/ Total Q1 |  |  |  |  |  |  |  |  |  |  | 156.3108782 | 113.98496 | 959.8840182 |  |  |  |
| Prince Edward Island | Metformin 1000mg BID | 2167786 | 90 | 360 | 0.0247 |  | 8.892 | 0.53352 | 12.36 | 21.78552 | 12.89352 | 5.84552 | 15.94 |  |  |  |
| Prince Edward Island | Atorvastatin 40mg daily | 2295296 | 90 | 90 | 0.2342 |  | 21.078 | 1.26468 | 12.36 | 34.70268 | 13.62468 | 18.76268 | 15.94 |  | Prince Edward Island / Annual costs |  |
| Prince Edward Island | Omeprazole 20mg daily | 2245058 | 90 | 90 | 0.2287 |  | 20.583 | 1.23498 | 12.36 | 34.17798 | 13.59498 | 18.23798 | 15.94 |  | Pharmacy Margin | 666.9263206 |
| Prince Edward Island | Irbesartan 300 mg daily | 2406128 | 90 | 90 | 0.2281 |  | 20.529 | 1.23174 | 12.36 | 34.12074 | 13.59174 | 18.18074 | 15.94 |  | Government share | 311.02592 |
| Prince Edward Island | Levothyroxine 50 mcg daily | 2213192 | 90 | 90 |  | 0.031 | 2.79 | 0.1674 | 12.36 | 15.3174 | 12.5274 | 4.8374 | 10.48 |  | Patient share | 4025.916801 |
| Prince Edward Island | Atenolol 50mg daily | 2255545 | 90 | 90 | 0.1107 |  | 9.963 | 0.59778 | 12.36 | 22.92078 | 12.95778 | 6.98078 | 15.94 |  |  |  |
| Prince Edward Island | Liraglutide inj 1.8mg daily | 2351064 | 90 | 27 |  | 29.7367 | 802.8909 | 48.173454 | 12.36 | 863.424354 | 60.533454 | 0 | 863.424354 |  |  |  |
| Prince Edward Island | Lorazepam 1mg QHS | 655759 | 90 | 90 | 0.0447 |  | 4.023 | 0.24138 | 12.36 | 16.62438 | 12.60138 | 4.91138 | 11.713 |  |  |  |
| Prince Edward Island | ASAEC 81mg daily | 2237726 | 90 | 90 |  | 0.1095 | 9.855 | 5.306538462 | 0 | 15.16153846 | 5.306538462 | 0 | 15.16153846 |  |  |  |
| Prince Edward Island | Calcium 500mg / Vitamin D 1000U BID | 80017748 | 90 | 180 |  | 0.09389 | 16.9002 | 9.100107692 | 0 | 26.00030769 | 9.100107692 | 0 | 26.00030769 |  |  |  |
| Prince Edward Island/ Total Q1 |  |  |  |  |  |  |  |  |  |  | 166.7315802 | 77.75648 | 1006.4792 |  |  |  |
| Quebec | Metformin 1000mg BID | 2167786 | 90 | 360 |  | 0.0247 | 8.892 | 0.57798 | 27 | 36.46998 | 27.57798 | 23.74195698 | 12.72802302 |  |  |  |
| Quebec | Atorvastatin 40mg daily | 2295296 | 90 | 90 |  | 0.2342 | 21.078 | 1.37007 | 27 | 49.44807 | 28.37007 | 32.19069357 | 17.25737643 |  | Quebec /Annual costs |  |
| Quebec | Omeprazole 20mg daily | 2245058 | 90 | 90 |  | 0.2287 | 20.583 | 1.337895 | 27 | 48.920895 | 28.337895 | 31.84750265 | 17.07339236 |  | Pharmacy Margin | 1104.652025 |
| Quebec | Irbesartan 300 mg daily | 2406128 | 90 | 90 |  | 0.2281 | 20.529 | 1.334385 | 27 | 48.863385 | 28.334385 | 31.81006364 | 17.05332137 |  | Government share | 2514.683737 |
| Quebec | Levothyroxine 50 mcg daily | 2213192 | 90 | 90 |  | 0.0274 | 2.466 | 0.16029 | 27 | 29.62629 | 27.16029 | 19.28671479 | 10.33957521 |  | Patient share | 1512.765087 |
| Quebec | Atenolol 50mg daily | 2255545 | 90 | 90 |  | 0.1107 | 9.963 | 0.647595 | 27 | 37.610595 | 27.647595 | 24.48449735 | 13.12609766 |  |  |  |
| Quebec | Liraglutide inj 1.8mg daily | 2351064 | 90 | 27 |  | 22.83 | 616.41 | 40.06665 | 27 | 683.47665 | 67.06665 | 444.9432992 | 238.5333509 |  |  |  |
| Quebec | Lorazepam 1mg QHS | 655759 | 90 | 90 |  | 0.0447 | 4.023 | 0.261495 | 27 | 31.284495 | 27.261495 | 20.36620625 | 10.91828876 |  |  |  |
| Quebec | ASAEC 81mg daily | 2237726 | 90 | 90 |  | 0.1095 | 9.855 | 5.306538462 | 0 | 15.16153846 | 5.306538462 | 0 | 15.16153846 |  |  |  |
| Quebec | Calcium 500mg / Vitamin D 1000U BID | 80017748 | 90 | 180 |  | 0.09389 | 16.9002 | 9.100107692 | 0 | 26.00030769 | 9.100107692 | 0 | 26.00030769 |  |  |  |
| Quebec/Total Q1 |  |  |  |  |  |  |  |  |  |  | 276.1630062 | 628.6709344 | 378.1912718 |  |  |  |
| Saskatchewan | Metformin 1000mg BID | 2167786 | 90 | 360 |  | 0.0247 | 8.892 | 1.3338 | 11.4 | 21.6258 | 12.7338 | 0 | 21.6258 |  |  |  |
| Saskatchewan | Atorvastatin 40mg daily | 2295296 | 90 | 90 |  | 0.2342 | 21.078 | 2.1078 | 11.4 | 34.5858 | 13.5078 | 9.5858 | 25 |  | Saskatchewan /Annual costs |  |
| Saskatchewan | Omeprazole 20mg daily | 2245058 | 90 | 90 |  | 0.2287 | 20.583 | 2.0583 | 11.4 | 34.0413 | 13.4583 | 9.0413 | 25 |  | Pharmacy Margin | 546.8559846 |
| Saskatchewan | Irbesartan 300 mg daily | 2406128 | 90 | 90 |  | 0.2281 | 20.529 | 2.0529 | 11.4 | 33.9819 | 13.4529 | 8.9819 | 25 |  | Government share | 110.436 |
| Saskatchewan | Levothyroxine 50 mcg daily | 2213192 | 90 | 90 |  | 0.0316 | 2.844 | 0.8532 | 11.4 | 15.0972 | 12.2532 | 0 | 15.0972 |  | Patient share | 4106.652385 |
| Saskatchewan | Atenolol 50mg daily | 2255545 | 90 | 90 |  | 0.1107 | 9.963 | 1.49445 | 11.4 | 22.85745 | 12.89445 | 0 | 22.85745 |  |  |  |
| Saskatchewan | Liraglutide inj 1.8mg daily | 2351064 | 90 | 27 |  | 29.7367 | 802.8909 | 20 | 11.4 | 834.2909 | 31.4 | 0 | 834.2909 |  |  |  |
| Saskatchewan | Lorazepam 1mg QHS | 655759 | 90 | 90 |  | 0.0447 | 4.023 | 1.2069 | 11.4 | 16.6299 | 12.6069 | 0 | 16.6299 |  |  |  |
| Saskatchewan | ASAEC 81mg daily | 2237726 | 90 | 90 |  | 0.1095 | 9.855 | 5.306538462 | 0 | 15.16153846 | 5.306538462 | 0 | 15.16153846 |  |  |  |
| Saskatchewan | Calcium 500mg / Vitamin D 1000U BID | 80017748 | 90 | 180 |  | 0.09389 | 16.9002 | 9.100107692 | 0 | 26.00030769 | 9.100107692 | 0 | 26.00030769 |  |  |  |
| Saskatchewan/ Total Q1 |  |  |  |  |  |  |  |  |  |  | 136.7139962 | 27.609 | 1026.663096 |  |  |  |
| Northwest Territories | Metformin 1000mg BID | 2167786 | 90 | 360 |  | 0.0247 | 8.892 | 1.6325712 | 12.72 | 23.2445712 | 14.3525712 | 23.2445712 | 0 |  |  |  |
| Northwest Territories | Atorvastatin 40mg daily | 2295296 | 90 | 90 |  | 0.2342 | 21.078 | 3.8699208 | 12.72 | 37.6679208 | 16.5899208 | 37.6679208 | 0 |  | Northwest Territories /Annual costs |  |
| Northwest Territories | Omeprazole 20mg daily | 2245058 | 90 | 90 |  | 0.2287 | 20.583 | 3.7790388 | 12.72 | 37.0820388 | 16.4990388 | 37.0820388 | 0 |  | Pharmacy Margin | 729.1894998 |
| Northwest Territories | Irbesartan 300 mg daily | 2406128 | 90 | 90 |  | 0.2281 | 20.529 | 3.7691244 | 12.72 | 37.0181244 | 16.4891244 | 37.0181244 | 0 |  | Government share | 772.1149152 |
| Northwest Territories | Levothyroxine 50 mcg daily | 2213192 | 90 | 90 |  | 0.031 | 2.79 | 0.512244 | 12.72 | 16.022244 | 13.232244 | 16.022244 | 0 |  | Patient share | 3627.090985 |
| Northwest Territories | Atenolol 50mg daily | 2255545 | 90 | 90 |  | 0.1107 | 9.963 | 1.8292068 | 12.72 | 24.5122068 | 14.5492068 | 24.5122068 | 0 |  |  |  |
| Northwest Territories | Liraglutide inj 1.8mg daily | 2351064 | 90 | 27 |  | 29.7367 | 802.8909 | 50 | 12.72 | 865.6109 | 62.72 | 0 | 865.6109 |  |  |  |
| Northwest Territories | Lorazepam 1mg QHS | 655759 | 90 | 90 |  | 0.0447 | 4.023 | 0.7386228 | 12.72 | 17.4816228 | 13.4586228 | 17.4816228 | 0 |  |  |  |
| Northwest Territories | ASAEC 81mg daily | 2237726 | 90 | 90 |  | 0.1095 | 9.855 | 5.306538462 | 0 | 15.16153846 | 5.306538462 | 0 | 15.16153846 |  |  |  |
| Northwest Territories | Calcium 500mg / Vitamin D 1000U BID | 80017748 | 90 | 180 |  | 0.09389 | 16.9002 | 9.100107692 | 0 | 26.00030769 | 9.100107692 | 0 | 26.00030769 |  |  |  |
| Northwest Territories |  |  |  |  |  |  |  |  |  |  | 182.297375 | 193.0287288 | 906.7727462 |  |  |  |
| Nunavut | Metformin 1000mg BID | 2167786 | 90 | 360 | 0 | 0.0247 | 8.892 | 2.1127392 | 16.95 | 27.9547392 | 19.0627392 | 27.9547392 | 0 |  |  |  |
| Nunavut | Atorvastatin 40mg daily | 2295296 | 90 | 90 | 0 | 0.2342 | 21.078 | 5.0081328 | 16.95 | 43.0361328 | 21.9581328 | 43.0361328 | 0 |  | Nunavut /Annual costs |  |
| Nunavut | Omeprazole 20mg daily | 2245058 | 90 | 90 | 0 | 0.2287 | 20.583 | 4.8905208 | 16.95 | 42.4235208 | 21.8405208 | 42.4235208 | 0 |  | Pharmacy Margin | 883.5268278 |
| Nunavut | Irbesartan 300 mg daily | 2406128 | 90 | 90 |  | 0.2281 | 20.529 | 4.8776904 | 16.95 | 42.3566904 | 21.8276904 | 42.3566904 | 0 |  | Government share | 909.5322432 |
| Nunavut | Levothyroxine 50 mcg daily | 2213192 | 90 | 90 | 0 | 0.031 | 2.79 | 0.662904 | 16.95 | 20.402904 | 17.612904 | 20.402904 | 0 |  | Patient share | 3644.010985 |
| Nunavut | Atenolol 50mg daily | 2255545 | 90 | 90 | 0 | 0.1107 | 9.963 | 2.3672088 | 16.95 | 29.2802088 | 19.3172088 | 29.2802088 | 0 |  |  |  |
| Nunavut | Liraglutide inj 1.8mg daily | 2351064 | 90 | 27 | 0 | 29.7367 | 802.8909 | 50 | 16.95 | 869.8409 | 66.95 | 0 | 869.8409 |  |  |  |
| Nunavut | Lorazepam 1mg QHS | 655759 | 90 | 90 | 0 | 0.0447 | 4.023 | 0.9558648 | 16.95 | 21.9288648 | 17.9058648 | 21.9288648 | 0 |  |  |  |
| Nunavut | ASAEC 81mg daily | 2237726 | 90 | 90 | 0 | 0.1095 | 9.855 | 5.306538462 | 0 | 15.16153846 | 5.306538462 | 0 | 15.16153846 |  |  |  |
| Nunavut | Calcium 500mg / Vitamin D 1000U BID | 80017748 | 90 | 180 | 0 | 0.09389 | 16.9002 | 9.100107692 | 0 | 26.00030769 | 9.100107692 | 0 | 26.00030769 |  |  |  |
| Nunavut |  |  |  |  |  |  |  |  |  |  | 220.881707 | 227.3830608 | 911.0027462 |  |  |  |
| Yukon | Metformin 1000mg BID | 2167786 | 90 | 360 |  | 0.0247 | 8.892 | 2.1127392 | 12.72 | 23.7247392 | 14.8327392 | 23.7247392 | 0 |  |  |  |
| Yukon | Atorvastatin 40mg daily | 2295296 | 90 | 90 |  | 0.2342 | 21.078 | 5.0081328 | 12.72 | 38.8061328 | 17.7281328 | 38.8061328 | 0 |  | Yukon /Annual costs |  |
| Yukon | Omeprazole 20mg daily | 2245058 | 90 | 90 |  | 0.2287 | 20.583 | 4.8905208 | 12.72 | 38.1935208 | 17.6105208 | 38.1935208 | 0 |  | Pharmacy Margin | 783.3462996 |
| Yukon | Irbesartan 300 mg daily | 2328100 | 90 | 90 |  | 0.2184 | 19.656 | 4.6702656 | 12.72 | 37.0462656 | 17.3902656 | 37.0462656 | 0 |  | Government share | 870.7538688 |
| Yukon | Levothyroxine 50 mcg daily | 2213192 | 90 | 90 |  | 0.03 | 2.7 | 0.64152 | 12.72 | 16.06152 | 13.36152 | 16.06152 | 0 |  | Patient share | 3566.444831 |
| Yukon | Atenolol 50mg daily | 2255545 | 90 | 90 |  | 0.1107 | 9.963 | 2.3672088 | 12.72 | 25.0502088 | 15.0872088 | 25.0502088 | 0 |  |  |  |
| Yukon | Liraglutide inj 1.8mg daily | 2351064 | 90 | 27 |  | 29.7367 | 802.8909 | 50 | 12.72 | 865.6109 | 62.72 | 0 | 865.6109 |  |  |  |
| Yukon | Lorazepam 1mg QHS | 655759 | 90 | 90 |  | 0.04 | 3.6 | 0.85536 | 12.72 | 17.17536 | 13.57536 | 17.17536 | 0 |  |  |  |
| Yukon | ASAEC 81mg daily | 2237726 | 90 | 90 |  | 0.08 | 7.2 | 1.71072 | 12.72 | 21.63072 | 14.43072 | 21.63072 | 0 |  |  |  |
| Yukon | Calcium 500mg / Vitamin D 1000U BID | 80017748 | 90 | 180 |  | 0.09389 | 16.9002 | 9.100107692 | 0 | 26.00030769 | 9.100107692 | 0 | 26.00030769 |  |  |  |
| Yukon |  |  |  |  |  |  |  |  |  |  | 195.8365749 | 217.6884672 | 891.6112077 |  |  |  |
